# The neighborhood of the Spike gene is a hotspot for modular intertypic homologous and non-homologous recombination in Coronavirus genomes

**DOI:** 10.1093/molbev/msab292

**Published:** 2021-10-12

**Authors:** Marios Nikolaidis, Panayotis Markoulatos, Yves Van de Peer, Stephen G Oliver, Grigorios D Amoutzias

**Affiliations:** 1 Bioinformatics Laboratory, Department of Biochemistry and Biotechnology, University of Thessaly, Larissa, 41500, Greece; 2 Microbial Biotechnology-Molecular Bacteriology-Virology Laboratory, Department of Biochemistry and Biotechnology, University of Thessaly, Larissa, 41500, Greece; 3 Department of Plant Biotechnology and Bioinformatics, Ghent University, Ghent, 9054, Belgium; 4 Center for Plant Systems Biology, VIB, Ghent, 9054, Belgium; 5 Department of Biochemistry, Genetics and Microbiology, University of Pretoria, Pretoria, 0028, South Africa; 6 College of Horticulture, Nanjing Agricultural University, Nanjing, 210095, China; 7 Department of Biochemistry, University of Cambridge, Sanger Building, 80 Tennis Court Road, Cambridge, CB2 1GA, UK

## Abstract

Coronaviruses (CoVs) have very large RNA viral genomes with a distinct genomic architecture of core and accessory open reading frames (ORFs). It is of utmost importance to understand their patterns and limits of homologous and non-homologous recombination, because such events may affect the emergence of novel CoV strains, alter their host range, infection rate, tissue tropism pathogenicity, and their ability to escape vaccination programs. Intratypic recombination among closely related CoVs of the same subgenus has often been reported; however, the patterns and limits of genomic exchange between more distantly related CoV lineages (intertypic recombination) needs further investigation. Here, we report computational/evolutionary analyses that clearly demonstrate a substantial ability for CoVs of different subgenera to recombine. Furthermore, we show that CoVs can obtain—through non-homologous recombination—accessory ORFs from core ORFs, exchange accessory ORFs with different CoV genera, with other viruses (i.e., toroviruses, influenza C/D, reoviruses, rotaviruses, astroviruses) and even with hosts. Intriguingly, most of these radical events result from double-crossovers surrounding the Spike ORF, thus highlighting both the instability and mobile nature of this genomic region. While many such events have often occurred during the evolution of various CoVs, the genomic architecture of the relatively young SARS-CoV/SARS-CoV-2 lineage so far appears to be stable.

## Introduction

Genomic analyses of single-stranded RNA-viruses, including Coronaviruses (CoVs), have repeatedly demonstrated how recombination affects their emergence, host-range, and pathogenicity (Decaro et al. 2009; Simon-Loriere and Holmes 2011; Terada et al. 2014; Tian et al. 2014; Su et al. 2016; Lau et al. 2018). Given the current pandemic of SARS-CoV-2 (Coronaviridae Study Group of the International Committee on Taxonomy of Viruses 2020; Wu et al. 2020), it is of utmost importance to fully understand the patterns and limits of homologous and non-homologous genomic exchange of the entire CoV subfamily. This knowledge will allow us to better evaluate any risks from cross-species transmission and recombination with other closely or distantly related viruses. It may also guide the development of future vaccines, by allowing the selection of stable antigenic regions and avoiding reversion (via recombination) of any future live-attenuated vaccine strains (Guillot et al. 2000; Racaniello 2006; Pliaka et al. 2012; Burns et al. 2013; Graham et al. 2018; Nikolaidis et al. 2019).

According to the ICTV 2020 release, the CoV subfamily (*Orthocoronavirinae*) harbours significant genomic diversity, comprising 4 genera (α–δ), further subdivided into 25 sub-genera (Lauber et al. 2012; Lauber and Gorbalenya 2012; ICTV Coronaviridae study group). Various CoVs are found in a wide range of animal species, causing respiratory, enteric, hepatic, and nervous system disorders with mild to severe symptoms (Rota et al. 2003; Weiss and Navas-Martin 2005; Woo et al. 2007; Bermingham et al. 2012; Wheeler et al. 2018; Chen et al. 2020; Wu et al. 2020). Bats are reservoirs for the α- and β-CoVs, whereas wild birds are reservoirs for the γ- and δ-CoVs (Woo et al. 2009; Woo et al. 2012; Wong et al. 2019; Latinne et al. 2020; Wille and Holmes 2020). Human CoVs are found in the α- and β-genera and have a zoonotic origin, with bats as the key reservoir, but intermediate hosts may also be involved in the cross-species transmission (Song et al. 2005; Reusken et al. 2013; Fan et al. 2019).

CoVs possess very large genomes among RNA-viruses (25-32 Kb) and contain at least 6 core ORFs (1a, 1b, Spike, Envelope, Membrane, and Nucleocapsid) (Gorbalenya et al. 2006; Cui et al. 2019; Chen et al. 2020). Lineage-specific accessory ORFs are also present and may be involved in host adaptation, including the modulation of interferon signaling and the production of pro-inflammatory cytokines (Gorbalenya et al. 2006; Liu et al. 2014; Cui et al. 2019; Hartenian et al. 2020). This large genome size and complex architecture allows division of labour and flexibility for cross-species adaptation (Lauber et al. 2013). Importantly, the Spike protein facilitates binding to host receptors and so determines host-range, cell-tropism, and even the transition from a mild towards a highly pathogenic phenotype, via point-mutations and recombination (Sánchez et al. 1999; Kuo et al. 2000; Casais et al. 2003; Rottier et al. 2005; Menachery et al. 2015).

Recombination events among closely-related CoV strains/genotypes/species of the same subgenus have been reported frequently (Keck et al. 1988; Kottier et al. 1995; Herrewegh et al. 1998; Decaro et al. 2009; Tian et al. 2014; Dudas and Rambaut 2016; Forni et al. 2017; Bobay et al. 2020; Boni et al. 2020; Saeng-Chuto et al. 2020; Goldstein et al. 2021; Yang et al. 2021); we denote this category of events as *intratypic* recombination. The corresponding recombination junctions are scattered across the genome, although enrichment around transcriptional regulatory sequences (TRS-B) has been reported (Yang et al. 2021). These TRS are needed for template switching during the transcription of the CoV ORFs (Sawicki et al. 2007; Sola et al. 2015), but they may also facilitate recombination via template switching among different CoVs (Graham et al. 2018; Yang et al. 2021). The genomes of several CoVs are mosaic, but many of their donors have yet to be sequenced (Goldstein et al. 2021). Furthermore, recombination events among more distantly related CoVs have also been observed. Such radical evolutionary events probably result from the presence of highly conserved TRS-B sequences (shared between the recombining CoVs) at the beginning of the various ORFs (Sawicki et al. 2007; Sola et al. 2015; Boniotti et al. 2016; Graham et al. 2018; Banerjee et al. 2020). Nevertheless, very disparate TRS-B sequences between two CoVs cause incompatibility and thus may also present barriers to such recombination events (Yount et al. 2006). In this study, we define as *intertypic* any recombination event among members of different CoV subgenera. In addition, non-homologous recombination events may occur with other viruses or taxa, leading to the acquisition of new genomic regions that appear as lineage-specific accessory ORFs (Zeng et al. 2008; Woo et al. 2014; Forni et al. 2017). The goal of this study is to understand the patterns and limits of radical (intertypic) genomic exchange of CoVs and to see whether any genomic regions emerge as hotspots of recombination. The first part of this analysis focuses on homologous recombination of core ORFs among different CoV subgenera, whereas the second part deals with non-homologous recombination of accessory ORFs among CoV subgenera/genera and even with other taxa.

## Results

Several computational methods exist for detecting and analyzing recombination events among closely related viruses (Posada et al. 2002; Pond et al. 2005; Martin et al. 2011). In this study, we have implemented phylogenetic tree incongruence methods, which are best suited for macro-evolutionary analyses, as well as similarity plots (see Methods Sections). BioNJ, PhyMl and Bayesian protein phylogenetic trees and tanglegrams (or 'cophylo plots', a way of graphically representing correspondence between two phylogenies with the same tip labels) were generated for the non-structural peptides (nsps) of ORFs 1a/1b and the other core ORFs. This was done both for all four genera together and for each of the four genera individually. In addition, phylogenetic trees (BioNJ and PhyML) of the various regions were compared against each other for incongruence, using the normalized Robinson-Foulds method for unrooted trees (see Methods section). We further validated the statistical significance of detected incongruities with CONSEL, to ensure the robustness of our conclusions. In this study, we only consider highly confident phylogenetic incongruence events that are supported by high bootstrap, aLRT and posterior probability values for all three tree methods and are also statistically supported by the corresponding CONSEL analyses. In all analyses, the neighborhood of the Spike ORF emerges as an intertypic recombination hotspot.

**The Spike ORF displays elevated phylogenetic tree incongruence**

Phylogenetic trees based on the Spike ORF consistently display the highest or next-highest phylogenetic incongruence compared to all other analyzed regions, in α-, γ- and δ-CoVs (Figure **1**; suppl. file 1 figs 32-33, 38-39, 50-51, 57-58). In contrast, the corresponding regions of β-CoVs display relatively low phylogenetic incongruence. The Spike sequence is one of the most variable core genomic regions. However, other core regions also have similar sequence variability, but do not display such high levels of phylogenetic incongruence. Therefore, this pattern (confirmed by subsequent phylogenetic tree tanglegram analyses) does not result from badly aligned regions, rather, it may be attributed to divergence combined with cassette-like intertypic recombination. If the majority of intertypic recombination events involved single crossovers, then there should be high phylogenetic incongruence among the regions flanking the Spike ORF, but this is not the case. Furthermore, if most of the intertypic recombination (in various regions) involved single crossovers, then the incongruence among the 5' terminal nsps and the 3' terminal ORFs, such as Membrane and Nucleocapsid, should also be high, resembling linkage disequilibrium decay (Dudas and Rambaut 2016), but it is not.

**Tanglegram-based detection of intertypic recombination events in the common ancestors of CoV genera and subgenera**

α- and β-CoVs consistently cluster together as a major clade for all core genomic regions except for Spike, for which most of the α- and all the δ-CoVs form a single group (Figure **2** and suppl.fig.1, recombination event 21). Moreover, cryo-electron microscopy has demonstrated that the Spike proteins of α- and δ-CoVs are structurally more similar to each other (Shang et al. 2018). Thus, at least one recombination event occurred in which the common ancestor of all δ-CoVs obtained a Spike ORF from an α-CoV ancestor.

We also observed several cases of phylogenetic incongruence involving entire subgenera (mostly in α-CoVs); they displayed a major shift in their phylogenetic position (for a certain genomic region), as a monophyletic group. We interpret this as a major event that occurred in the common ancestor of the representative sequences of that subgenus. Here, we only report cases well supported by BioNJ, PhyML and Bayesian tree tanglegrams and also statistically supported (for their incongruence) by CONSEL. The regions that are involved in such events are shown in Figure **2** and are designated as SgM (Subgenus Movement).

More specifically, in α-CoVs, there exist 14 well-established subgenera, with the *Ozimops* and *Desmodus* genomes possibly forming two extra subgenera. The first 9 subgenera (*Decacovirus, Pedacovirus, Colacovirus, Nyctacovirus, Minunacovirus, Duvinacovirus, Setracovirus, Myotacovirus, Rhinacovirus*) together with *Ozimops* and *Desmodus* constitute a major clade that we designate A1. Another two subgenera, (*Tegacovirus, Minacovirus*) constitute a major clade that we designate A2 and is a sister group to A1. *Luchacovirus* (found in rodents), *Sunacovirus* and *Soracovirus* (both found in shrews) constitute three very diverse additional clades, that we designate A3, A4 and A5 respectively. The tanglegrams reveal that *Ozimops* is a sister group to *Decacovirus*, but for nsp16 it pairs with *Minunacovirus* (recombination event 4, suppl.figs.16-19). The *Rhinacovirus* (A1 clade) nsp8 is no longer part of the A1 clade, but clusters with the A3 *Luchacovirus* (recombination event 3, suppl.figs.12-15). *Luchacovirus* (A3 clade), moves within the A1 clade for both nsp1 (recombination event 1, suppl.figs.4-7) and nsp7 (recombination event 2, suppl.figs.8-11). Similarly, *Sunacovirus* (A4 clade) moves within the A1 clade for Envelope (recombination event 11, suppl.figs.28-31). We observed many other incongruities for most of the subgenera in various genomic regions, but their new positions (in the trees) were not supported by both high bootstrap/aLRT values and different trees, thus they may actually represent cases of rapid divergence.

Although α-CoVs form 5 distinct lineages, their Spike ORFs are organized into two major evolutionary clusters. The smaller cluster comprises *Rhinacovirus* (a member of clade A1), *Luchacovirus* (clade A3), *Sunacovirus* (clade A4), *Soracovirus* (clade A5), whereas the major cluster comprises all the other members of clades A1 and A2 (see Spike tree in Figure **2**, recombination events 5, 8, 9, 10 in suppl.figs.1-2, 20-23,). The Spike ORF of this smaller cluster has been suggested to originate from β-CoVs via an ancient recombination event (Tsoleridis et al. 2019).

Phylogenetic incongruence was also observed for the Nucleocapsid region of β-CoV *Merbecovirus* (Figure **2** and recombination event 12 in suppl.figs.34-37). By taking *Sarbecovirus* as the reference point, *Hibecovirus* is their closest subgenus, followed by *Nobecovirus*, *Merbecovirus,* and finally *Embecovirus* (most distant). The only exception to this pattern is observed in the Nucleocapsid region, where *Merbecovirus* seems to be the closest subgenus to the *Sarbecovirus-Hibecovirus* group. An alternative explanation is that the ancestral *Nobecovirus* Nucleocapsids underwent recombination or significant sequence divergence. However, manual inspection of the trees, their branch lengths, and the Poisson-distances leads us to favor the first explanation, whilst acknowledging that the second cannot be excluded at present.

**Tanglegram-based detection of intertypic recombination between some members of different subgenera**

We investigated instances where certain genomic regions of the members of a particular subgenus did not form a monophyletic group. These observations could be attributed to rapid divergence or intertypic recombination events in some, but not all, members. These events are more recent than the ones (described above) that occurred in the common ancestor of a subgenus. Such regions are shown in Figure **2** (designated as "P": polyphyletic). We checked whether these candidate recombinant sequences clustered within or next to other subgenera with high bootstrap/aLRT/posterior probability values and also performed similarity plot and bootscan analyses with RDP4 (Martin et al. 2015) (see Methods), whenever possible. We detected several events; two in α-CoVs, five in γ-CoVs, and three in δ-CoVs. Interestingly, 9 of these 10 events are located at the Spike ORF.

The most striking and recent event has been documented for Swine Enteric CoV (Boniotti et al. 2016), which is essentially a swine *Tegacovirus* (A2 lineage) that obtained the Spike ORF of a swine *Pedacovirus* (A1 lineage) (recombination event 6, suppl.figs.20-22, 24-25). A second case (again in the Spike ORF) concerns five of the thirteen analyzed *Tegacovirus* sequences that form a monophyletic sister group to *Minacoviruses* (recombination event 7, suppl.figs.20-22, 26-27). An alternative sequence of events is that the other seven *Tegacovirus* (from cats and dogs) that form the second Spike monophyletic group recombined with an as yet unknown donor from the A2 lineage. Inspection of the phylogenetic trees and their branches leads us to favor the first option, while the host-range of the second group favors the second option. Yet another instance concerns four γ-CoV *Igacovirus* Spike sequences (from birds) that form a monophyletic cluster outside of the *Igacovirus* (recombination events 13-16, suppl.figs.40-44). This is a case of three or most probably four independent events where members from an as yet unknown γ-CoV subgenus repeatedly served as Spike donors to several Igacoviruses. A further case involves a duck *Igacovirus* Membrane sequence that clusters with the γ–CoV *Brangacovirus* (recombination event 17, suppl.figs.45-49). A final example concerns five δ-CoV *Buldecovirus* Spike sequences forming a monophyletic cluster (that is outside of Buldecoviruses) and is a sister group to *Herdecovirus* (recombination events 18-20, suppl.figs.52-56). Our interpretation is that this is a case of three independent events, where members from an, as yet unknown, δ-CoV subgenus (a close relative of Herdecoviruses) repeatedly served as Spike donors to these Buldecoviruses.

In addition, we detected several low-confidence intertypic recombination events for α-CoV subgenera, where the incongruent sequences cluster with other subgenera, but with low bootstrap/aLRT/posterior probability support. Here, either the donor is unknown or the incongruence is due to rapid divergence; they were not considered further in our study. Finally, we also observed previously reported intratypic recombination events, i.e. within *Sarbecovirus* (Suppl.Figs.60-68). Although such events are not the focus of this study, it should be mentioned that, at the beginning of the COVID-19 pandemic, several studies analyzed the available genomic data for evidence of recombination that could have led to the emergence of SARS-CoV2 (Boni et al. 2020; Lam et al. 2020; Paraskevis et al. 2020; Yang et al. 2021). Although the data show that SARS-CoV2 did not emerge via a recent recombination event, recombinant sequences (from other species) among the SARS-CoV and SARS-CoV2 lineages have been detected and were also confirmed by our study.

**Accessory ORF evolution: Non-homologous recombination of accessory ORFs between different CoV subgenera and genera.**

Based on PSI-BLAST, we built position-specific scoring matrices (PSSMs) for the various annotated accessory ORFs and thus identified 73 non-redundant Accessory ORF Families (AOFs; see Methods Section). The PSSMs allowed for a very sensitive homology search and revealed very distinct distributions in the various genera and subgenera (Figure **3**, Figure **4** and suppl.file

2). Although no AOF was present in all four genera, three AOFs were present in some subgenera of both α- and β-CoVs and three AOFs were present in subgenera of both γ- and δ-CoVs. Interestingly, three of these intergenus AOFs are localized in the neighborhood of the Spike ORF. Possibly, some AOFs with restricted distributions may actually be distant homologs of other AOFs that significantly diverged (Ouzounis 2020; Neches et al. 2021) and lost their homology signal.

Intriguingly, we detected two AOFs with very restricted distributions that originated either from gene duplication or horizontal gene transfer (HGT) of a Spike ORF fragment. The first instance concerns a bat β-CoV *Hibecovirus* ORF2 that is situated between ORF1ab and Spike, that is distantly homologous to the N-terminal region of its Spike (suppl.file 2: PSSM_TBlastN: 4e-39; 27% identity). This is either a case of non-homologous recombination/gene-fragment duplication within the same genome (followed by rapid divergence) or horizontal transfer from another related *Hibecovirus* Spike N-terminal region. The second instance concerns a similar Spike gene-fragment duplication event for ORF6 of some Luchacoviruses (suppl.file 2: PSSM_TBlastN: 7e-63; 25% identity).

We also detected distant homology between the ORF3a of β-CoV *Sarbecovirus/Hibecovirus/Nobecovirus* and the Membrane ORF of α-CoV A2 *Tegacovirus* and A4 *Sunacovirus* (suppl.file 2: PSSM_TBlastN: 2.4e-4 and 3.9e-4 respectively). Accordingly, a bioinformatics analysis (Ouzounis, 2020) recently reported a very distant homology among the SARS-CoV-2 ORF3a and Membrane ORFs. Based on our extended genome sampling and the observed e-values of the ORF3a PSSM against α–CoVs (best PSSM_TBlastN: 2.4e-4) and β-CoVs (best PSSM_TBlastN: 2e-3), possibly a Membrane region from α-CoVs jumped via non-homologous recombination to the common ancestor of *Sarbecovirus/Hibecovirus/Nobecovirus* and rapidly diverged to an accessory ORF.

**Non-homologous recombination of accessory ORFs between coronaviruses and other taxa**

We detected seven AOFs that had homologs in other taxa, outside of the *Coronavirinae* (suppl.file 2), with three of them situated in the neighborhood of Spike. The most striking and well-studied example is a hemagglutinin-esterase (MHV_HE) that is present in all the members of β-CoV *Embecovirus*, situated just before the Spike. It has homologs in toroviruses (porcine torovirus PSI-Blast e-value: 1.7e-55) and influenza C/D. Most probably, it was acquired either indirectly (via a torovirus intermediate step) or directly from an influenza C/D-like virus, and subsequently adapted and coevolved with the Spike (Snijder et al. 1991; Zeng et al. 2008; Caprari et al. 2015; Lang et al. 2020).

Another case is the β-CoV NS2 *Embecovirus* AOF (MHV_NS2), that belongs to the 2H phosphoesterase superfamily (Mazumder et al. 2002). This AOF is observed in most Embecoviruses, like HCoV-OC43, and is situated between ORF1ab and the hemagglutinin-esterase (HE). Interestingly, close homologs (NCBI-BlastP e-value: 6e-61) of this AOF (from β-CoVs) are consistently found in several rodent α-CoV Luchacoviruses as well (Tsoleridis et al. 2019), at the same genomic location, but they do not have the neighboring HE ORF. This AOF is also homologous to a region within the central part of polyprotein 1ab of several toroviruses, including porcine torovirus (PSI-BLAST e-value: 2e-28). Apparently, non-homologous genomic exchange among CoVs and toroviruses has happened more than once.

Next to the α-CoV *Luchacovirus* ORF2/NS2, there exists another accessory ORF (instead of HE in Embecoviruses), designated ORF2b. It is present in some, but not all α-CoV Luchacoviruses. It is homologous to rodent C-type lectins (PSI-Blast e-value: 4e-34) found in natural killer cell receptors as well as in many poxviruses and some herpesviruses. This AOF probably originated from its hosts (Wang et al. 2020). Furthermore, both ORF2a and ORF2b are missing from another closely related *Luchacovirus* genome (MT820625.1), thus highlighting the dynamic nature of this genomic region (Wang et al. 2020) and the potential for gene loss (Forni et al. 2017).

We also identified four more interesting AOFs. p10, situated just after the nucleocapsid region of some β-CoV Nobecoviruses in bats, is homologous (PSI-BLAST e-value: 3.9e-22) to p10 proteins from reoviruses (Huang et al. 2016). The *Buldecovirus* NS7a AOF (situated after the Nucleocapsid) of several avian δ-CoV Buldecoviruses is homologous (PSI-BLAST e-value: 1e-10) to NSP1-1 from avian rotavirus-g. An uridine kinase (closest PSI-Bast hit: fungi; e-value: 2e-30) is found only in γ-CoV Cegacoviruses (Mihindukulasuriya et al. 2008). Finally, the same γ-CoV Cegacoviruses contain ORF6 that is distantly homologous to the capsid protein of human astrovirus 5 (PSI-BLAST e-value: 4.7e-7).

## Discussion

The integration of our extensive phylogenetic and genome architecture analyses have revealed intertypic homologous and non-homologous recombination events among the genomes of different CoV subgenera/genera, and even with other taxa. Intriguingly, many of these events are localized around the Spike ORF and occur as double crossovers, where an entire region is exchanged as a cassette/module and the rest of the genome stays intact. It is unlikely that these observed and statistically supported phylogenetic incongruities (especially for Spike) are artifacts of rapid divergence or convergent evolution, because the "incongruent" regions actually cluster with regions from other genera/subgenera with high bootstrap/aLRT/posterior probability support (among other evidence, like site-wise likelihood of alternative hypotheses – results not shown). The Spike recombination of Swine Enteric CoV is the most recent and clear example. We have applied stringent analysis criteria involving the phylogeny of entire regions and it is possible that many genuine intertypic recombination events may not have passed our filters, especially if they involved small segments of an ORF (Forni et al. 2017). Another major problem is genomic sampling, where the donor has yet to be sequenced (Goldstein et al. 2021).

Our interpretation for the frequently observed modular recombination events around the Spike ORF is that long-range genetic interactions of various genomic regions may actually block radical (intertypic) single crossover recombination events (Sola et al. 2011; Sola et al. 2015) but allow for double crossover events in certain genomic islands. This conclusion is supported by various independent experimental observations. Nucleocapsid proteins (N-proteins) from different members of the same genus may only be partially compatible, whereas N-proteins from different genera are completely incompatible (Schelle et al. 2005; Sungsuwan et al. 2020) and may even have a suppressive effect (Masters 2019; Sungsuwan et al. 2020). N-proteins are also involved in circularization of the genome (Lo et al. 2019). CoV RNA secondary structures have been shown to form long-range interactions within a CoV genome (Ziv et al. 2020) and to interact with cellular components, to initiate transcription and replication (Sola et al. 2011). Genetic interactions have been observed between the nsp8, nsp9 peptides (from ORF1a) and the pseudoknot at the 3' end of the genome (Züst et al. 2008). Thus, single-crossover recombination events among different subgenera may break such long-range interactions, while double-crossover/modular events may allow their retention.

We also observed distinct subgenus-specific accessory ORF genomic architectures. These may function as an additional barrier to single-crossover intertypic recombination events, that would otherwise disrupt certain co-evolved combinations of ORFs. Several of these AOFs have been introduced from other genera/subgenera. However, some of these AOFs do not have homologs in any other subgenera and may have emerged via i) *de novo* gene birth, ii) rapid divergence of existing ORFs and loss of the homology signal, or iii) via non-homologous recombination with ORFs (followed by rapid divergence) from other CoVs, other viruses, or even hosts (Elhaik et al. 2006; McLysaght and Hurst 2016; Moyers and Zhang 2016; Schmitz and Bornberg-Bauer 2017; Ouzounis 2020).

We observed exchange of genomic regions between CoVs and toroviruses, influenza C/D (directly or indirectly), reoviruses, rotaviruses, astroviruses, and even with hosts. Such events were frequent in the neighborhood of the Spike ORF. Toroviruses are of particular interest, because they belong to the same order (*Nidovirales*) as CoVs and can also act as gene donors in other viral orders, e.g. porcine *Enterovirus*-G (Shang et al. 2017; Hu et al. 2019). Worryingly, porcine toroviruses have both a worldwide distribution and a high infection rate (Hu et al. 2019). Thus, future genomic sampling of yet undiscovered CoVs may reveal an even more extensive exchange between CoVs and toroviruses. Moreover, genomic exchange between viruses (*Flaviviridae, Hepeviridae, Dicistroviridae, Potyviridae*) and their hosts has been observed repeatedly (Gilbert and Cordaux 2017). It is conceivable that some of the above-mentioned CoV AOFs did not move from one virus to the other, but independently from similar hosts; however, the PSI-Blast results show other viral sequences, and not cellular proteins, to be the closest hits.

Importantly, members of the relatively young (Boni et al. 2020) SARS-CoV/SARS-CoV-2 lineages (within Sarbecoviruses) do not yet appear to act as recipients in radical intertypic recombination events. They also display a very distinct AOF architecture. Thus, current evolutionary data do not favor a scenario where SARS-CoV-2 may (homologously) recombine with other currently circulating human CoVs of other subgenera/genera. Furthermore, SARS-CoV/SARS-CoV-2 do not seem to exchange accessory ORFs with other CoV subgenera or other viruses/hosts, with the exceptions of ORF3a that is an old and unresolved event and ORF7a (with some Decacoviruses). It should be noted that their closest relatives, Hibecoviruses, have a divergent Spike-like accessory ORF that resulted from either a gene duplication or horizontal transfer event. Nevertheless, SARS-like viruses can recombine with SARS2-like viruses, as our and other analyses have shown (Boni et al. 2020; Lam et al. 2020; Yang et al. 2021). This finding has very important implications, because, combined with the ability of Sarbecoviruses to easily move from one host to another, it demonstrates a potential for a future intratypic recombination event (within Sarbecoviruses), where a highly infectious SARS-CoV2 variant (e.g. the Delta variant) could recombine with a SARS-like sequence in another host species and give rise to a recombinant that combines the high infectivity of SARS-CoV2 with the much higher mortality rate of SARS itself.

Many of the events that we have observed are very old; nevertheless, our results suggest that researchers and those responsible for public health should be vigilant. Certain key taxa like bats and/or farmed animals (especially pigs) have the potential to play a key role in any future emergence of a recombinant SARS-CoV-2 strain or some other CoV epidemic (from another genus/subgenus). SARS-CoV-2 spill-back from humans to other animals (domesticated or wild) that also harbor many and diverse CoVs has been reported (de Morais et al. 2020; Olival et al. 2020; Sit et al. 2020). Ferrets, cats and dogs are susceptible to the currently circulating SARS-

CoV-2 strains, whereas pigs, chicken and ducks appear to have lesser, or no susceptibility (Meekins et al. 2020; Shi et al. 2020; Sit et al. 2020; Pickering et al. 2021). CoVs demonstrate a high capacity for cross-species infection, even from birds to mammals, either directly or via a few evolutionary steps (Li et al. 2006; Graham and Baric 2010; Menachery et al. 2015; Menachery et al. 2016; Li et al. 2018; Boley et al. 2020). Furthermore, pigs are carriers of very diverse α-, β-, as well as δ-CoVs and have been shown to function as "recombination bioreactors", with the notable example of Swine Enteric CoV (Boniotti et al. 2016). In addition, intensively farmed pigs are hosts for many other viruses, such as toroviruses or influenza A (Hu et al. 2019; Henritzi et al. 2020; Sun et al. 2020). Fortunately, genomics is a valuable new tool for monitoring the emergence, spread, and ongoing adaptations of SARS-CoV-2 (Boni et al. 2020; Neches et al. 2020; Worobey et al. 2020; Kemp et al. 2021; Volz et al. 2021). It is conceivable that what we have observed is only the "tip of the iceberg"; that past unknown recombination events of various CoVs may have led to many unnoticed (or, perhaps, readily contained) localized small-scale epidemics that died out. However, given the observed genomic diversity and inherent genomic instability of CoVs, in this new era of urbanization, global transport, intensive farming, and habitat destruction (Beyer et al. 2021), intratypic and intertypic recombination events may lead to new epidemic strains that may prove much more difficult to contain (Bedford et al. 2019). As a final note, these results highlight the need to further investigate the inclusion of other, and much more stable, genomic regions (in addition to Spike) in the design and development of the next generation of coronavirus vaccines.

## Methods

### Phylogenetic analyses

We obtained the taxonomy IDs for α-, β-, γ- and δ-CoVs from NCBI Taxonomy in order to search for available nucleotide sequences in Genbank (Benson et al. 2013), using (as two extra criteria) the keyword "complete" and nucleotide length higher than 24,000. We obtained 1102, 14769, 435 and 154 genomic sequences from α-, β-, γ- and δ-CoVs respectively, in August 2020. Redundancy with the set of retrieved sequences was removed with the UCLUST software (Edgar 2010), using 90% nucleotide identity and 98% query coverage at the whole-genome level, in order to filter out the thousands of available genomes from the same virus that have been involved in large outbreaks, like SARS-CoV-2, PEDV, IBV. From each non-redundant group, we retained one representative sequence, or more if they were obtained from different hosts. We designate these groups as NRG90 (Non-Redundant-Group-90% nucleotide sequence identity). In addition, within each NRG90 group we ensured that we retained the representative RefSeq sequences for each species, that were obtained from ICTV taxonomy (ICTV Coronaviridae study group). Sequences were aligned with Muscle (Edgar 2004) and MAFTT (parameters: --auto) (Nakamura et al. 2018). Multiple alignment views and manual editing were performed with the Seaview4 software (Gouy et al. 2010). The boundaries of nsps within ORF1ab, as well as those of Spike, Envelope, Membrane, Nucleocapsid and the accessory ORFs were determined based on Genbank annotation and from manual inspection of the multiple alignments. Filtering of poorly aligned regions was performed with the g-blocks software (Castresana 2000), where we retained sites with less than 50% gaps and blocks of two consecutive sites. Model selection for ML and Bayesian trees was performed with Prottest3 (Darriba et al. 2011). Subsequent ML tree reconstruction was performed with PhyML (Guindon and Gascuel 2003) (applying SH-like approximate likelihood ratio test, SPR algorithm for tree search). Neighbour-Joining (BioNJ) trees were generated with Seaview4 (Gouy et al. 2010), using the Kimura two-parameter and Poisson models with 500 bootstraps, for nucleotide and protein sequences, respectively. Bayesian phylogenetic trees were calculated using the BEAST software v.1.10.4 (Drummond et al. 2012; Suchard et al. 2018) with MCMC length of 1 million and a burn-in value of 10000 (all the other operators and priors were set to default). Phylogenetic trees were visualized with Treedyn (Chevenet et al. 2006), iTOL (Letunic and Bork 2019) and Dendroscope (Huson and Scornavacca 2012). Phylogenetic trees were generated for all regions (nsps, ORF1ab, Spike, Envelope, Membrane, Nucleocapsid) of each CoV genus independently. In addition, phylogenetic trees that included all sequences of all four CoV genera together were generated for those regions (nsps 3-10, 12-16, ORF1ab, Spike, Membrane, Nucleocapsid) whose multiple alignments had a sufficient number of columns, after g-blocks filtering.

Phylogenetic tree incongruence was estimated/quantified with the Robinson-Foulds (RF) method (Robinson and Foulds 1981) for unrooted trees, within the Visual Treecmp server (Goluch et al. 2020). A certain genomic region is considered incongruent when its phylogeny is not in agreement with the phylogeny of the other regions (from the same genome). Visualization of the triangular matrix of Robinson-Foulds normalized values among the various trees was performed with Python and R pheatmap packages. This RF-matrix resembles the Linkage-Disequilibrium matrix, at the macroevolutionary level, but for specified genomic regions. Since the goal was to investigate incongruence at the macroevolutionary level and not within the virus species level, for this type of analyses, branches with length less than 0.02 were collapsed with the TreeGraph v2 software (Stöver and Müller 2010). Otherwise, the incongruence of strains of the same virus species would artificially inflate the RF values. This would especially be the case for γ-CoVs, where many divergent strains of IBV (*Igacovirus*) were available. Phylogenetic tree tanglegrams were visualized with Dendroscope (Huson and Scornavacca 2012), using the ML, BioNJ and Bayesian tree of ORF1ab as the reference tree against each of the ML, BioNJ and Bayesian trees of the individual nsps and ORFs S, E, M, N, for each of the four CoV genera separately. Estimation of evolutionary distance among homologous aligned sequence regions (for visualization in the RF-matrices) was performed with the Poisson-distance method within the MEGA X software (Kumar et al. 2018) (parameters - gap missing data: pairwise deletion; rates among sites: uniform). The statistical significance of phylogenetic incongruence of specific suspected recombination events was further assessed with the approximately unbiased (AU) test, using CONSEL (Shimodaira and Hasegawa 2001). For a certain set of sequences, the reference PhyML tree was obtained from the suspected recombined region and it was compared against the PhyML tree of the corresponding ORF1ab regions.

**Accessory ORF analysis**

In the first step of this analysis, all annotated accessory ORFs from our non-redundant set of 196 CoV genomes were retrieved from Genbank. We only retained accessory ORFs with a length ≥50 amino acids, with the exception of human CoVs (length≥30) that were situated in the regions among the 6 core ORFs and not any accessory ORFs that were entirely overlapping with any of the core ORFs. The selected annotated accessory ORFs in all analyzed genomes were further clustered in 88 homologous groups, using as cut-off, pairwise BLASTP e-values of 1e-10, followed by grouping with mcl-clustering (Enright et al. 2002). Afterwards, a representative peptide sequence from each cluster was used to build a corresponding Position Specific Scoring Matrix (PSSM) with locally installed PSI-BLAST, against the *Coronaviridae* proteins of the (locally installed) NCBI non-redundant protein database, with an e-value cut-off 1e-3 and as many iterations as needed, until convergence was achieved. Next, 15 redundant PSSMs were removed and we ended up with 73 annotated accessory ORF PSSMs. Accordingly, each non-redundant PSSM corresponded to one homologous **A**ccessory **O**RF **F**amily (AOF). All 73 PSSMs are available in supplementary file 3.

Afterwards, each AOF PSSM was used to scan all the analyzed CoV representative genomes for the presence of the corresponding family with TBlastN (cutoff: 1e-3). Each TBlastN hit was inspected to determine whether it encoded a peptide of at least 30 amino acids, otherwise it was considered to be pseudogenized (represented with orange colour in the matrices of figures 3 and 4). The coordinates of the detected homologous regions were visualized in each genome with Biopython and the genomic architectures were manually inspected. Genomic regions from the representative CoV genomes containing a certain AOF were aligned with Muscle. Each multiple alignment is available within the zipped supplementary file 4. Next, the annotated ORF PSSMs were used as queries to scan the entire NCBI non-redundant protein database, in order to detect AOF homologs in taxa outside of *Coronavirinae* and thus detect potential non-homologous recombination events (horizontal gene transfers). Intriguingly, bacterial draft genomes were found to include CoV AOFs with very high sequence identity. These draft genomes were re-assembled with Spades (Bankevich et al. 2012) and the relevant contigs were manually investigated for co-presence of CoV and bacterial genes, but they eventually appeared to be contaminations and were not further investigated.

## Figures

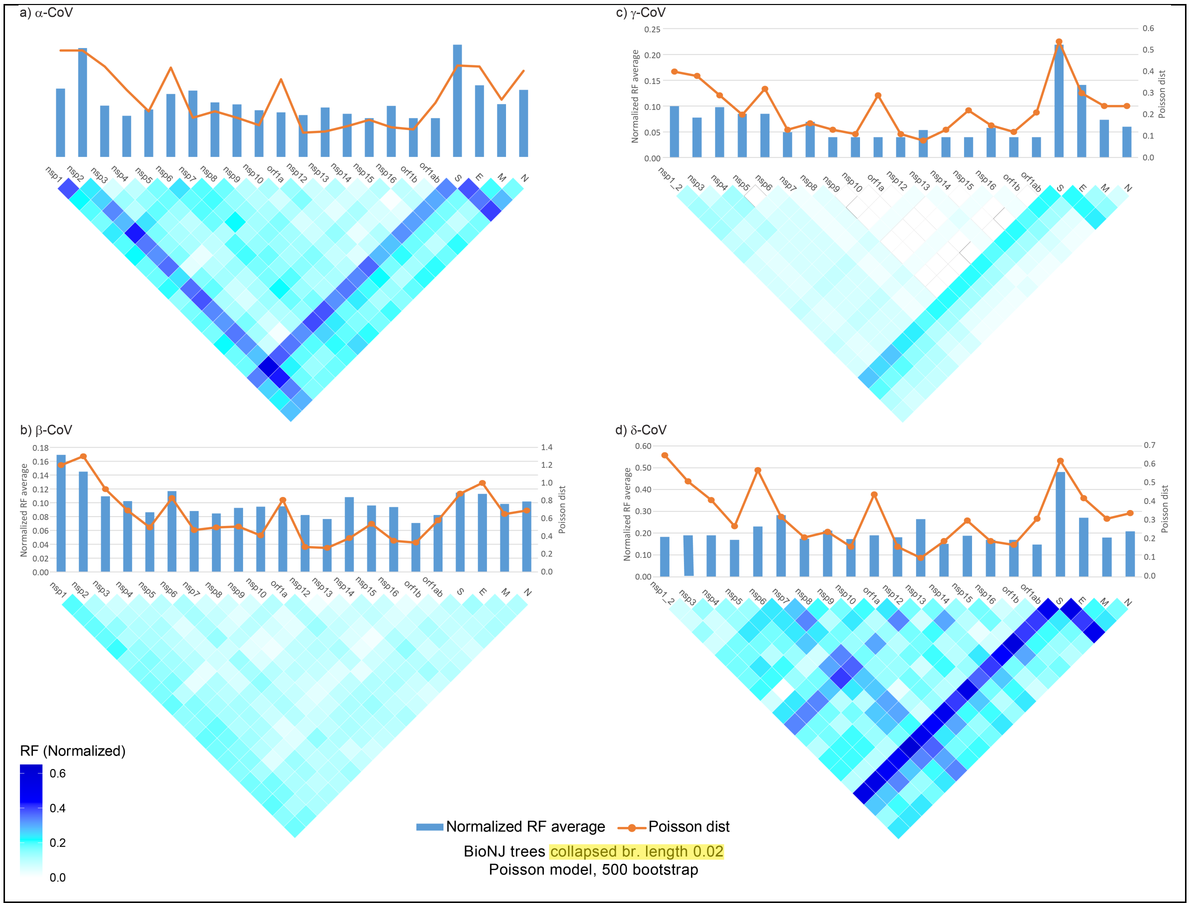

Figure 1. Matrices of incongruence among the core genomic regions of the four CoV genera based on the normalized Robinson-Foulds method, for unrooted trees (calculated with the TreeCMP server). BioNJ phylogenetic trees were generated with the Poisson model of evolution and 500 bootstrap replicates. In addition, branch lengths < 0.02 were collapsed. The orange line above each matrix displays the average Poisson-distance among sequences of the same genomic region (calculated with the MegaX software). Blue bars above each matrix display the average RF value for that particular region (against all other regions).

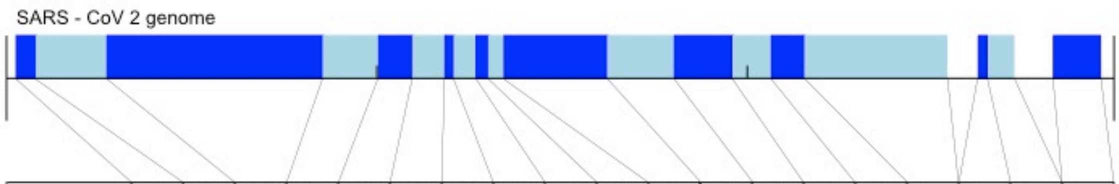

| | nsp1 | nsp2 | nsp3 | nsp4 | nsp5 | nsp6 | nsp7 | nsp8 | nsp9 | nsp10 | nsp12 | nsp13 | nsp14 | nsp15 | nsp16 | S | E | M | N |
|---|---|---|---|---|---|---|---|---|---|---|---|---|---|---|---|---|---|---|---|
| Decacovirus | | | | | | | | | | | | | | | | | | | |
| Ozimops | | | | | | | | | | | | | | | SgM | | | | |
| Minunacovirus | | | | | | | | | | | | | | | | | | | |
| Nyctacovirus | | | | | | | | | | | | | | | | | | | |
| Pedacovirus | | | | | | | | | | | | | | | | | | | |
| Colacovirus | | | | | | | | | | | | | | | | | | | |
| Myotacovirus | | | | | | | | | | | | | | | | | | | |
| Duvinacovirus | | | | | | | | | | | | | | | | | | | |
| Setracovirus | | | | | | | | | | | | | | | | | | | |
| Desmodus | | | | | | | | | | | | | | | | | | | |
| Rhinacovirus | | | | | | | | SgM | | | | | | | | SgM | | | |
| Tegacovirus | | | | | | | | | | | | | | | | P | | | |
| Minacovirus | | | | | | | | | | | | | | | | | | | |
| Luchacovirus | SgM | | | | | | SgM | | | | | | | | | SgM | | | |
| Sunacovirus | | | | | | | | | | | | | | | | SgM | SgM | | |
| Soracovirus | | | | | | | | | | | | | | | | SgM | | | |
| Sarbecovirus | | | | | | | | | | | | | | | | | | | |
| Hibecovirus | | | | | | | | | | | | | | | | | | | |
| Nobecovirus | | | | | | | | | | | | | | | | | | | |
| Merbecovirus | | | | | | | | | | | | | | | | | | | SgM |
| Embecovirus | | | | | | | | | | | | | | | | | | | |
| Igacovirus | | | | | | | | | | | | | | | | P | | P | |
| Brangacovirus | | | | | | | | | | | | | | | | | | | |
| Cegacovirus | | | | | | | | | | | | | | | | | | | |
| Buldecovirus | | | | | | | | | | | | | | | | GM / P | | | |
| Herdecovirus | | | | | | | | | | | | | | | | GM | | | |
| Andecovirus | | | | | | | | | | | | | | | | GM | | | |

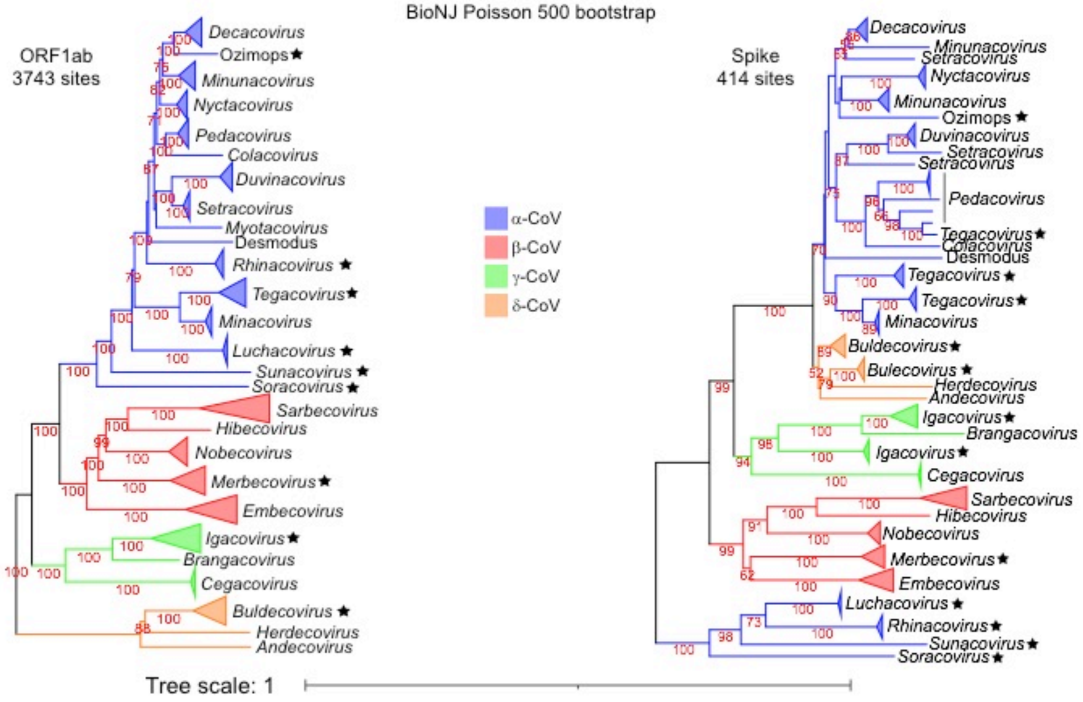

Stars indicate the CoV subgenera that are implicated in recombination events throughout the analyses

Figure 2. The genomic organization of the core ORFs and peptides of the SARS-CoV2 genome are displayed on the top of the figure. The table/matrix below it shows which genomic regions of the various subgenera are involved in intertypic recombination events. "GM" represents events that occurred at the common ancestor of the genus. "SgM" represents events that occurred at the common ancestor of the subgenus. "P" represents more recent events that occurred for one or few members of the subgenus and have resulted in a polyphyletic tree pattern (for that region and subgenus). All incongruence events in the matrix are supported by the three phylogenetic tree methods (NJ, PhyML, Bayesian) and are also statistically significant, based on the approximately unbiased test of CONSEL. Two phylogenetic trees (of ORF1ab and Spike) for all four genera are also included below the matrix, to visualize the recombination events of the Spike region. In these trees, we use stars to denote sub-genera that have been involved in intertypic homologous recombination events, in any genomic region (not only the Spike).

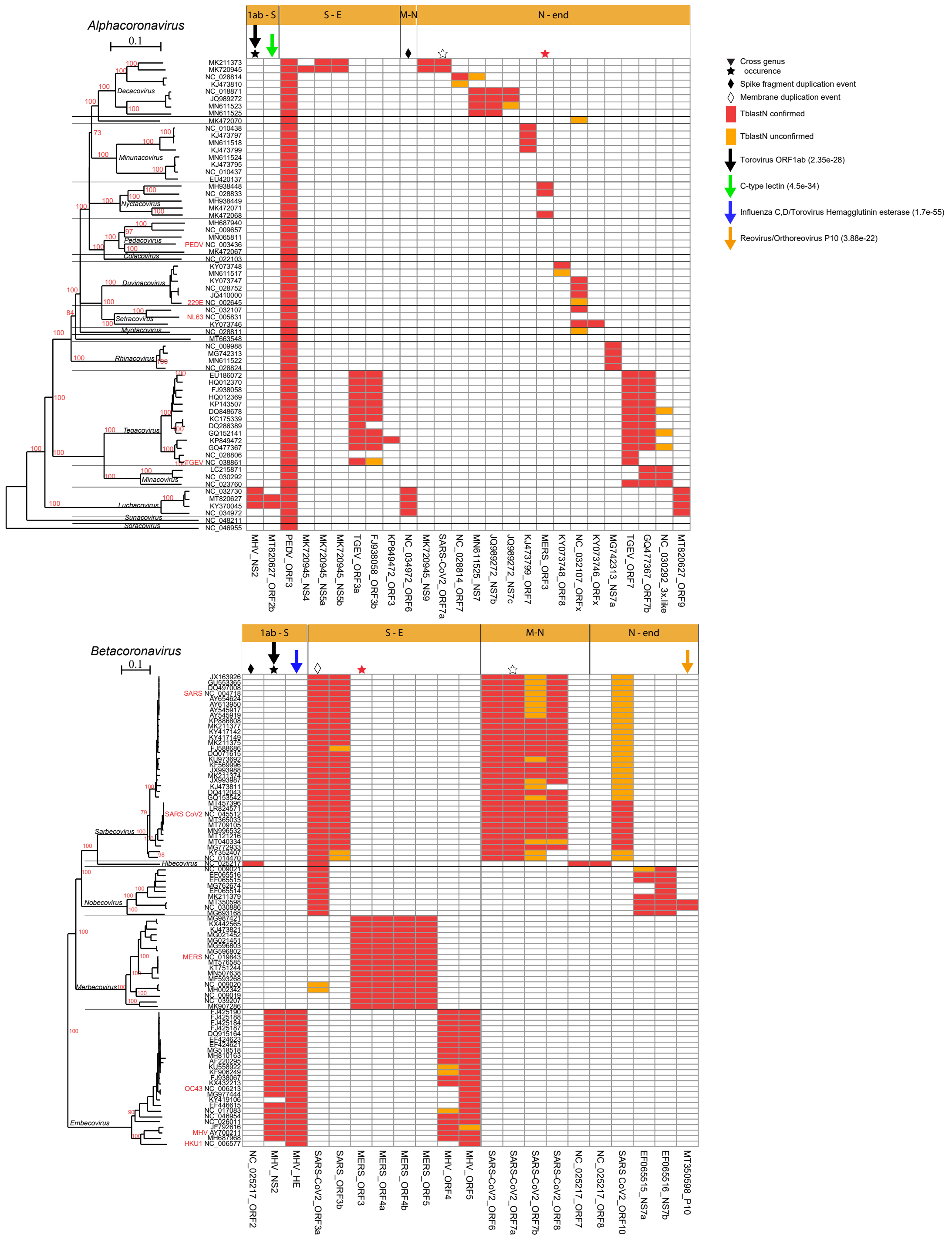

Figure 3. Presence and distribution of Accessory ORF Families (AOFs) in the α- and β-CoVs. Each column in the matrix represents a certain AOF. Red color (within the matrix cells) denotes the (TblastN) presence of an AOF that is also verified by a predicted ORF with length ≥30 aa, whereas if the length of the predicted ORF is <30 aa, then it is denoted with orange color. Stars denote AOFs that are present in both α- and β-CoV members, whereas diamonds denote an AOF that resulted from duplication of a core ORF. Downward arrows denote AOFs that have homologs in non-CoV genomes, together with their best PSI-Blast hit e-value. Horizontal orange bars (above the matrices) denote the genomic region where the AOF is located, i.e. S-E denotes the region between the Spike and Envelope ORFs.

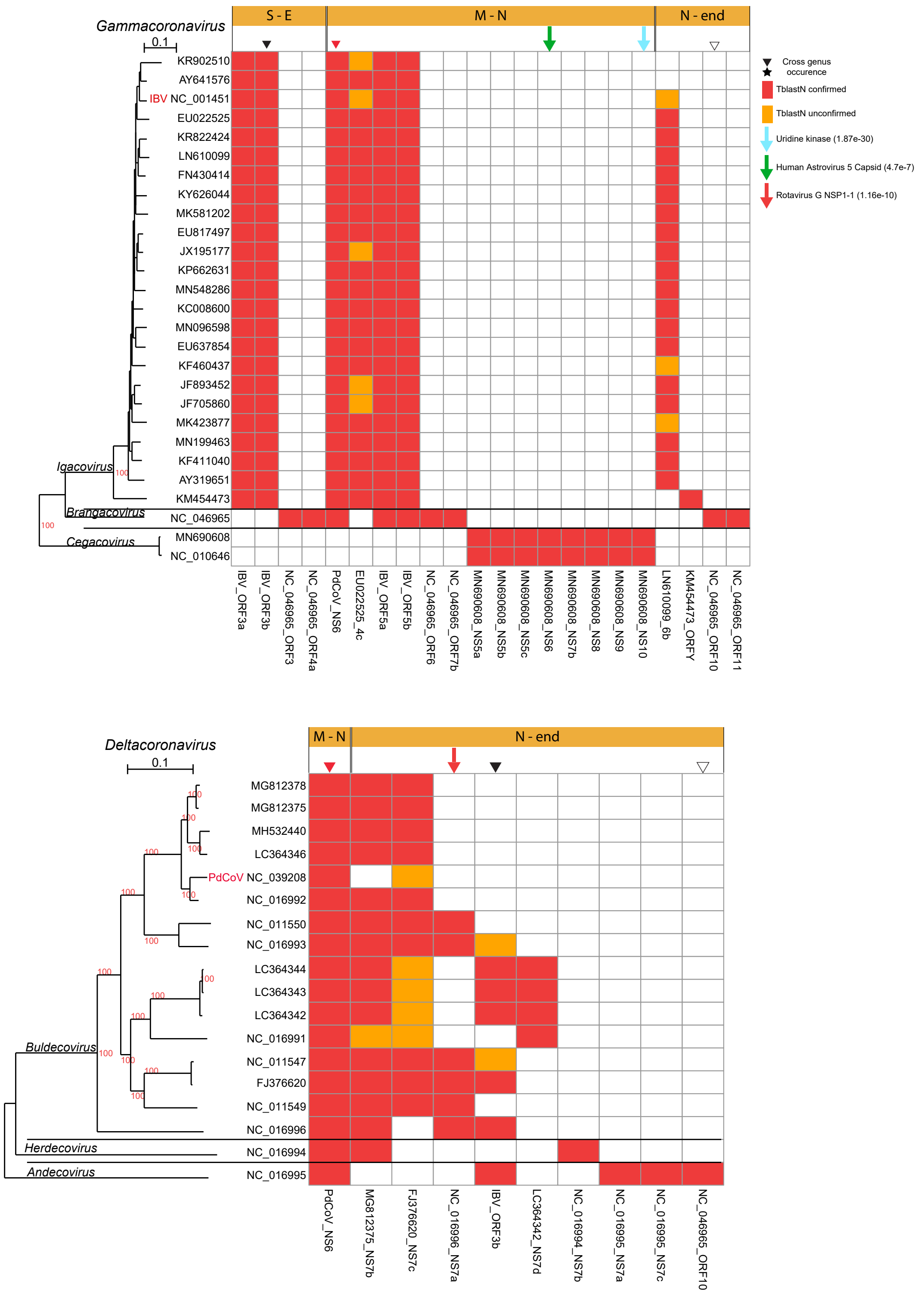

Figure 4. Presence and distribution of Accessory ORF Families (AOFs) in the γ- and δ-CoVs. Each column in the matrix represents a certain AOF. Red color (within the matrix cells) denotes the (TblastN) presence of an AOFs that is also verified by a predicted ORF with length ≥30 aa, whereas if the length of the predicted ORF is $<30$ aa, then it is denoted with orange color. Inverted triangles denote AOFs that are present in both γ- and δ-CoV members. Downward arrows denote AOFs that have homologs in non-CoV genomes, together with their best PSI-Blast hit e-value. Horizontal orange bars (above the matrices) denote the genomic region where the AOF is located, i.e. M-N denotes the region between the Membrane and Nucleocapsid ORFs.

**Availability of Data:** All necessary data are incorporated into the article and its online supplementary material. Any further data are available on request.

**Contributions:** M.N and G.D.A. analyzed the data; M.N, P.M., Y.V.d.P., S.G.O., G.D.A designed the analyses, wrote and edited the manuscript; G.D.A. supervised M.N.

**Acknowledgements:** M.N. would like to thank the Bodossakis foundation (studentship: BDA-394) and the University of Thessaly (studentship: DEKA-UTH-259) for financial support. We thank Stephane Rombauts for useful discussions concerning bacterial genome assembly artefacts.

**Competing interests:** The authors declare no competing interests.

## Supplementary Material

msab292_Supplementary_Data

