# Supplementary material for: The neighborhood of the Spike gene is a hotspot for modular intertypic homologous and non-homologous recombination in Coronavirus genomes
Source: Mol Biol Evol. 2021 Oct 12:msab292. doi: 10.1093/molbev/msab292 (PMC8549283; doi:10.1093/molbev/msab292)
Supplement: msab292_Supplementary_Data [file msab292_Supplementary_Data.zip › suppl_data/SuppFile1_revised_recombination_events_only_20210731.pdf]

Supplementary figures index (Recombination events only)

Section 1: Phylogenetic trees with all 4 genera together for the spike region................................supp. figs. 1-3

Section 2: Recombination events and Robinson - Foulds matrices for α-, β-, γ- and δ-CoVs.......supp. figs. 4-58
2.1 α-CoVs...supp. figs. 4-33
2.2 β-CoVs...supp. figs. 34-39
2.3 γ -CoVs...supp. figs. 40-51
2.4 δ -CoVs...supp. figs. 52-58

Section 3: Recombination analyses within *Sarbecoviruses*...supp. figs. 59-68
3.1 *Sarbecovirus* Poisson distances *to other β-CoV subgenera*.....................................supp. fig. 59
3.2 *Sarbecovirus* tanglegram analysis and simplot..supp. figs. 60-68

Supplementary file 1 Section 1

BioNJ, PhyML and Bayesian trees of all four genera together for the spike region

BioNJ trees were computed with Poisson model and 500 bootstraps

PhyML trees were computed with LGIG model, 4 gamma categories, aLRT and SPR

Bayesian trees were computed with the BEAST software
using the LGIG model and 1 million MCMC simulations

Stars indicate the CoV subgenera that are implicated in recombination events throughout the analyses

Recombinant sequences in CONSEL analyses are colored yellow

The trees in Section 1 show that in the spike region *Deltacoronaviruses* cluster with A1 and A2 *Alphacoronaviruses* (Event 21). Also, *Rhinacovirus* A1, *Luchacovirus* A3, *Sunacovirus* A4 and *Soracovirus* A5 form a distinct monophyletic clade outside of *Alphacoronaviruses* (Events 5, 8-10).

Supp. fig. 1: All genera ORF1ab and Spike trees (BioNJ, PhyML & Bayesian)

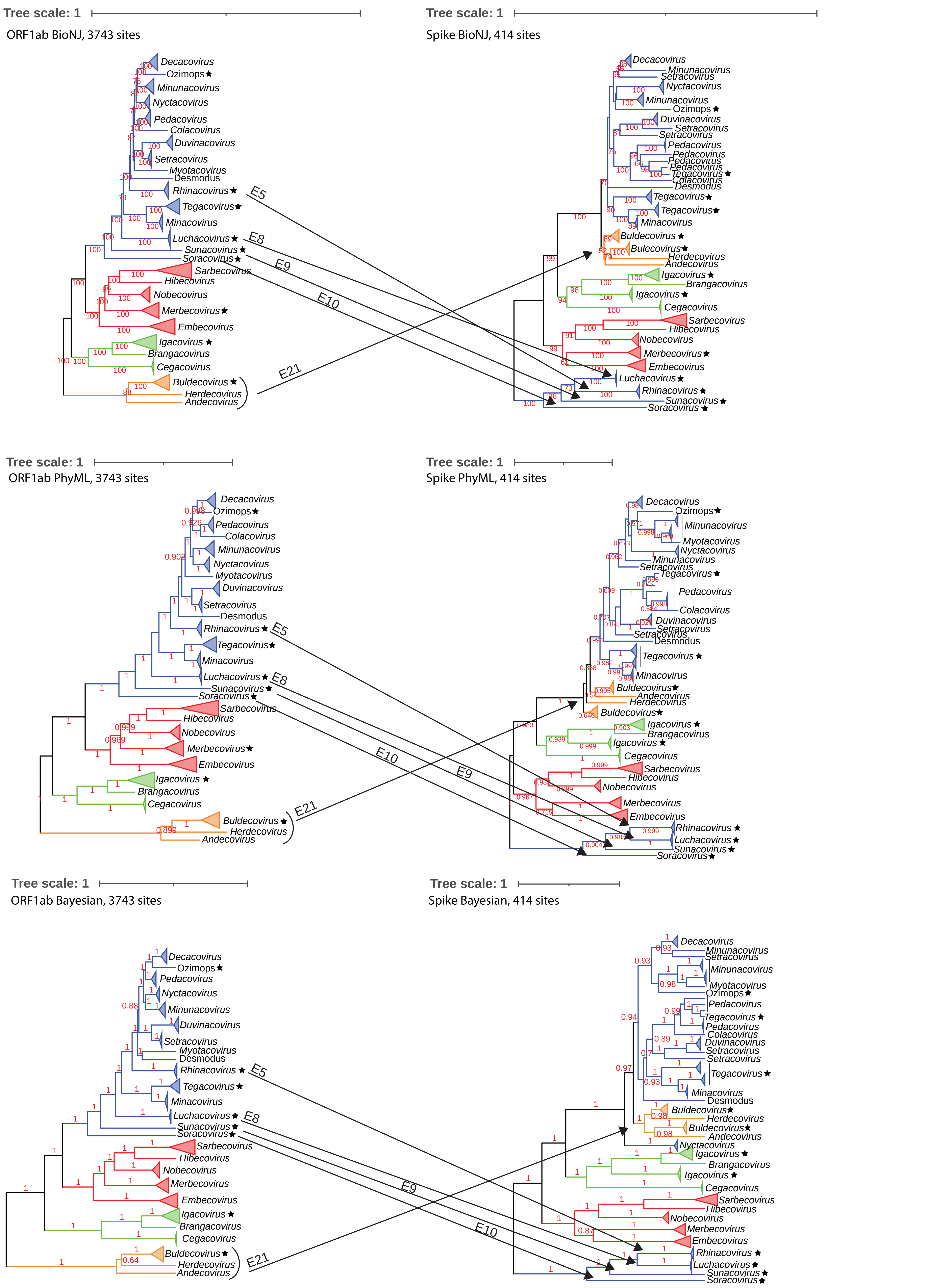

Supp. fig. 2: Events 5, 8-10 CONSEL (ORF1ab LGIG - Spike VTIG PhyML, aLRT, SPR)

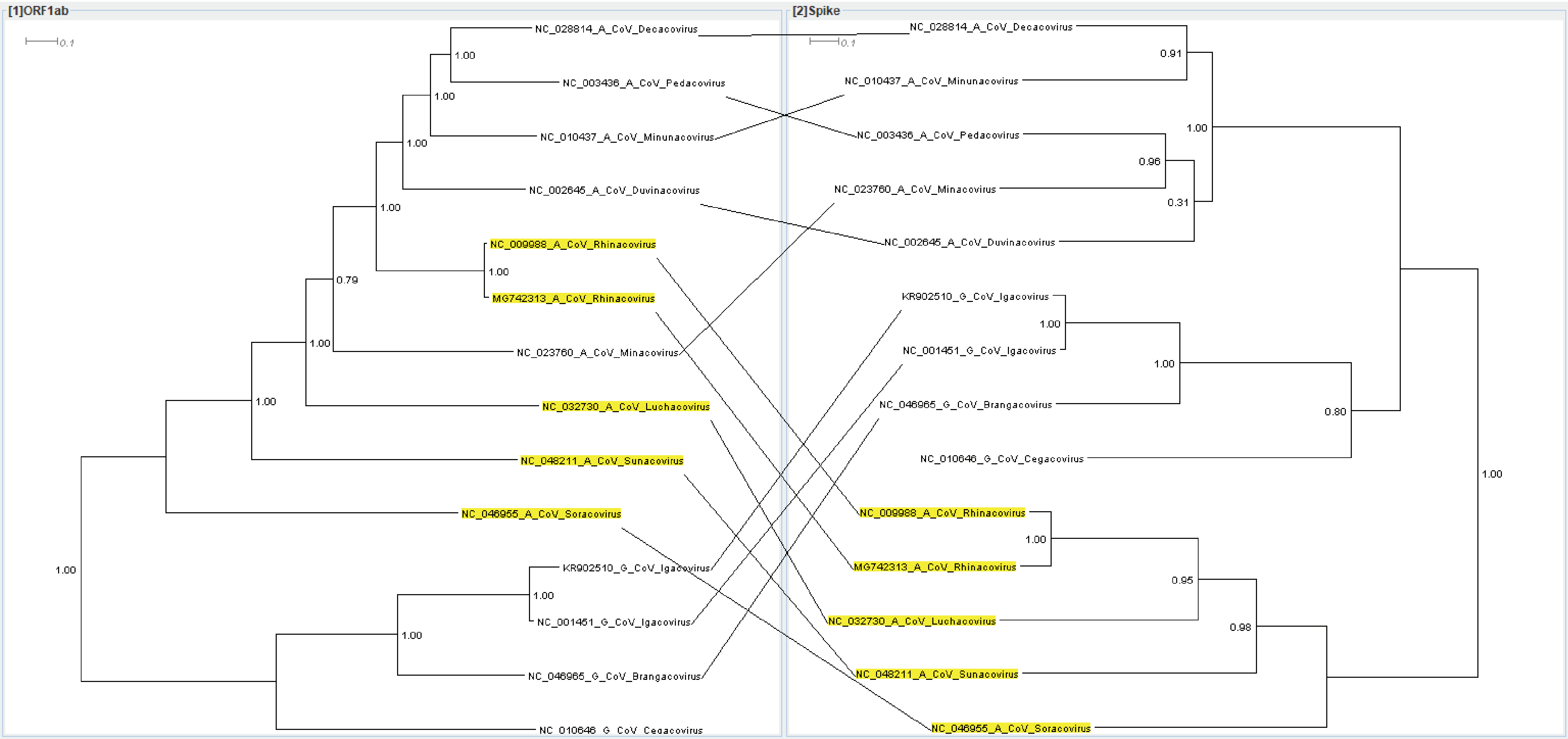

Tree	obs	au	np	bp	pp	kh	sh	wkh	wsh
Spike	-1827.1	1	1	1	1	1	1	1	1
ORF1ab	1827.1	6.00E-45	9.00E-19	0	0	0	0	0	0

Recombinant organisms in CONSEL analyses are colored yellow

Supp. fig. 3: Event 21 CONSEL (ORF1ab LGIG - Spike VTIG PhyML, aLRT, SPR)

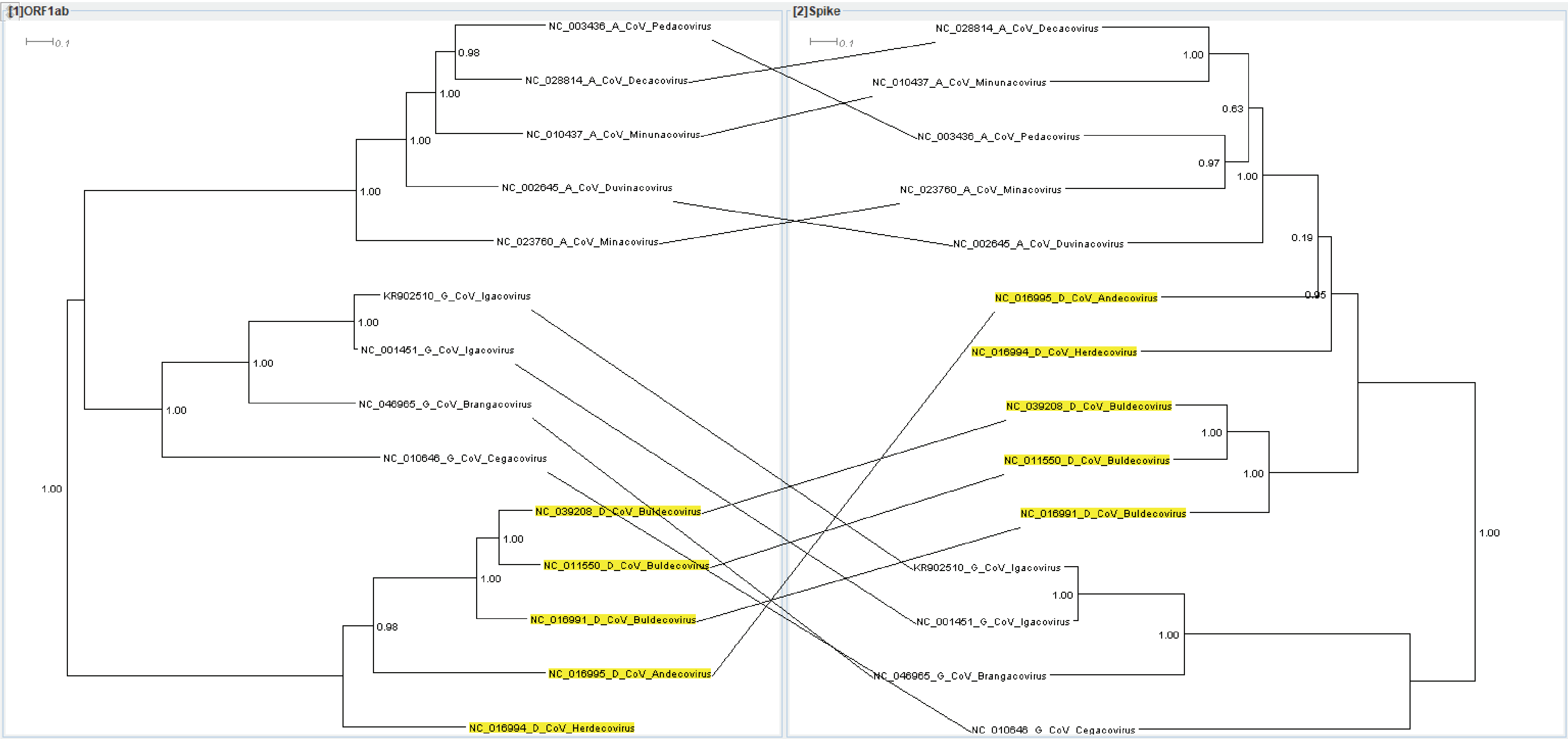

Tree	obs	au	np	bp	pp	kh	sh	wkh	wsh
Spike	-772.6	1	1	1	1	1	1	1	1
ORF1ab	772.6	4.00E-07	1.00E-07	0	0	0	0	0	0

Recombinant organisms in CONSEL analyses are colored yellow

Supplementary file 1 Section 2

Recombination events and Robinson - Foulds matrices for α-, β-, γ- and δ-CoVs

Recombination events were infered using BioNJ, PhyML and Bayesian trees

BioNJ trees were computed with Poisson model and 500 bootstraps
PhyML trees were computed with the best fit model, 4 gamma categories, aLRT and SPR

Bayesian trees were computed with the BEAST software
using the best fit model and 1 million MCMC simulations

Further analyses were conducted with CONSEL, Similarity plots and Bootscan
(when applicable)

Recombinant organisms in CONSEL analyses are colored yellow

Supplementary file 1 section 2, subsection 1

Recombination events for α-CoVs

For easier inspection of the recombination events several α-CoV subgenera have been colored as follows

- *Decacovirus* subgenus
- Ozimops sp.
- *Minunacovirus* subgenus
- *Nyctacovirus* subgenus
- *Colacovirus* subgenus
- *Setracovirus* subgenus
- *Rhinacovirus* subgenus
- *Tegacovirus* subgenus
- *Luchacovirus* subgenus
- *Sunacovirus* subgenus

Phylogenetic incogruences are noted with arrows

Recombinant organisms in CONSEL analyses are colored yellow

- Event 1: *Luchacovirus* A3 nsp1
  This peptide is found within the A1 group, with bootstrap 87, aLRT 0.89, posterior probability 0.93. This event is also supported by CONSEL.
- Event 2: *Luchacovirus* A3 nsp7
  This peptide is found within the A1 group, with bootstrap 82, aLRT 0.95, posterior probability 0.95. This event is also supported by CONSEL.
- Event 3: *Rhinacovirus* A1 nsp8
  For this peptide *Rhinacoviruses* are no longer within the A1 group and they are shown as sister group to *Luchacovirus* A3. Bootstrap support 68, aLRT 0.85, posterior probability 0.99, also supported by CONSEL.
- Event 4: Ozimops A1 nsp16
  This Ozimops (MK472070) peptide is no longer sister group to *Decacovirus* subgenus and is now shown as sister group to the *Minunacovirus* subgenus. Bootstrap support 88, aLRT 0.89, posterior probability 1.00, also supported by CONSEL.
- Event 5: *Rhinacovirus* A1 Spike
  These peptides are part of a monophyletic subgroup together with *Luchacovirus* A3, *Sunacovirus* A4, *Soracovirus* A5. This subgroup is very distinct outside of the α-CoVs. Bootstrap support 100, aLRT 1.0, posterior probability 1. This is also supported by CONSEL. This event is also shown on the phylogenetic trees involving all four genera in supp. fig. 1.
- Event 6: *Tegacovirus* A2 Spike Swine Enteric
  Tegacovirus NC_028806 Swine enteric is known to have obtained its spike from PEDV (NC_003436) via recombination. Bootstrap 100, aLRT 1, posterior probability 1. This is also supported by Simplot, bootscan and CONSEL. Original paper PMID: 26689738.
- Event 7: *Tegacovirus* A2 Spike second incident
  Tegacoviruses are no longer a monophyletic subgenus. Five *Tegacovirus* sequences are no longer part of the *Tegacovirus* subgenus and are forming a monophyletic sister group to the A2 *Minacovirus* subgenus. This is supported by Bootstrap 100, aLRT 0.99, posterior probability 1. This is also supported by Simplot, Bootscan and CONSEL
- Event 8: *Luchacovirus* A3 Spike
  These peptides are part of a monophyletic subgroup together with *Rhinacovirus* A1, *Sunacovirus* A4, *Soracovirus* A5. This subgroup is very distinct outside of the α-CoVs. Bootstrap support 100, aLRT 1.0, posterior probability 1. This is also supported by CONSEL. This event is also shown on the phylogenetic trees involving all four genera in supp. fig. 1.
- Event 9: *Sunacovirus* A4 Spike
  This peptide is part of a monophyletic subgroup together with *Rhinacovirus* A1, *Luchacovirus* A3 and *Soracovirus* A5. This subgroup is very distinct outside of the a-CoVs. Bootstrap support 100, aLRT 1.0, posterior probability 1. This is also supported by CONSEL. This event is also shown on the phylogenetic trees involving all four genera in supp. fig. 1.
- Event 10: *Soracovirus* A5 Spike
  This peptide is part of a monophyletic subgroup together with *Rhinacovirus* A1, *Luchacovirus* A3 and *Sunacovirus* A4. This subgroup is very distinct outside of the α-CoVs. Bootstrap support 100, aLRT 1.0, posterior probability 1. This is also supported by CONSEL. This event is also shown on the phylogenetic trees involving all four genera in supp. fig. 1.
- Event 11: *Sunacovirus* A4 Envelope
  Sunacovirus A4 Envelope is within the A1 group. Bootstrap support 79, aLRT 0.82, posterior probability 1. This is also supported by CONSEL.

Supp. fig. 4: *Alphacoronavirus* ORF1ab - nsp1 BioNJ Tanglegram (Event 1)

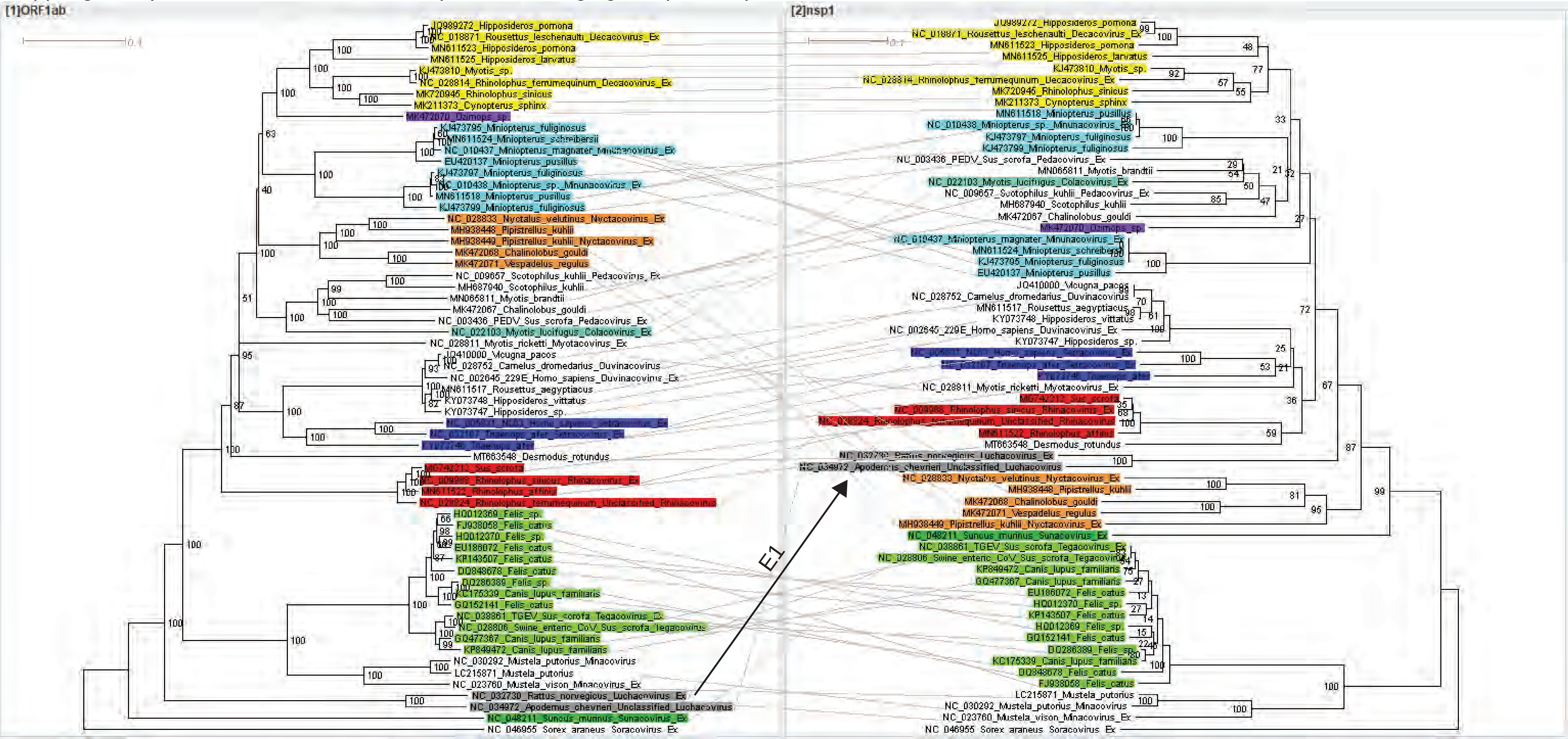

Supp. fig. 5: *Alphacoronavirus* ORF1ab - nsp1 PhyML Tanglegram (Event 1)

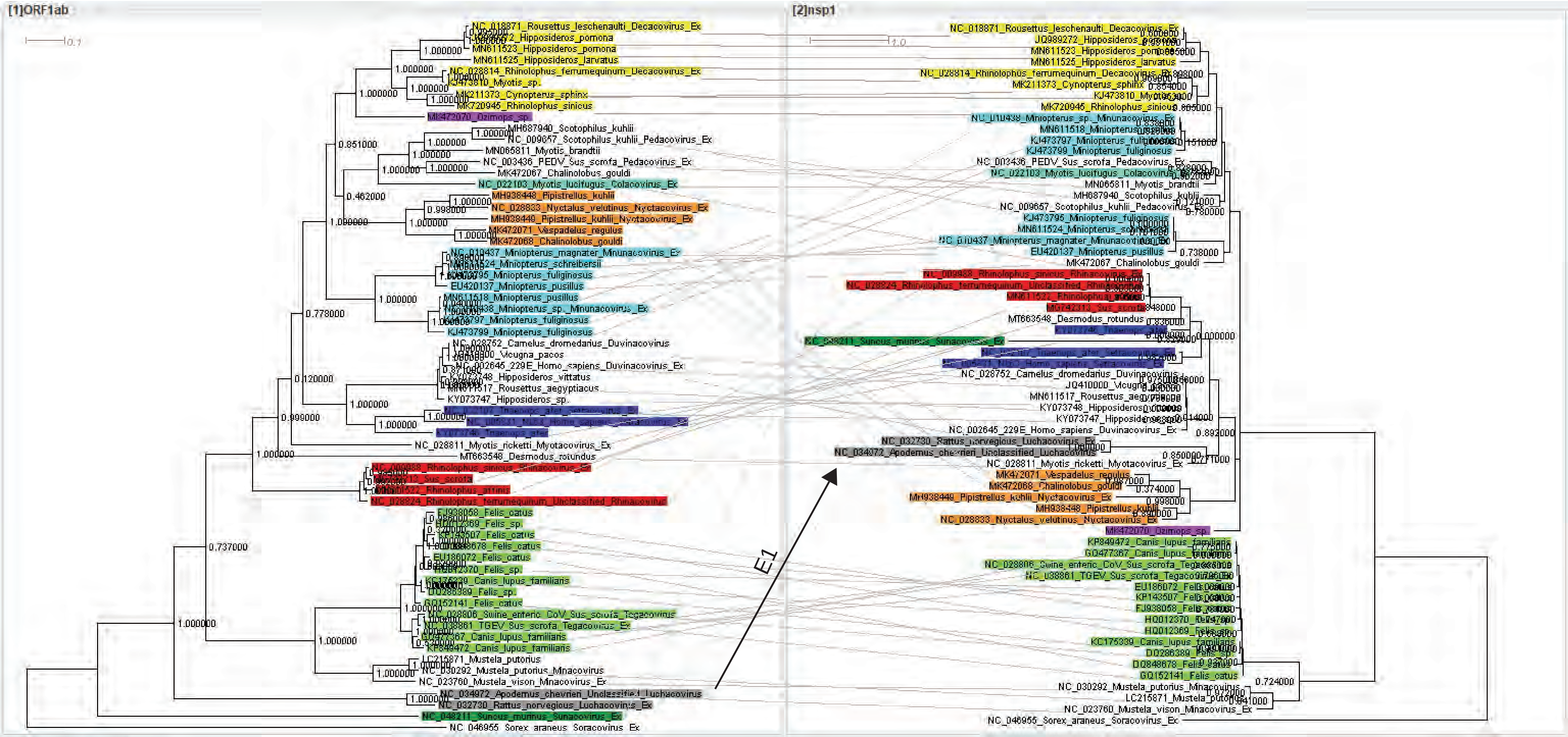

Supp. fig. 6: *Alphacoronavirus* ORF1ab - nsp1 Bayesian Tanglegram (Event 1)

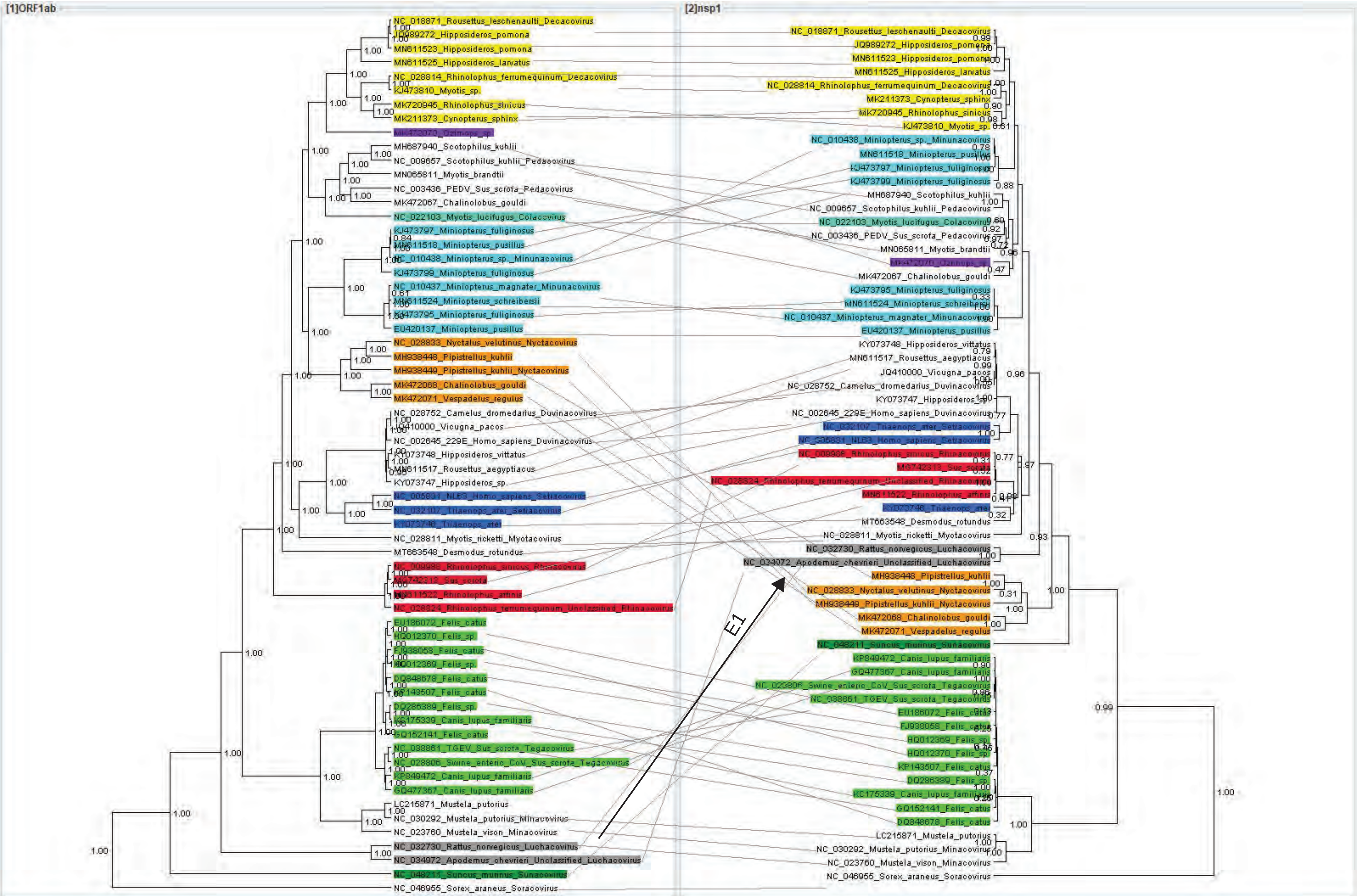

Supp. fig. 7: Event 1 CONSEL (ORF1ab LGIG - nsp1 WAGIG PhyML, aLRT, SPR)

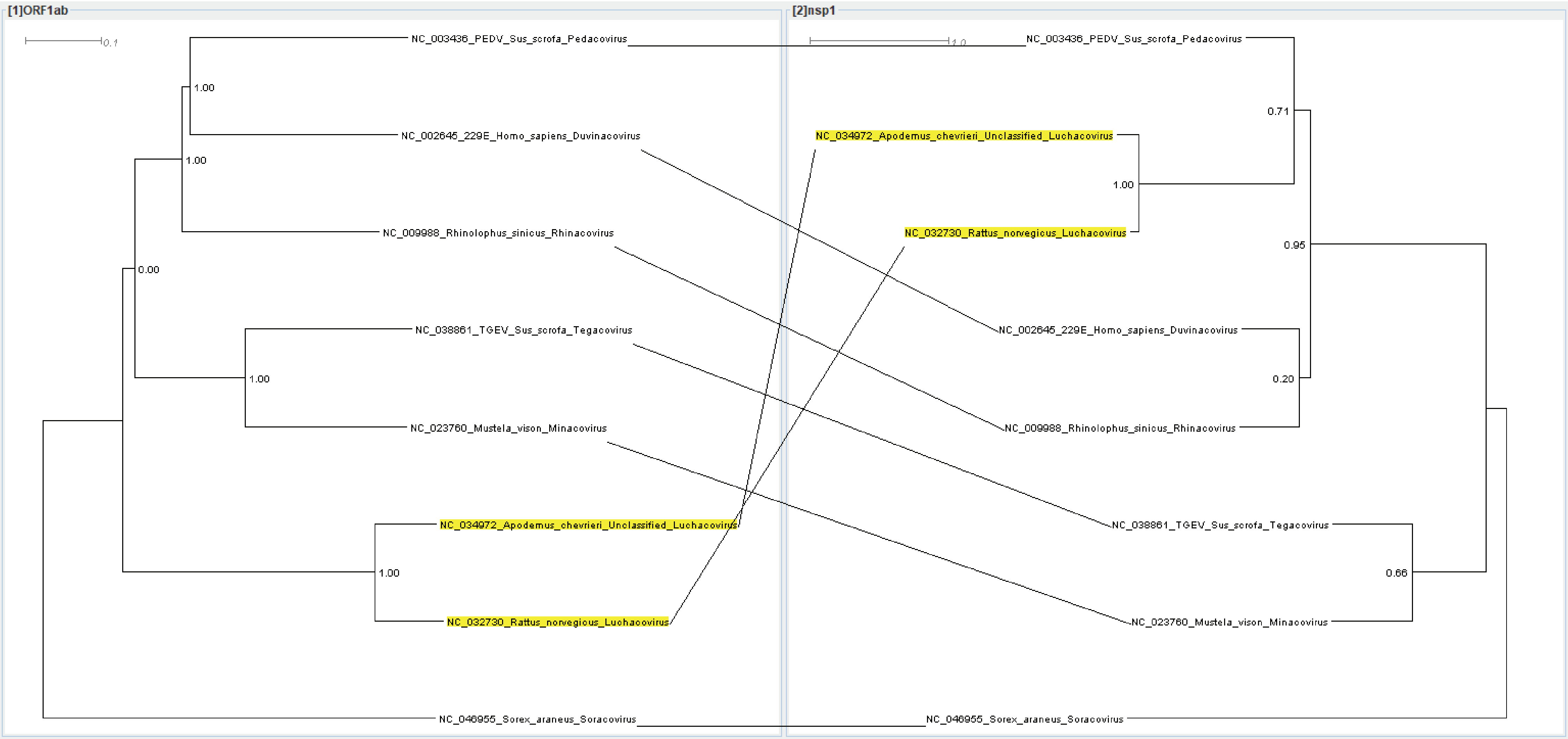

Tree	obs	au	np	bp	pp	kh	sh	wkh	wsh
nsp1	-123.3	1	1	1	1	1	1	1	1
ORF1ab	123.3	3.00E-12	1.00E-08	0	3.00E-54	0	0	0	0

Recombinant organisms in CONSEL analyses are colored yellow

Supp. fig. 8: *Alphacoronavirus* ORF1ab - nsp7 BioNJ Tanglegram (Event 2)

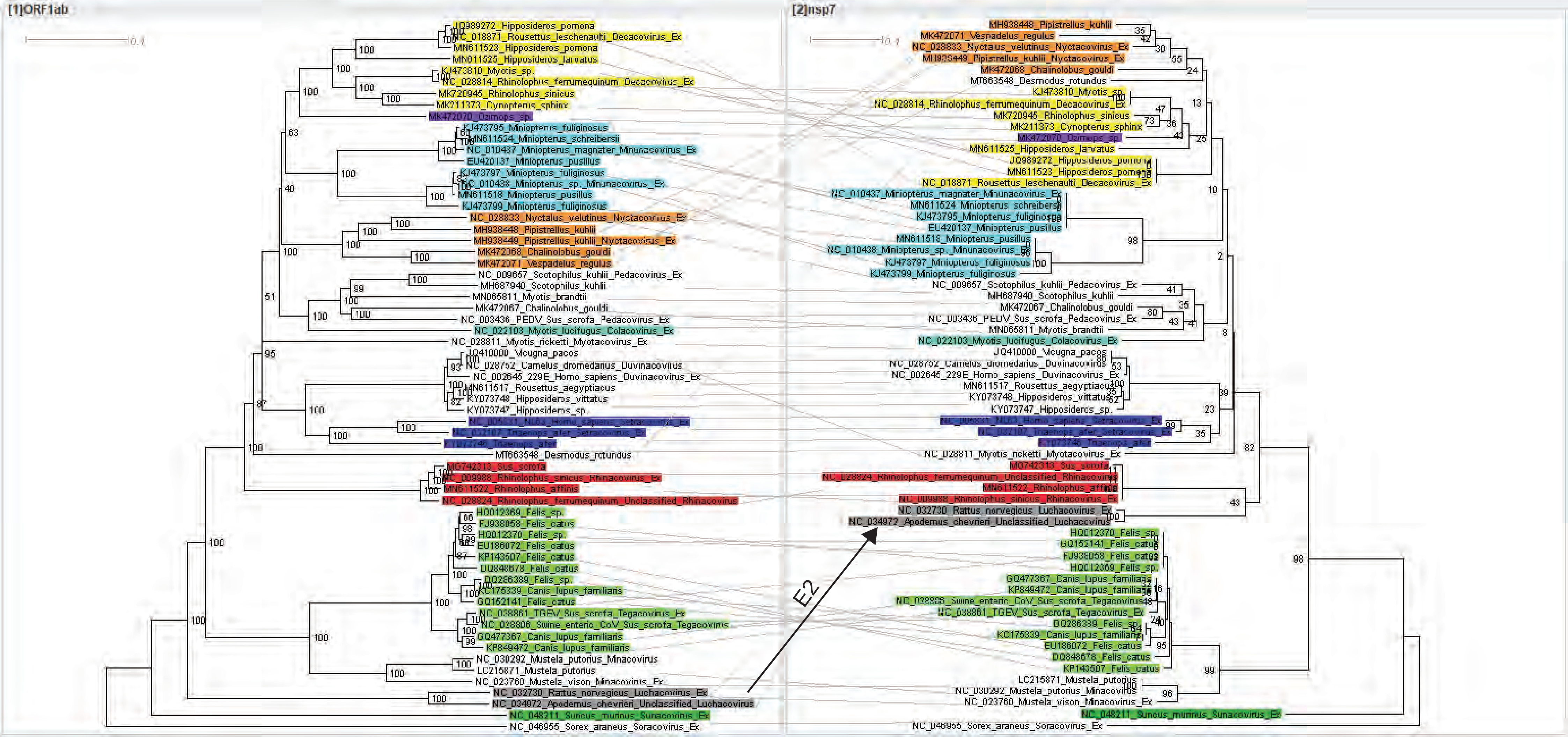

Supp. fig. 9: *Alphacoronavirus* ORF1ab - nsp7 PhyML Tanglegram (Event 2)

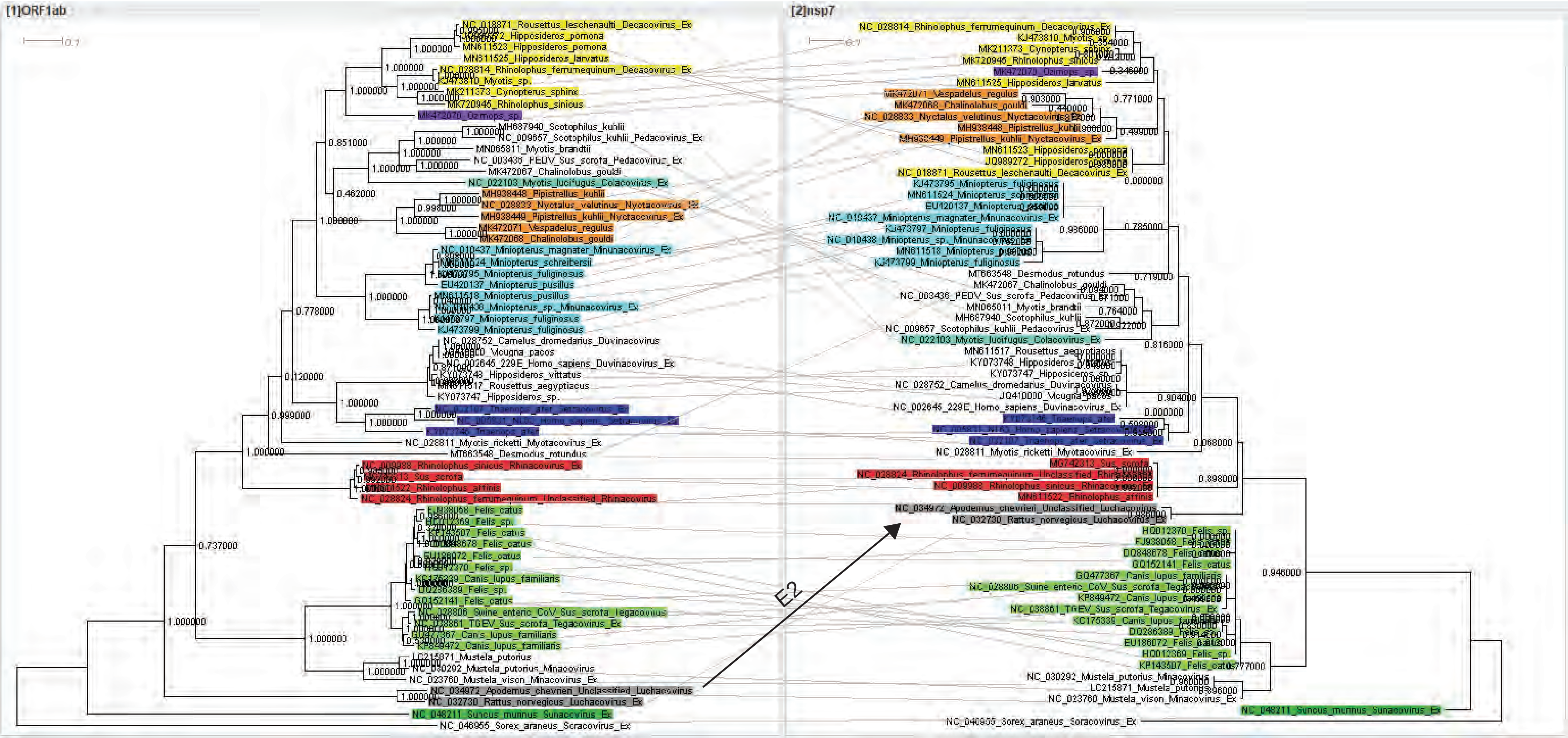

Supp. fig. 10: *Alphacoronavirus* ORF1ab - nsp7 Bayesian Tanglegram (Event 2)

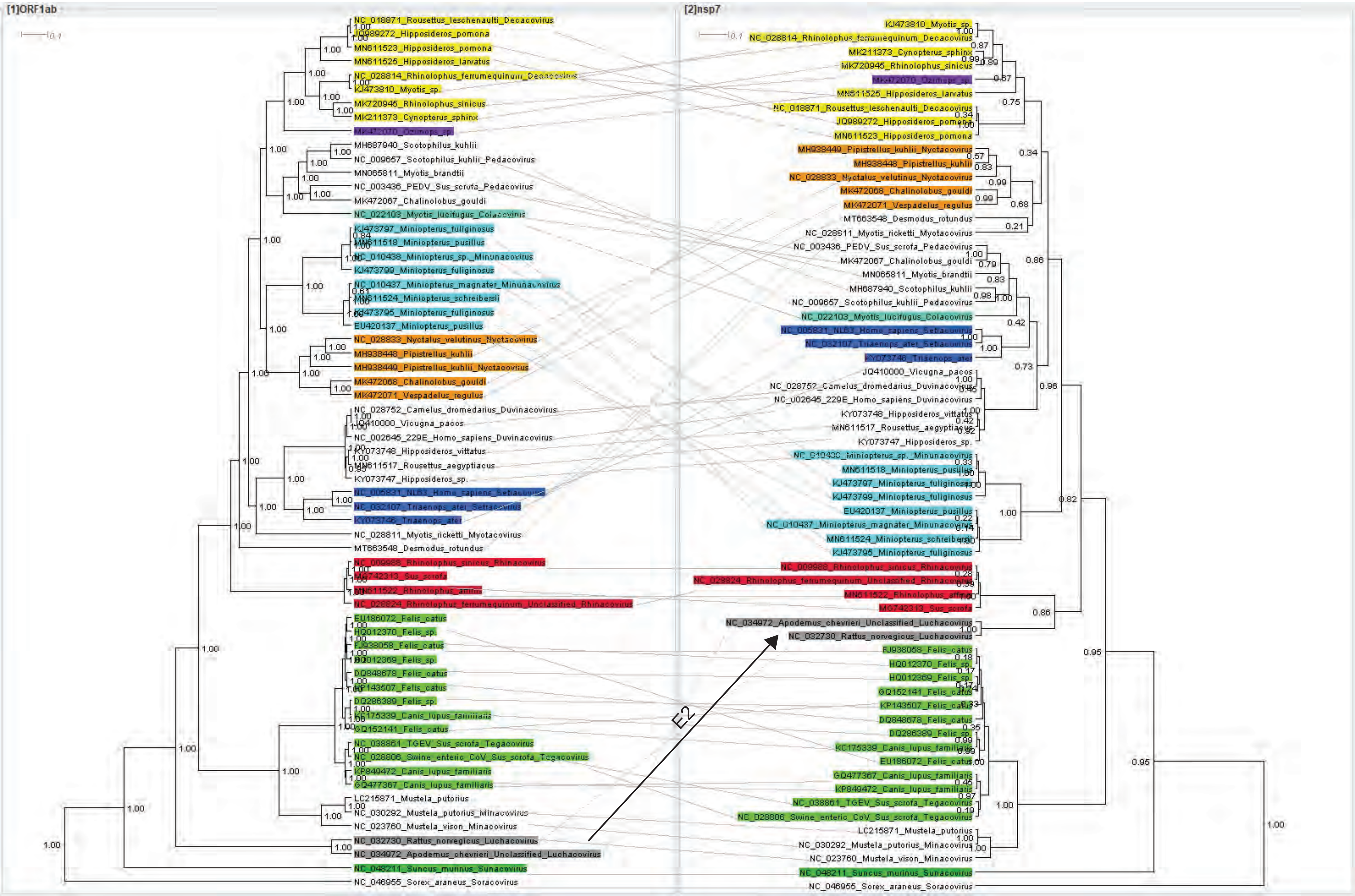

Supp. fig. 11: Event 2 CONSEL (ORF1ab LGIG - nsp7 LGIG PhyML, aLRT, SPR)

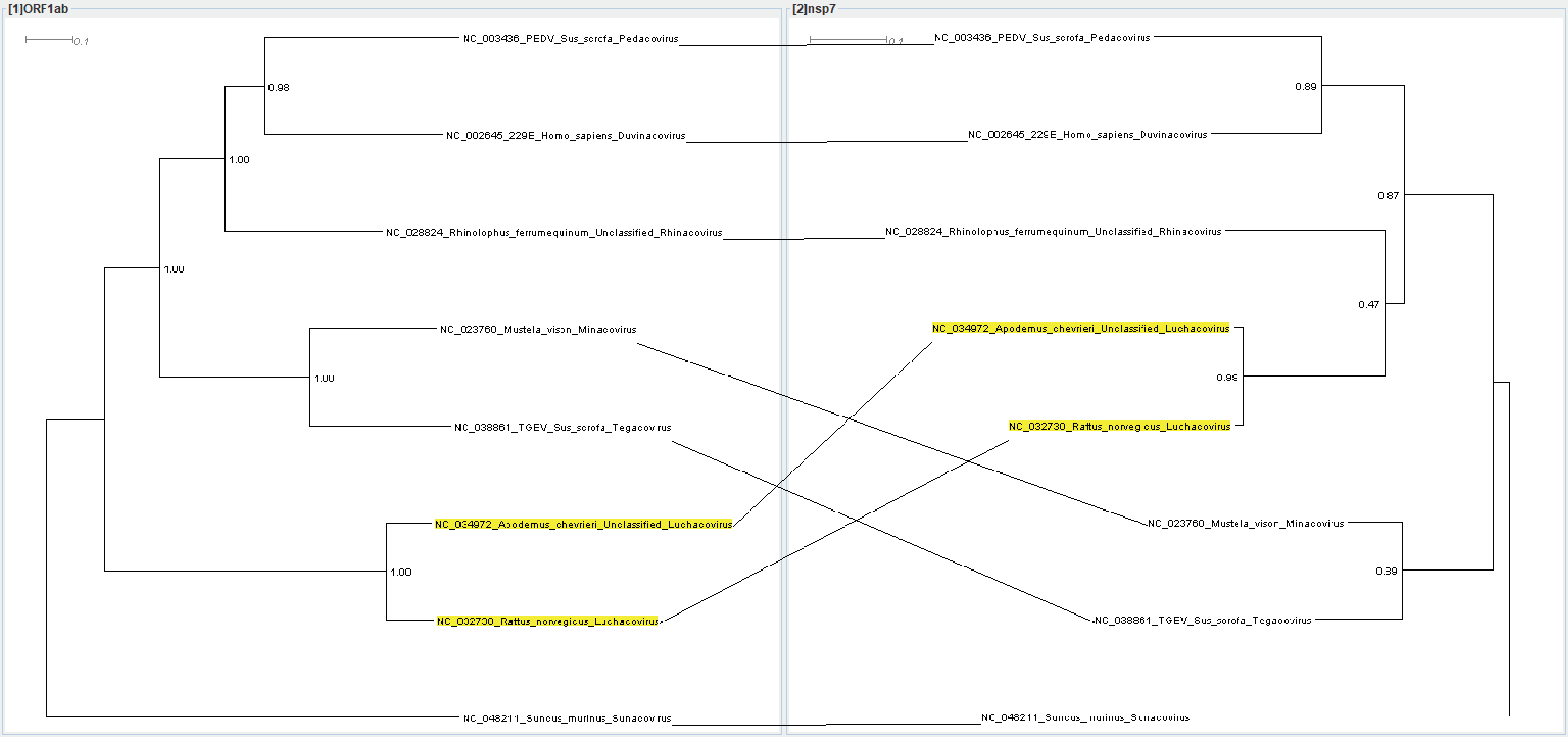

Tree	obs	au	np	bp	pp	kh	sh	wkh	wsh
nsp7	-31.4	1	1	1	1	1	1	1	1
ORF1ab	31.4	6.00E-05	6.00E-05	1.00E-04	2.00E-04	0	0	0	0

Recombinant organisms in CONSEL analyses are colored yellow

Supp. fig. 12: *Alphacoronavirus* ORF1ab - nsp8 BioNJ Tanglegram (Event 3)

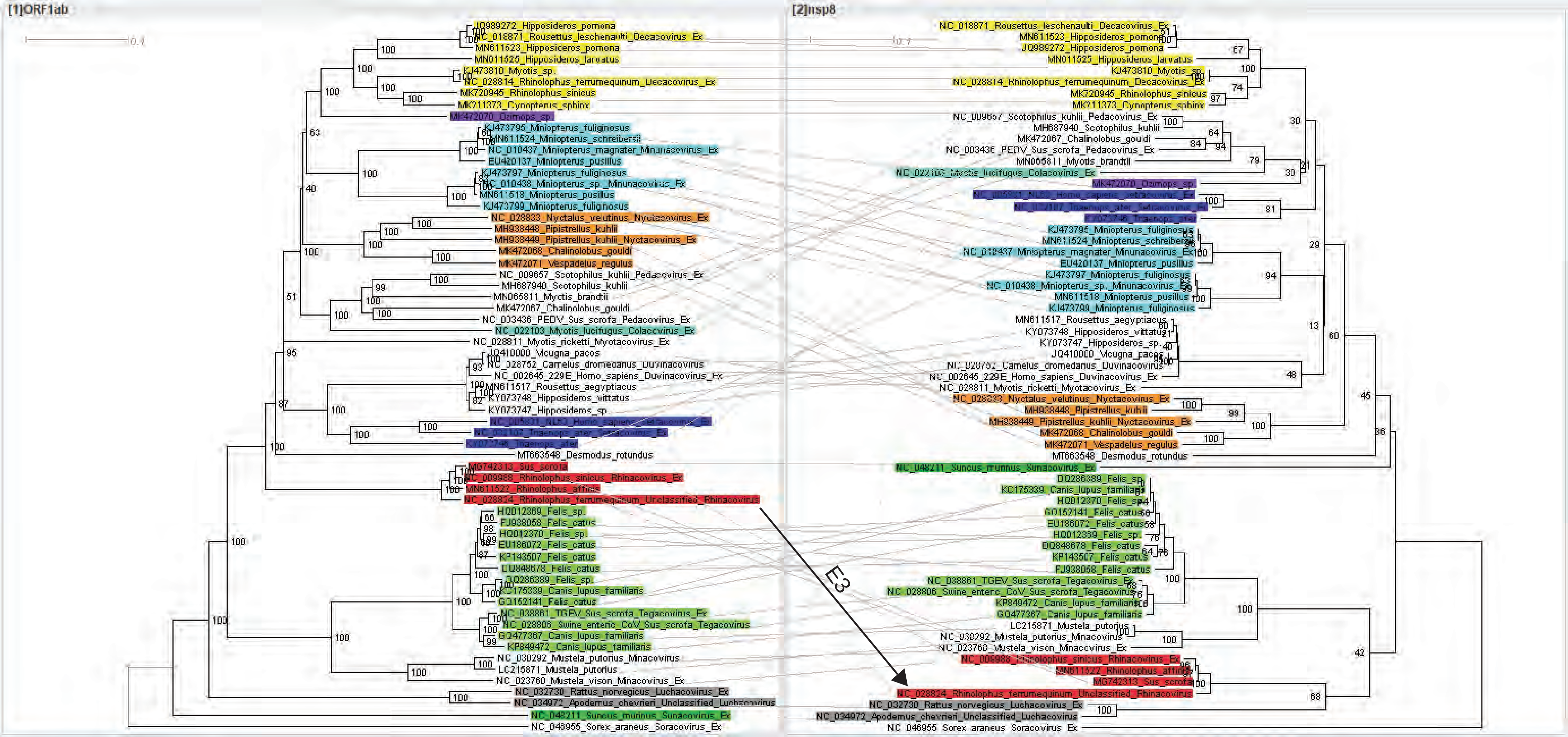

Supp. fig. 13: *Alphacoronavirus* ORF1ab - nsp8 PhyML Tanglegram (Event 3)

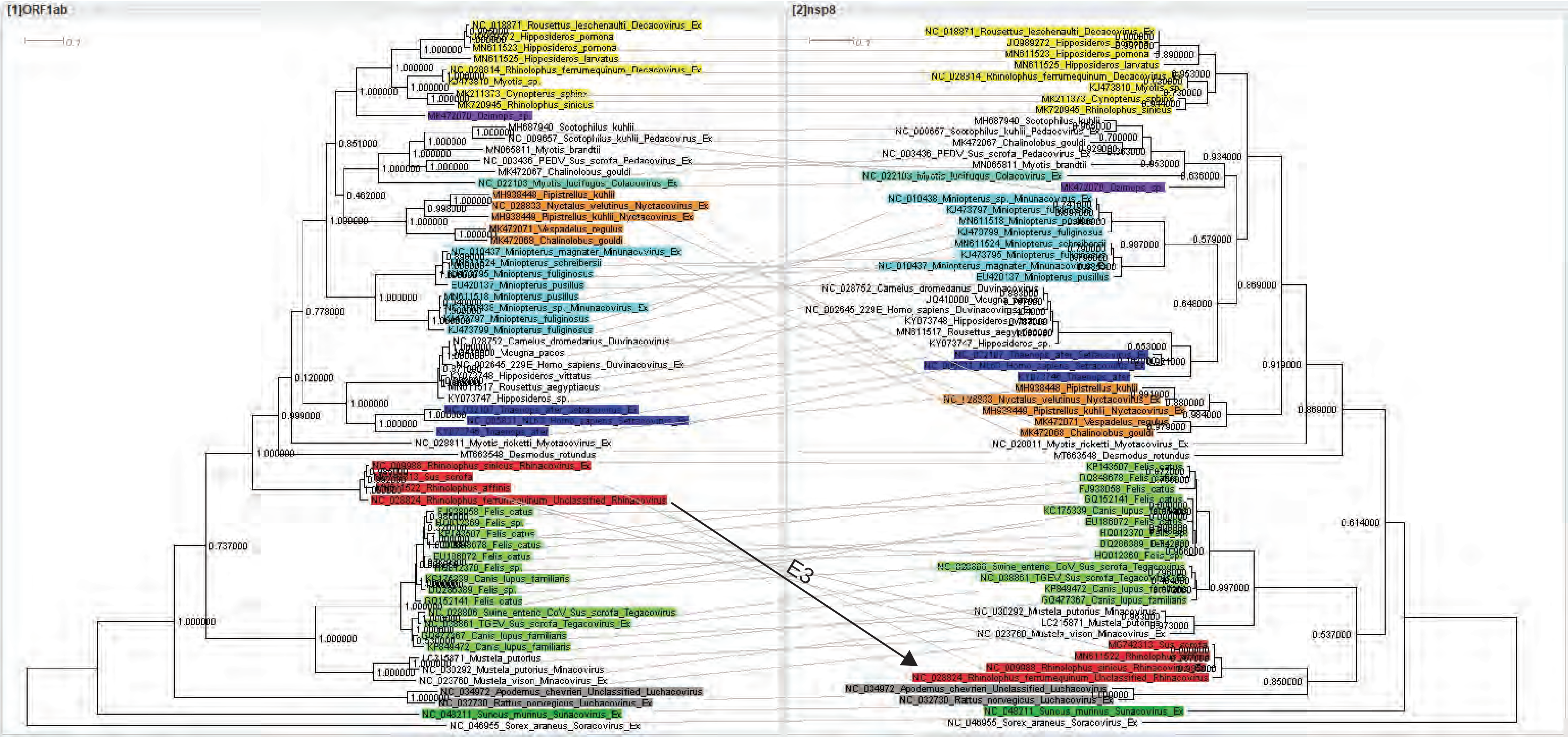

Supp. fig. 14: *Alphacoronavirus* ORF1ab - nsp8 Bayesian Tanglegram (Event 3)

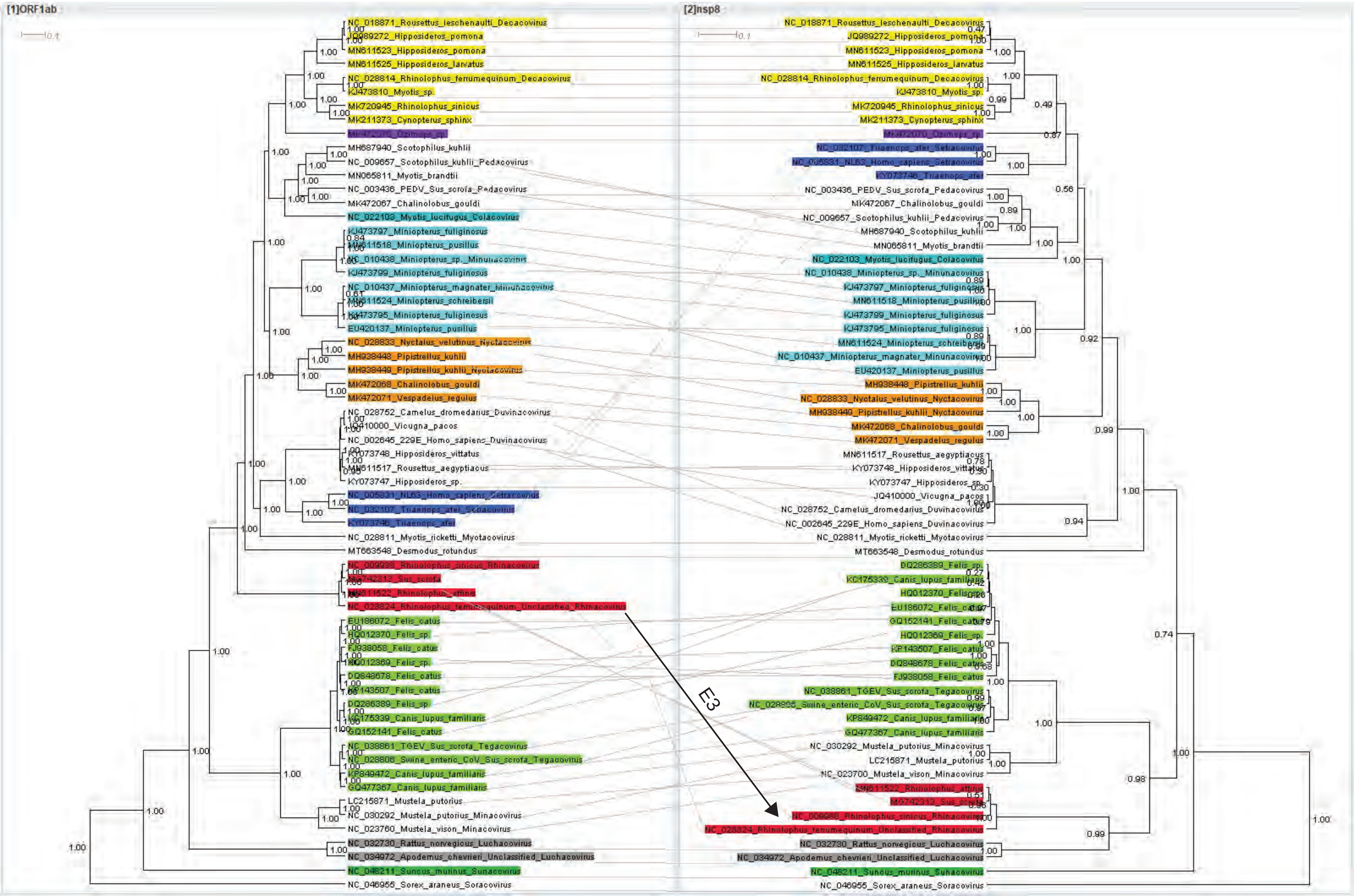

Supp. fig. 15: Event 3 CONSEL (ORF1ab LGIG - nsp8 LGIG PhyML, aLRT, SPR)

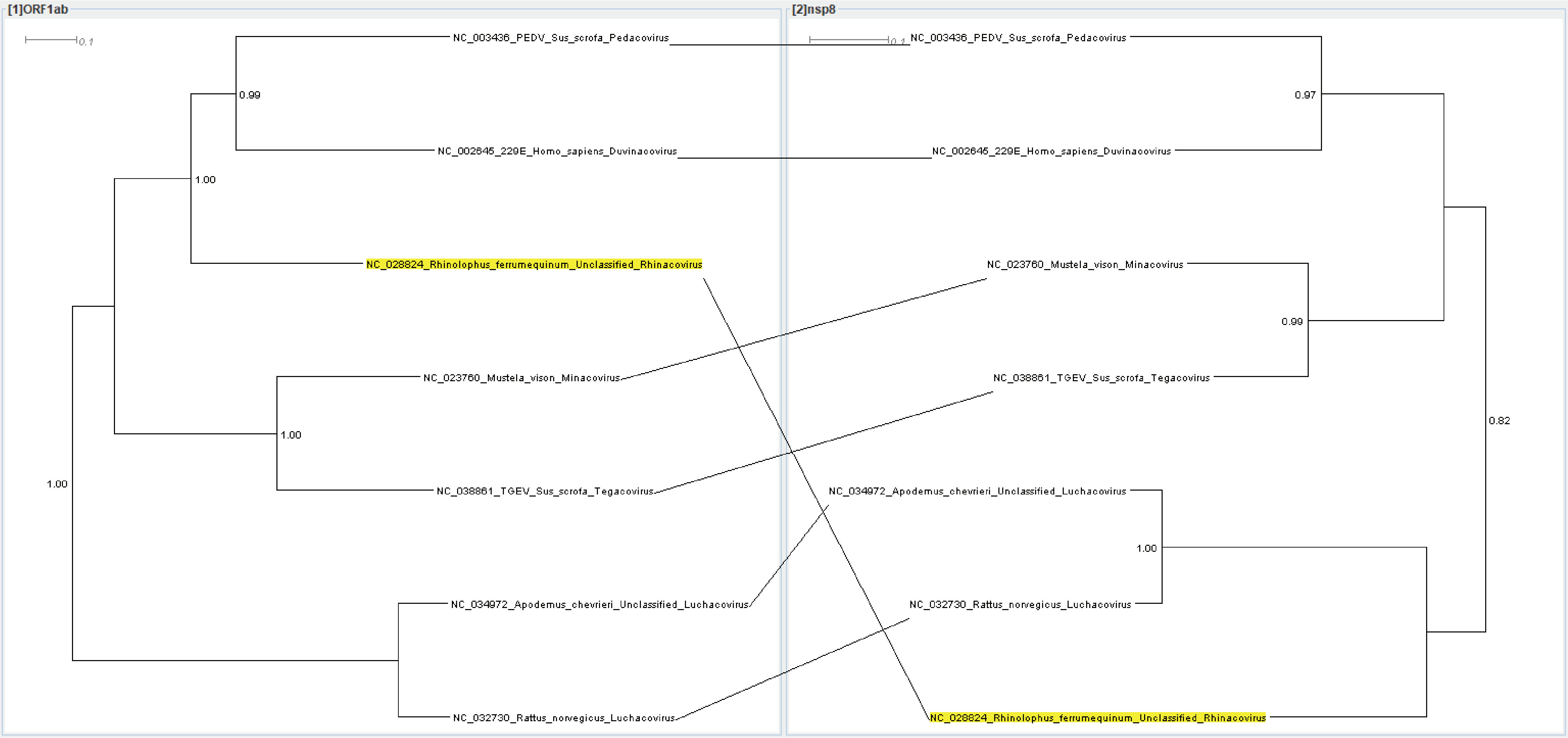

Tree	obs	au	np	bp	pp	kh	sh	wkh	wsh
nsp8	-35.1	1	1	1	1	1	1	1	1
ORF1ab	35.1	3.00E-04	2.00E-04	3.00E-04	6.00E-16	1.00E-04	1.00E-04	1.00E-04	1.00E-04

Recombinant organisms in CONSEL analyses are colored yellow

Supp. fig. 16: *Alphacoronavirus* ORF1ab - nsp16 BioNJ Tanglegram (Event 4)

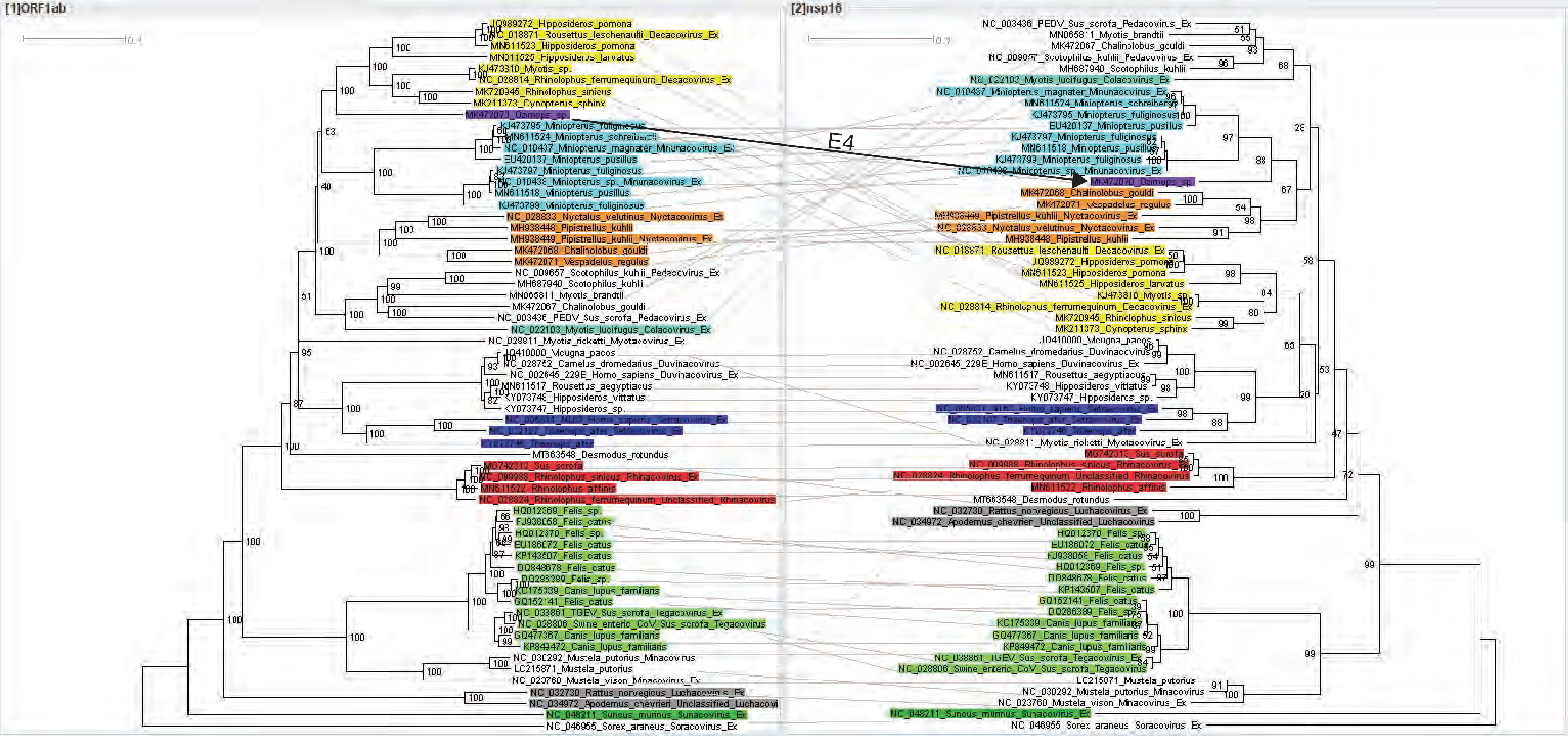

Supp. fig. 17: *Alphacoronavirus* ORF1ab - nsp16 PhyML Tanglegram (Event 4)

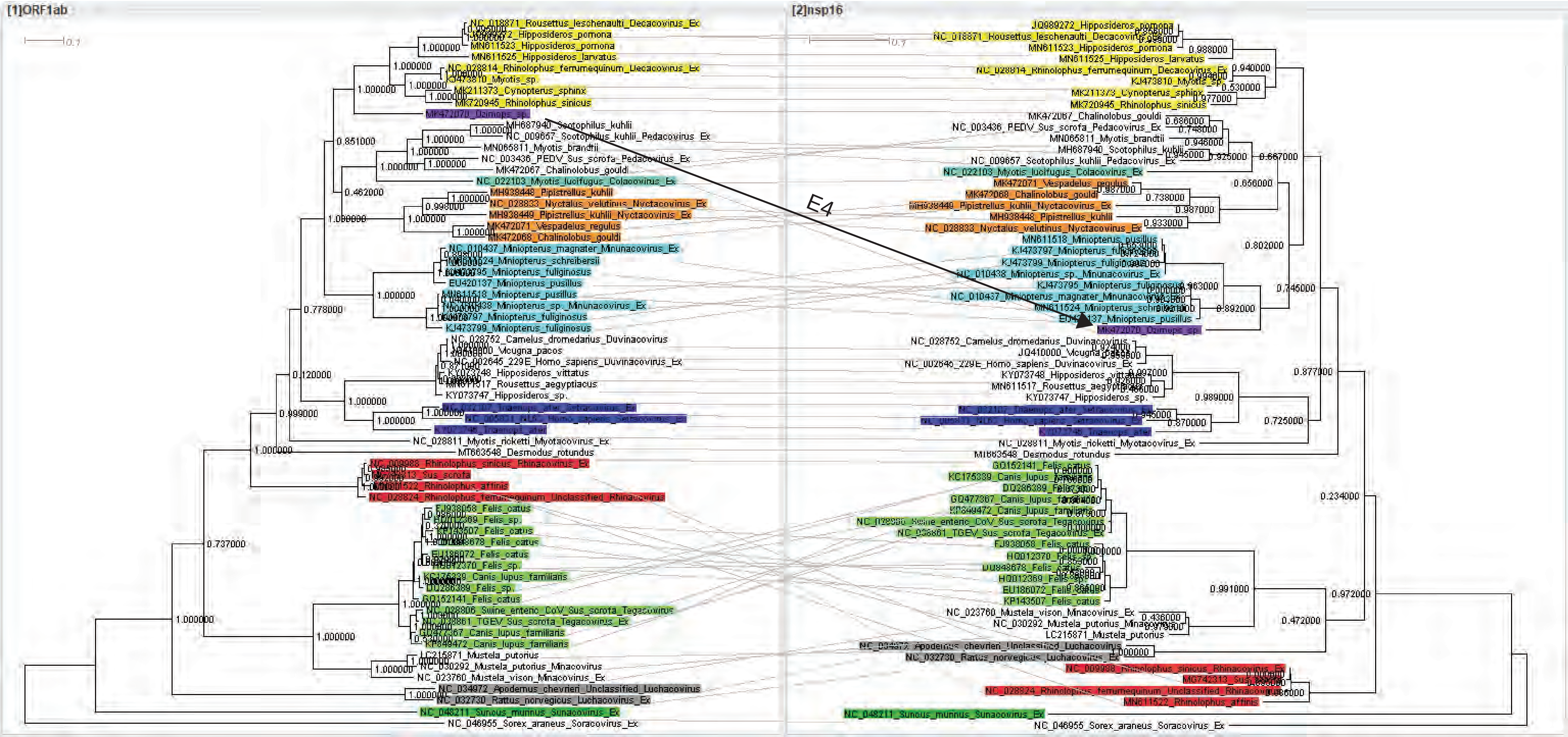

Supp. fig. 18: *Alphacoronavirus* ORF1ab - nsp16 Bayesian Tanglegram (Event 4)

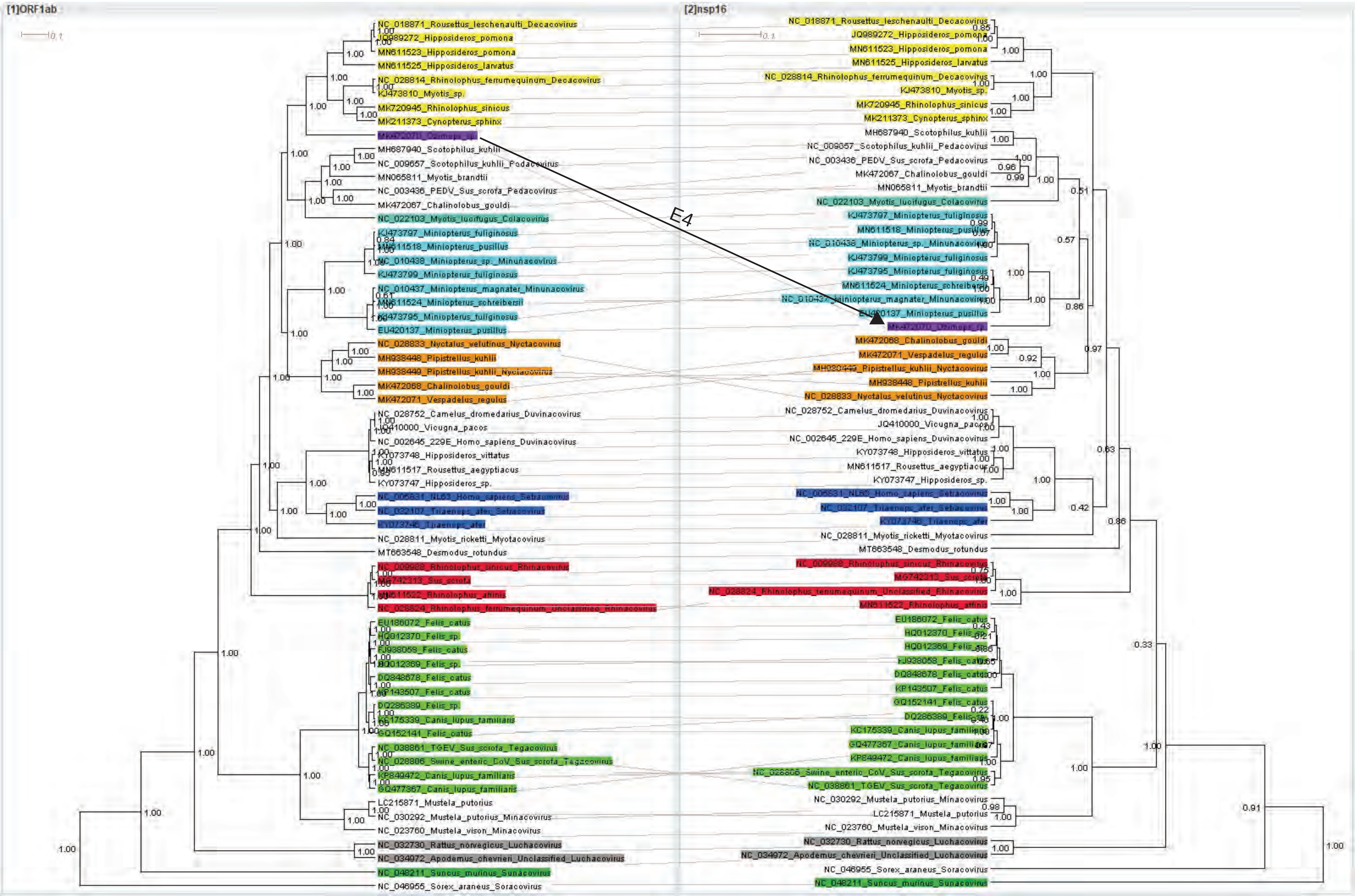

Supp. fig. 19: Event 4 CONSEL (ORF1ab LGIG - nsp16 LGIG PhyML, aLRT, SPR)

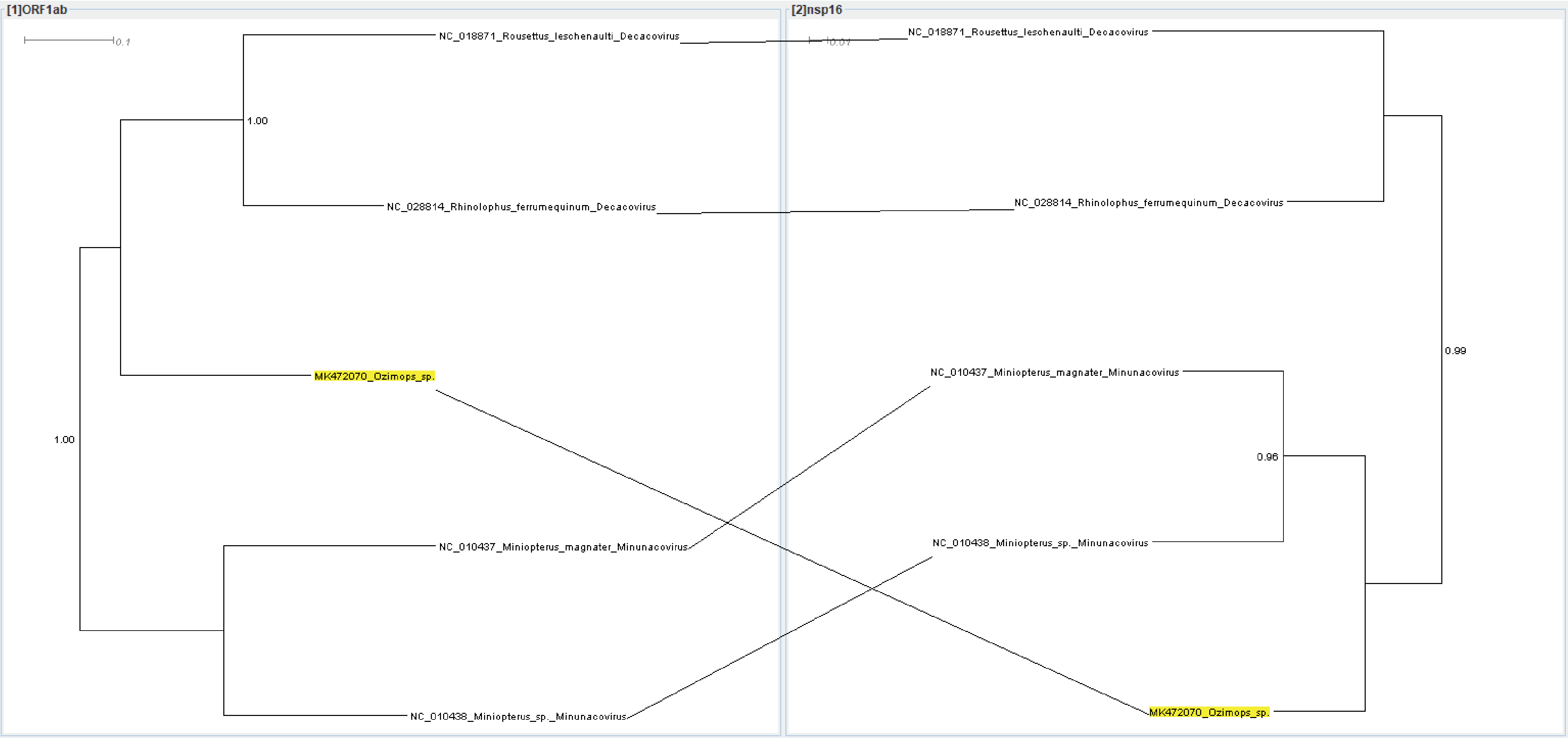

Tree	obs	au	np	bp	pp	kh	sh	wkh	wsh
nsp16	-66.4	1	1	1	1	1	1	1	1
ORF1ab	66.4	1.00E-07	5.00E-07	0	1.00E-29	0	0	0	0

Recombinant organisms in CONSEL analyses are colored yellow

Supp. fig. 20: *Alphacoronavirus* ORF1ab - Spike BioNJ Tanglegram (Events 5-10)

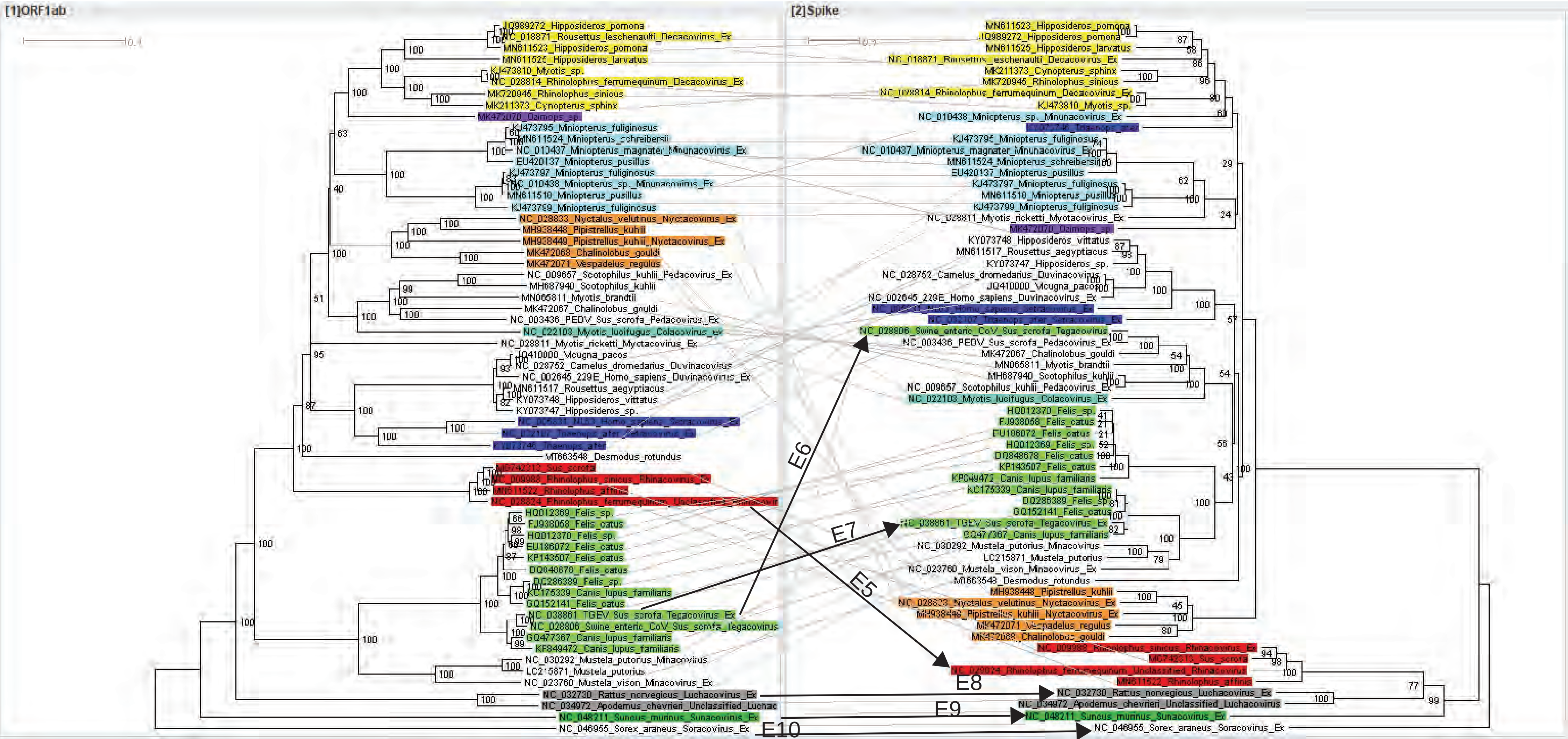

Supp. fig. 21: *Alphacoronavirus* ORF1ab - Spike PhyML Tanglegram (Events 5-10)

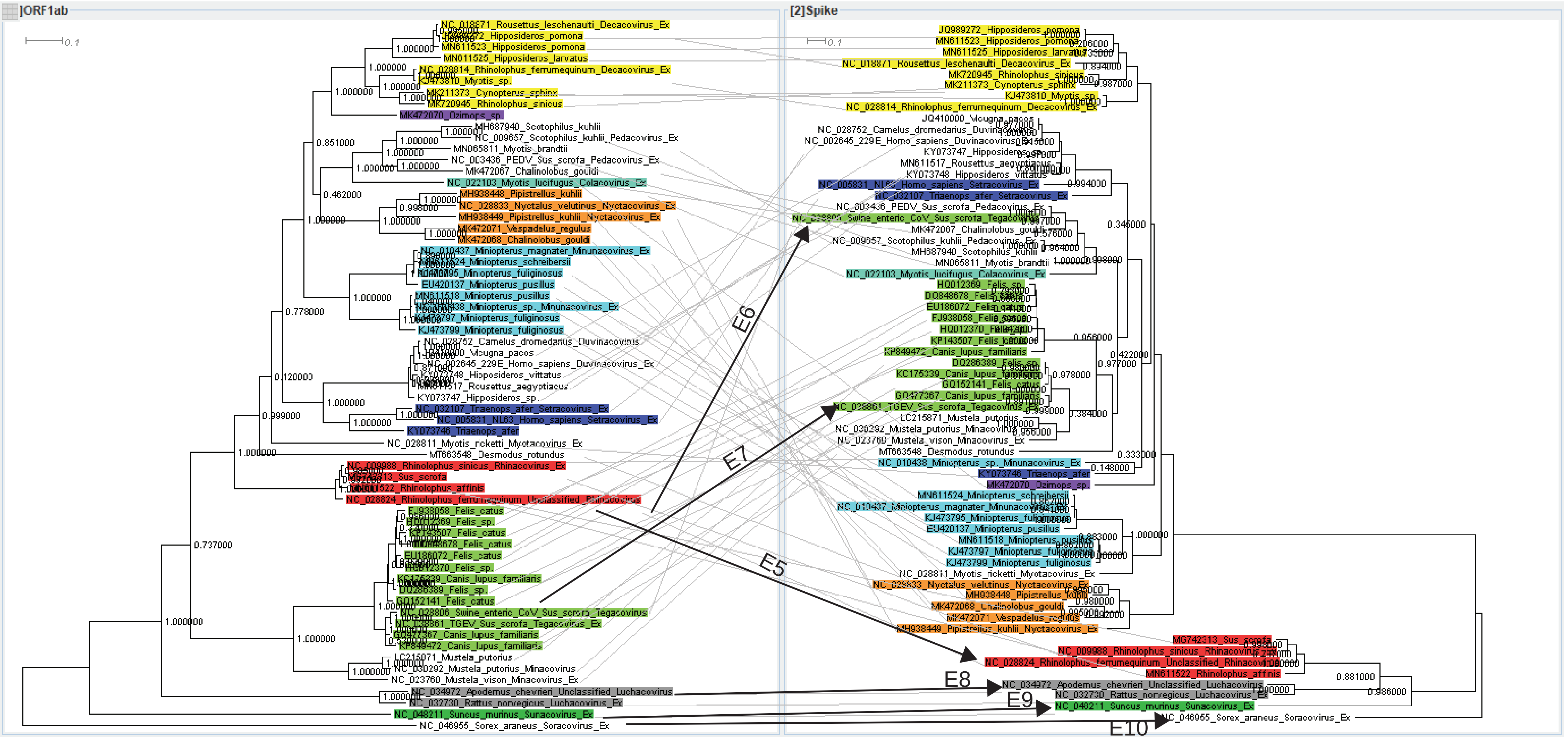

Supp. fig. 22: *Alphacoronavirus* ORF1ab - Spike Bayesian Tanglegram (Events 5-10)

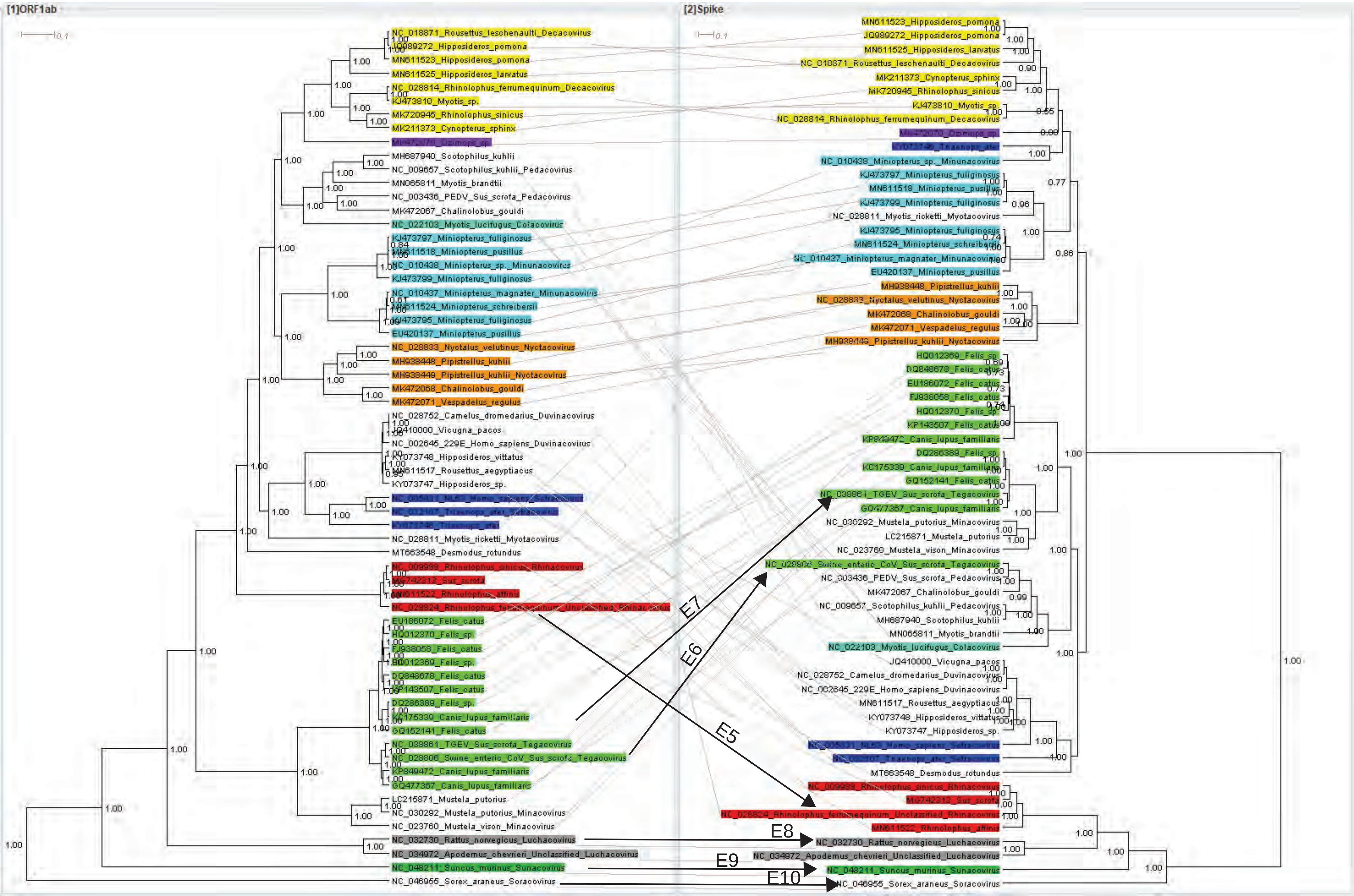

Supp. fig. 23: Event 5 CONSEL (ORF1ab LGIG - Spike WAGIG PhyML, aLRT, SPR)

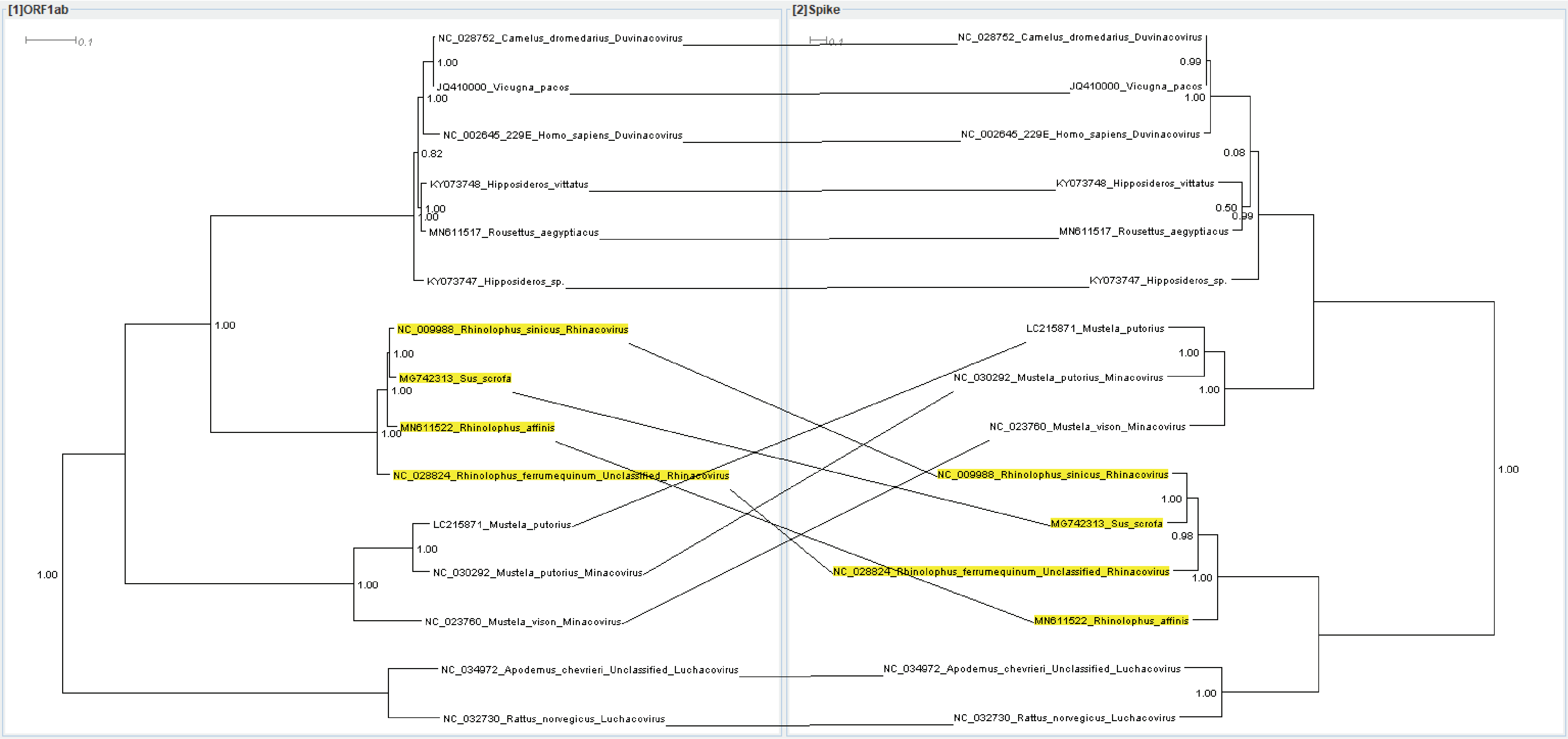

Tree	obs	au	np	bp	pp	kh	sh	wkh	wsh
Spike	-2709.5	1	1	1	1	1	1	1	1
ORF1ab	2709.5	4.00E-65	5.00E-20	0	0	0	0	0	0

Recombinant organisms in CONSEL analyses are colored yellow

Supp. fig. 24: Recombination event 6 Similarity plot and Bootscan analyses

A) *Alphacoronavirus Tegacovirus* (Swine Enteric) Simplot:

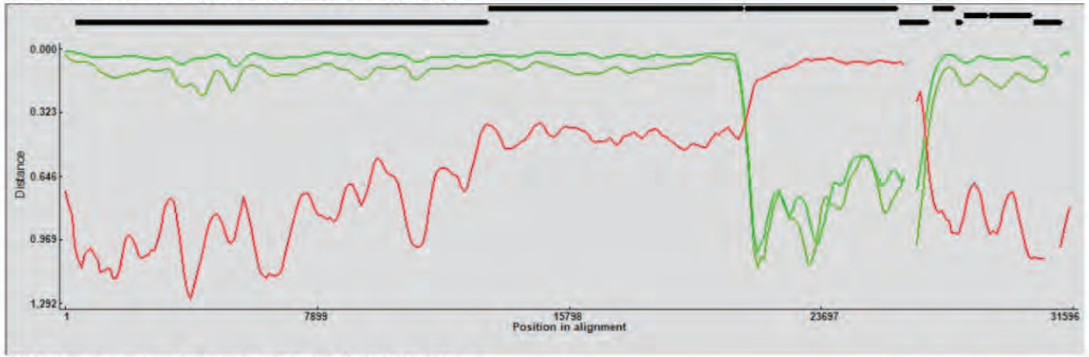

B) *Alphacoronavirus Tegacovirus* (Swine Enteric) Bootscan:

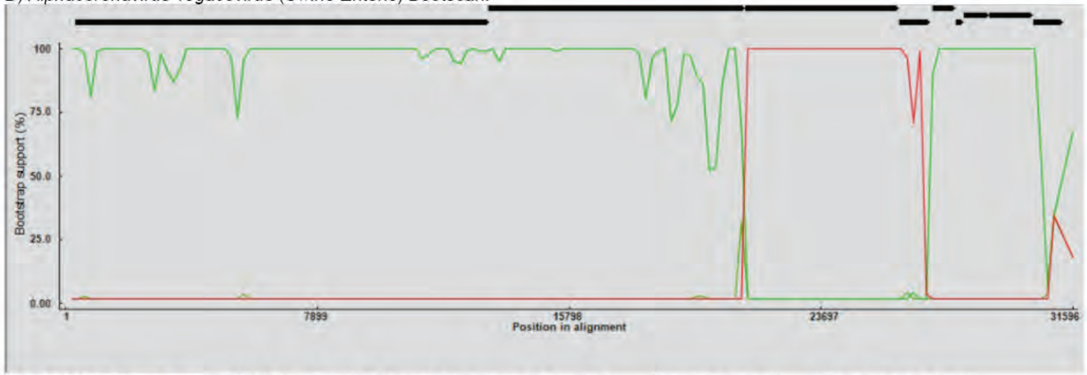

NC_028808 Swine enteric CoV_Sus_Scrofa_Tegacovirus scanned against:
- KP849472_Canis_lupus_familiaris
- NC_038861_TGEV_Sus_scrofa_Tegacovirus_Ex
- NC_003436_PEDV_Sus_scrofa_Pedacovirus_Ex

Simplot params:
Window size:500
Step size:100

Bootscan params:
Window size:500
Step size:200
Bootstrap replicates:100
Pairwise distances

C) *Alphacoronavirus Tegacovirus* ORF1ab vs Spike Tanglegram

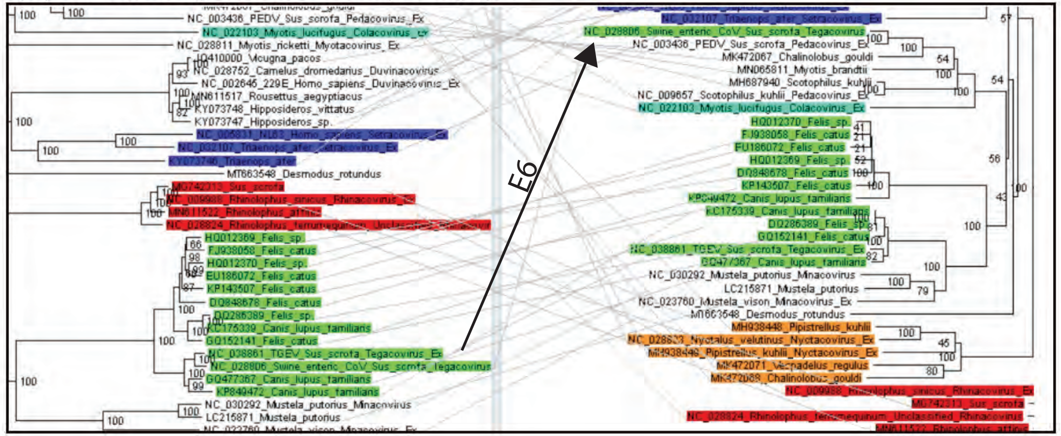

Supp. fig. 25: Event 6 CONSEL (ORF1ab LGIG - Spike WAGIG PhyML , aLRT, SPR))

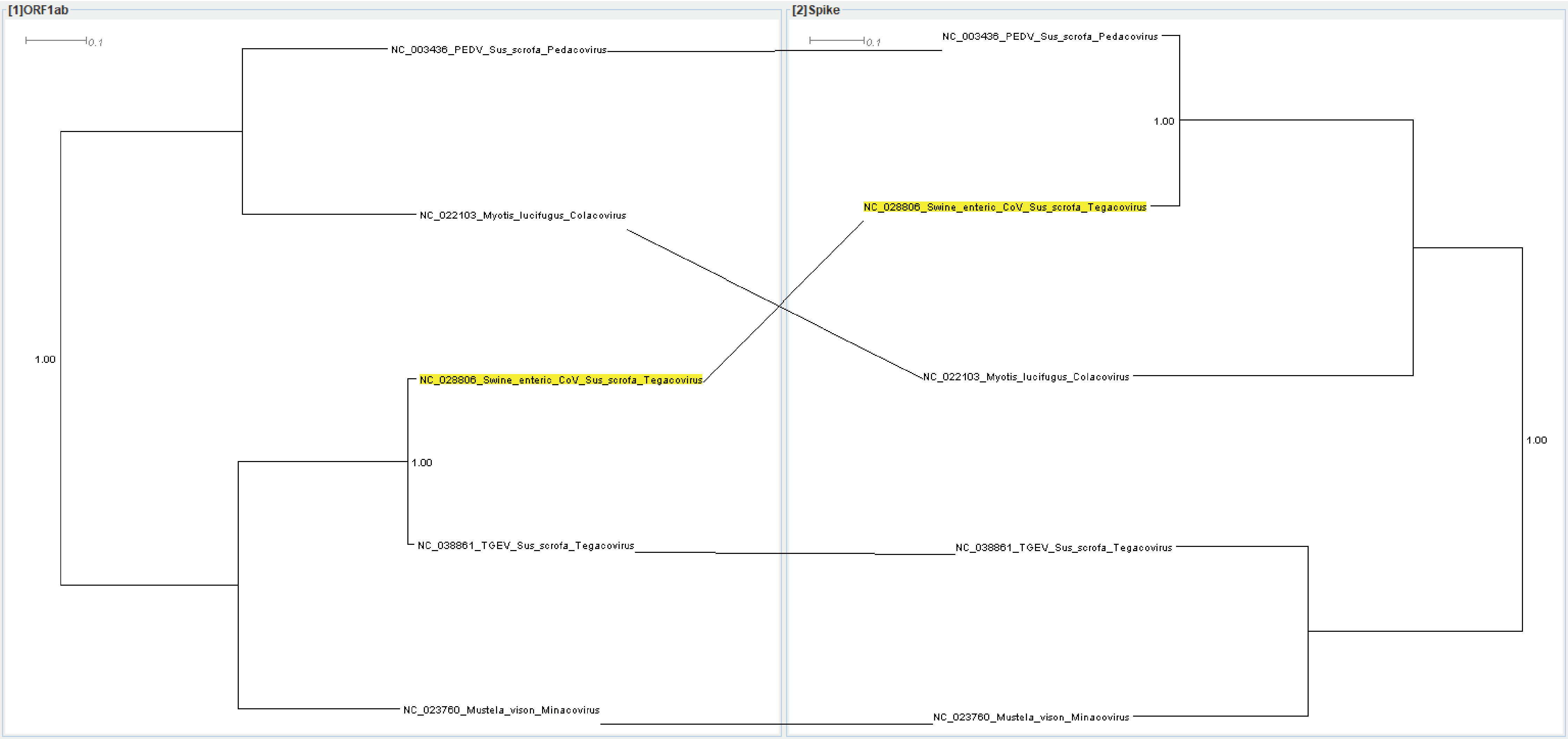

Trees	obs	au	np	bp	pp	kh	sh	wkh	wsh
Spike	-3826.8	1	1	1	1	1	1	1	1
ORF1ab	3826.8	8.00E-98	3.00E-24	0	0	0	0	0	0

Recombinant organisms in CONSEL analyses are colored yellow

Supp. fig. 26: Recombination event 7 Similarity plot and Bootscan analyses

A) *Alphacoronavirus Tegacovirus* (TGEV) Simplot:

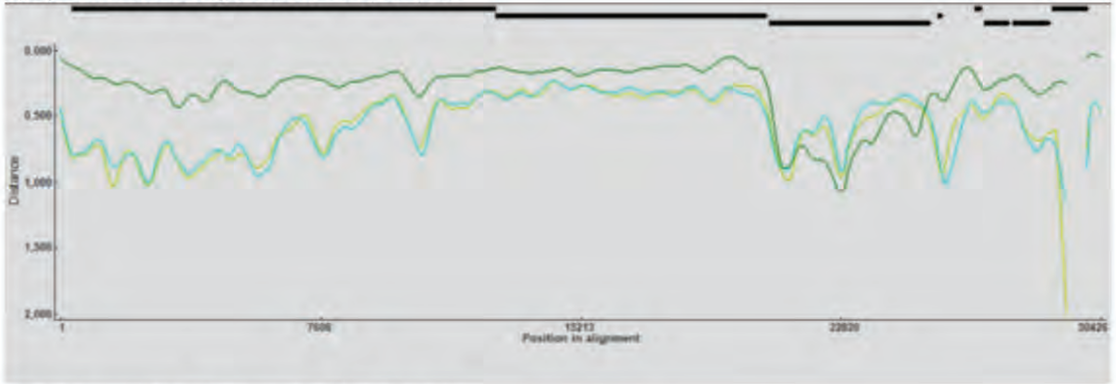

B) *Alphacoronavirus Tegacovirus* (TGEV) Bootscan:

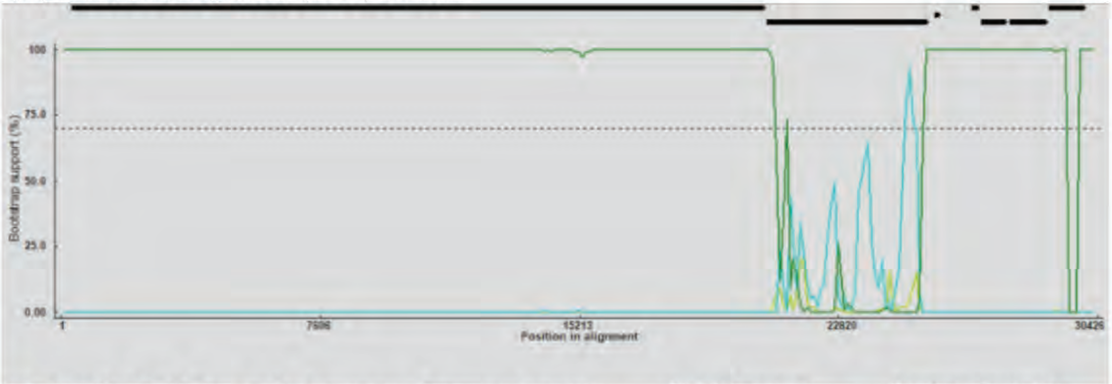

NC_038861_TGEV_Sus_scrofa_Tegacovrus_Ex scanned against:
NC_030292_Mustela_putorius_Minacovirus
NC_023760_Mustela_vison_Minacovirus_Ex
DQ848678_Felis_catus

Simplot params:
Window size:500
Step size:100

Bootscan params:
Window size:500
Step size:200
Bootstrap replicates:100
Pairwise distances

C) *Alphacoronavirus Tegacovirus* ORF1ab vs Spike Tanglegram

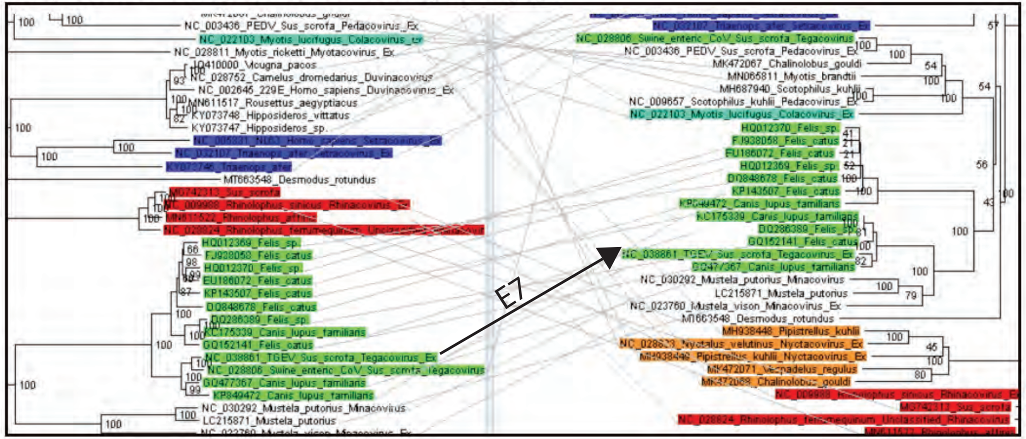

Supp. fig. 27: Event 7 CONSEL (ORF1ab LGIG - Spike WAGIG PhyML, aLRT, SPR)

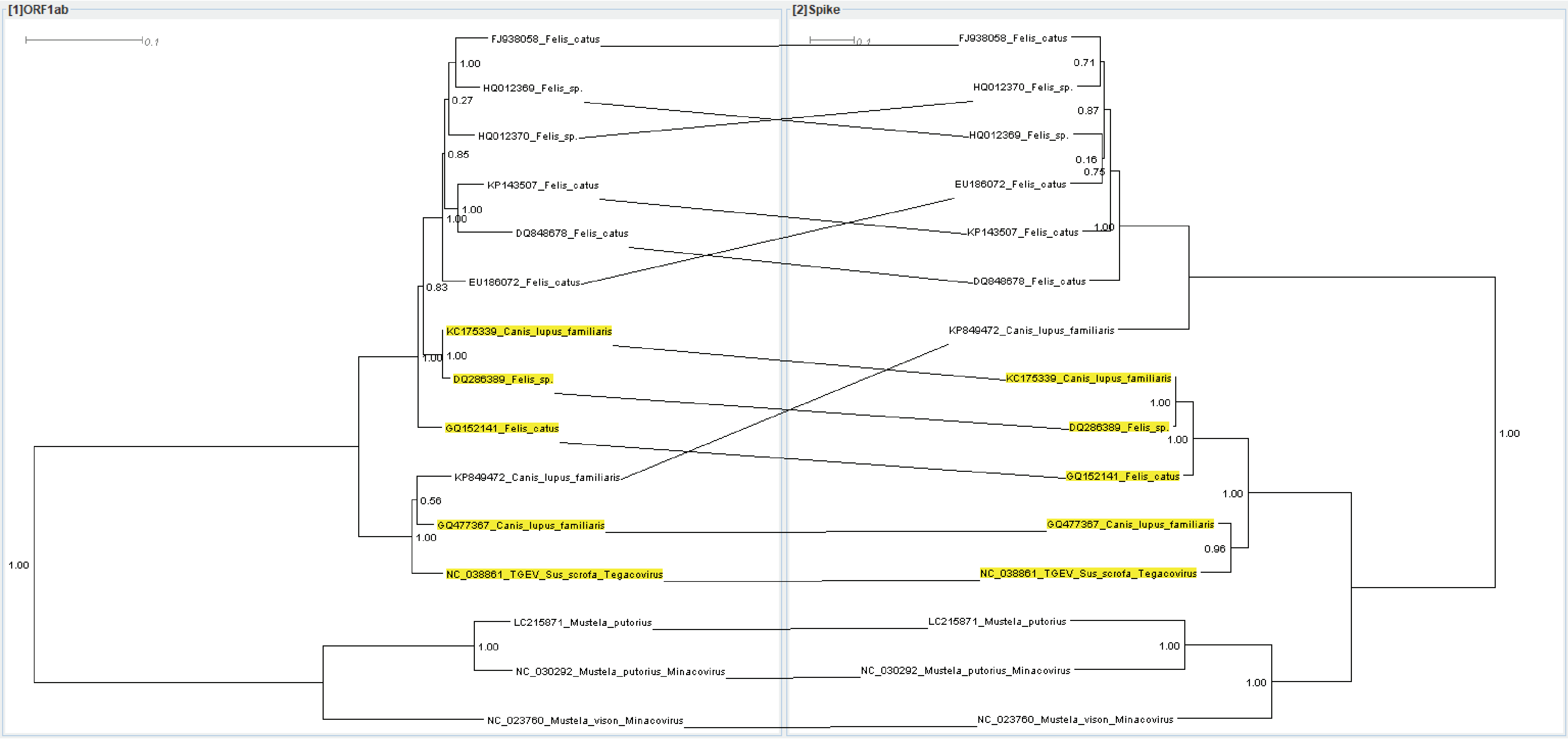

Trees	obs	au	np	bp	pp	kh	sh	wkh	wsh
Spike	-5594.2	1	1	1	1	1	1	1	1
ORF1ab	5594.2	2.00E-48	4.00E-16	0	0	0	0	0	0

Recombinant organisms in CONSEL analyses are colored yellow

Supp. fig. 28: *Alphacoronavirus* ORF1ab - Envelope BioNJ Tanglegram (Event 11)

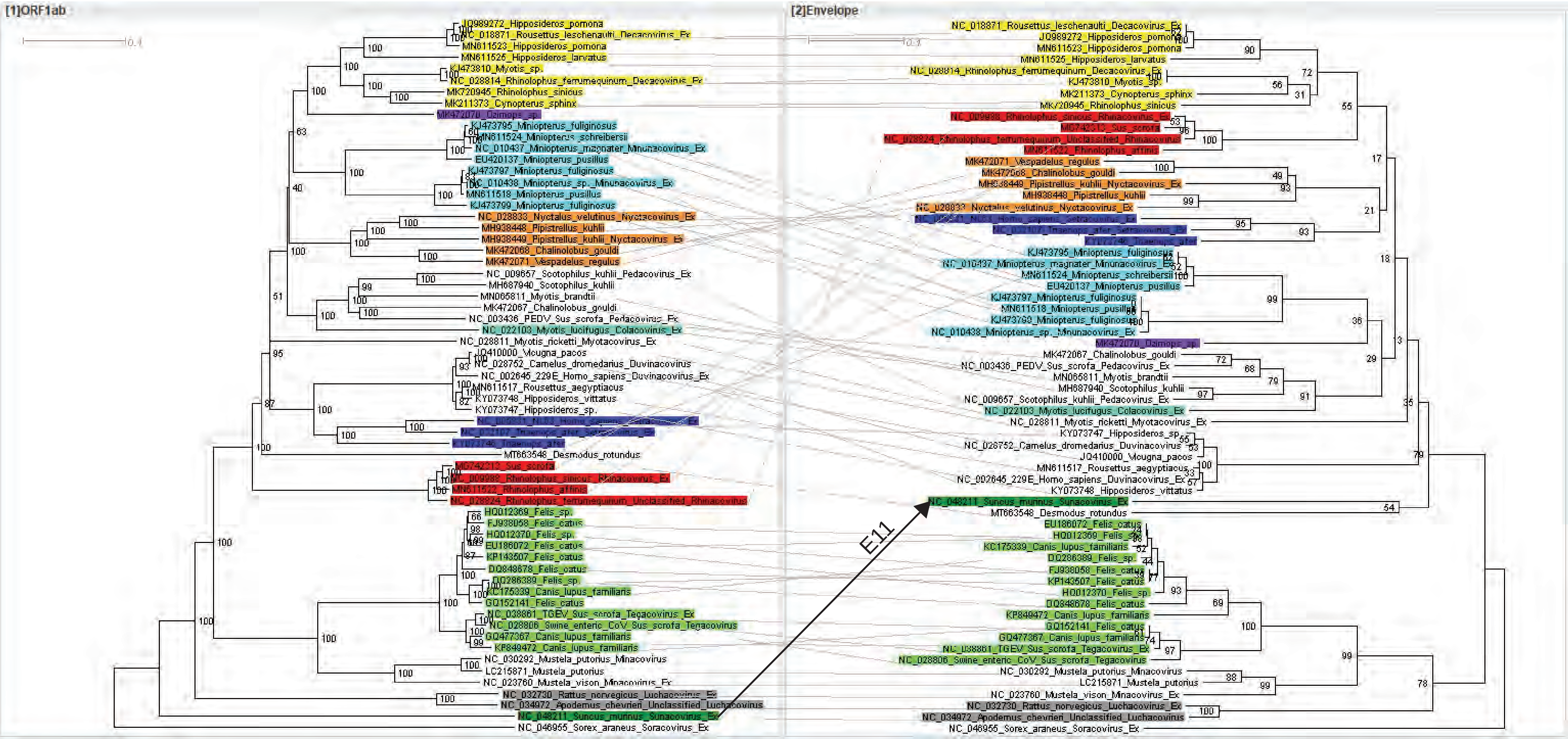

Supp. fig. 29: *Alphacoronavirus* ORF1ab - Envelope PhyML Tanglegram (Event 11)

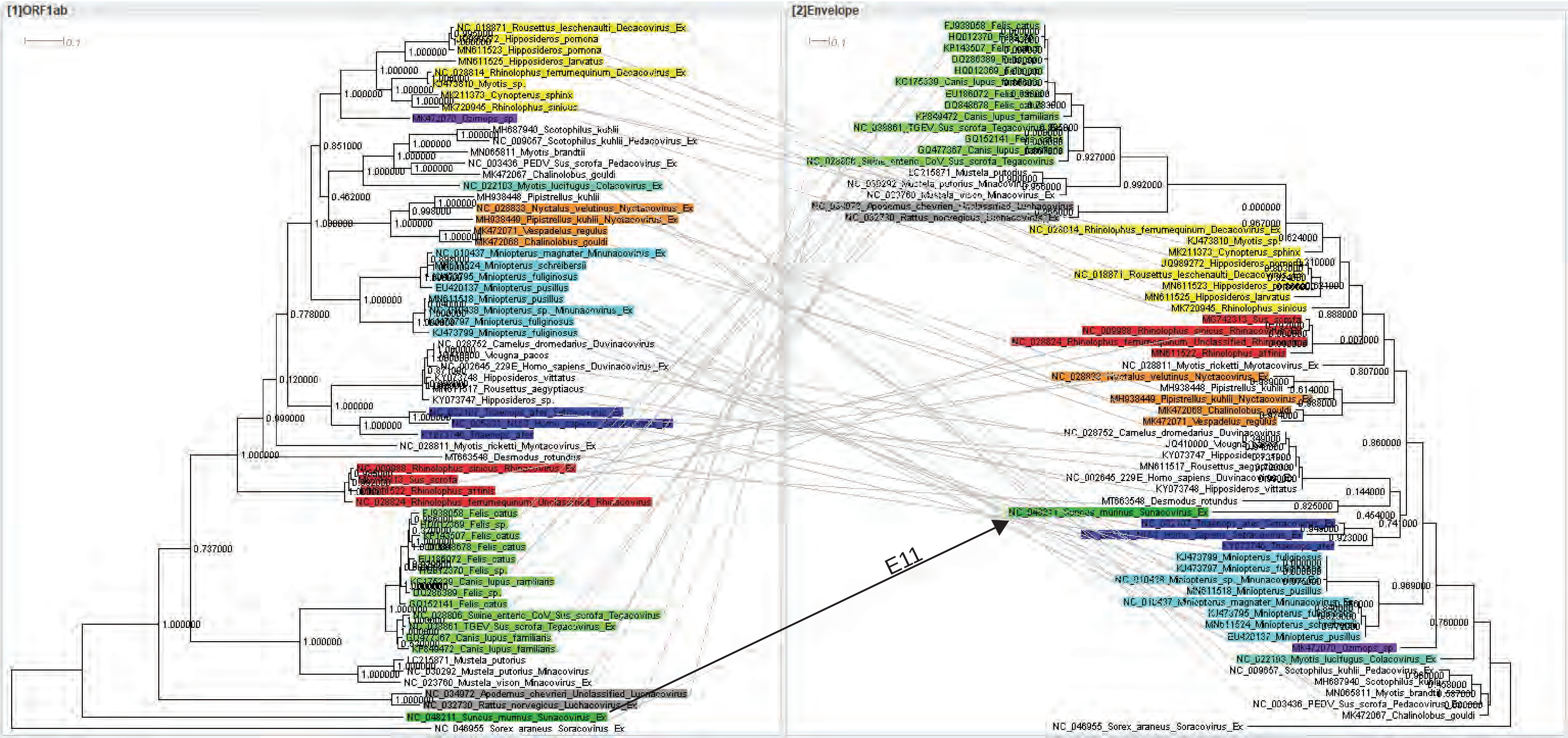

Supp. fig. 30: *Alphacoronavirus* ORF1ab - Envelope Bayesian Tanglegram (Event 11)

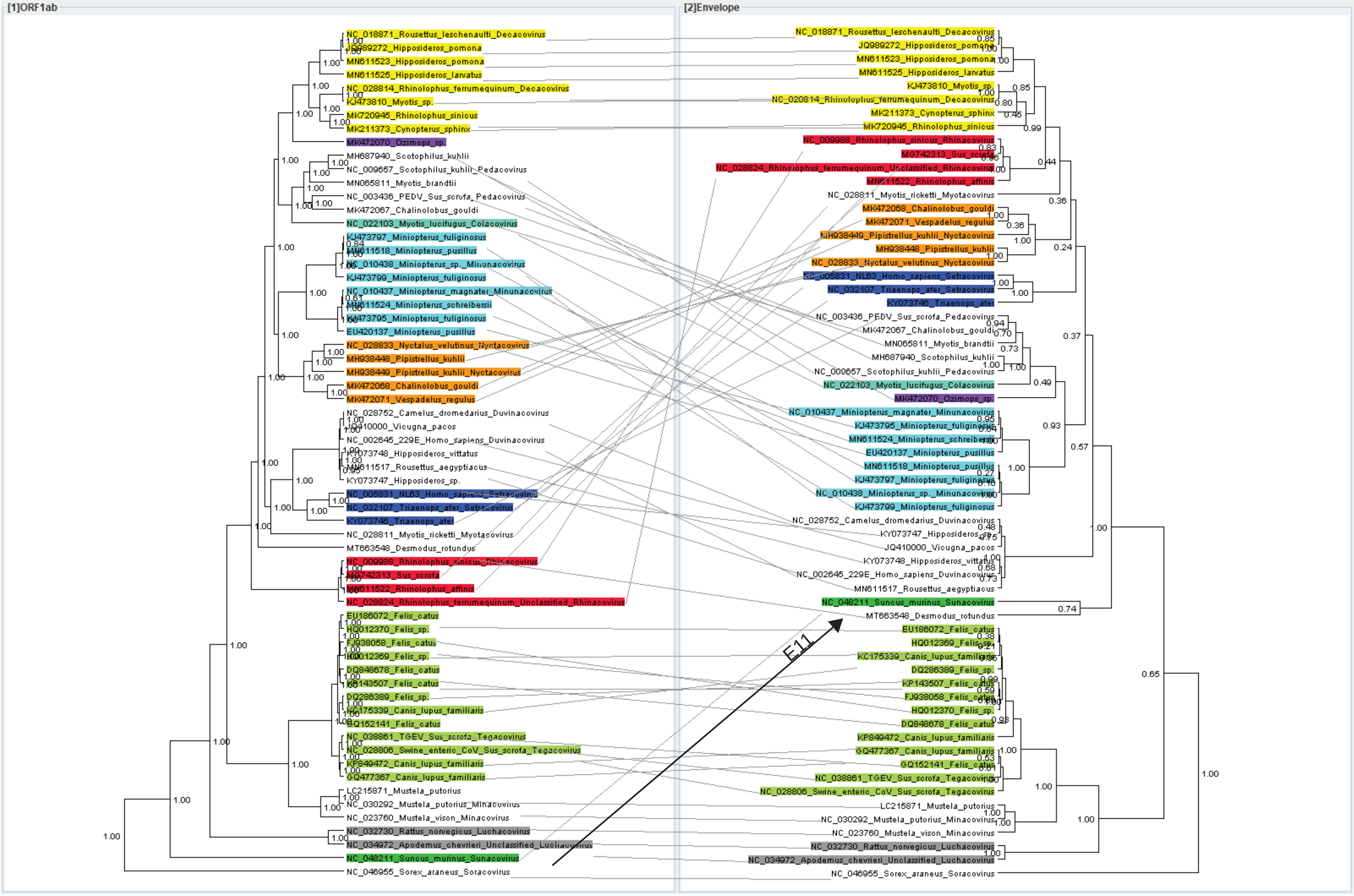

Supp. fig. 31: Event 11 CONSEL (ORF1ab LGIG - Envelope LGIG PhyML, aLRT, SPR)

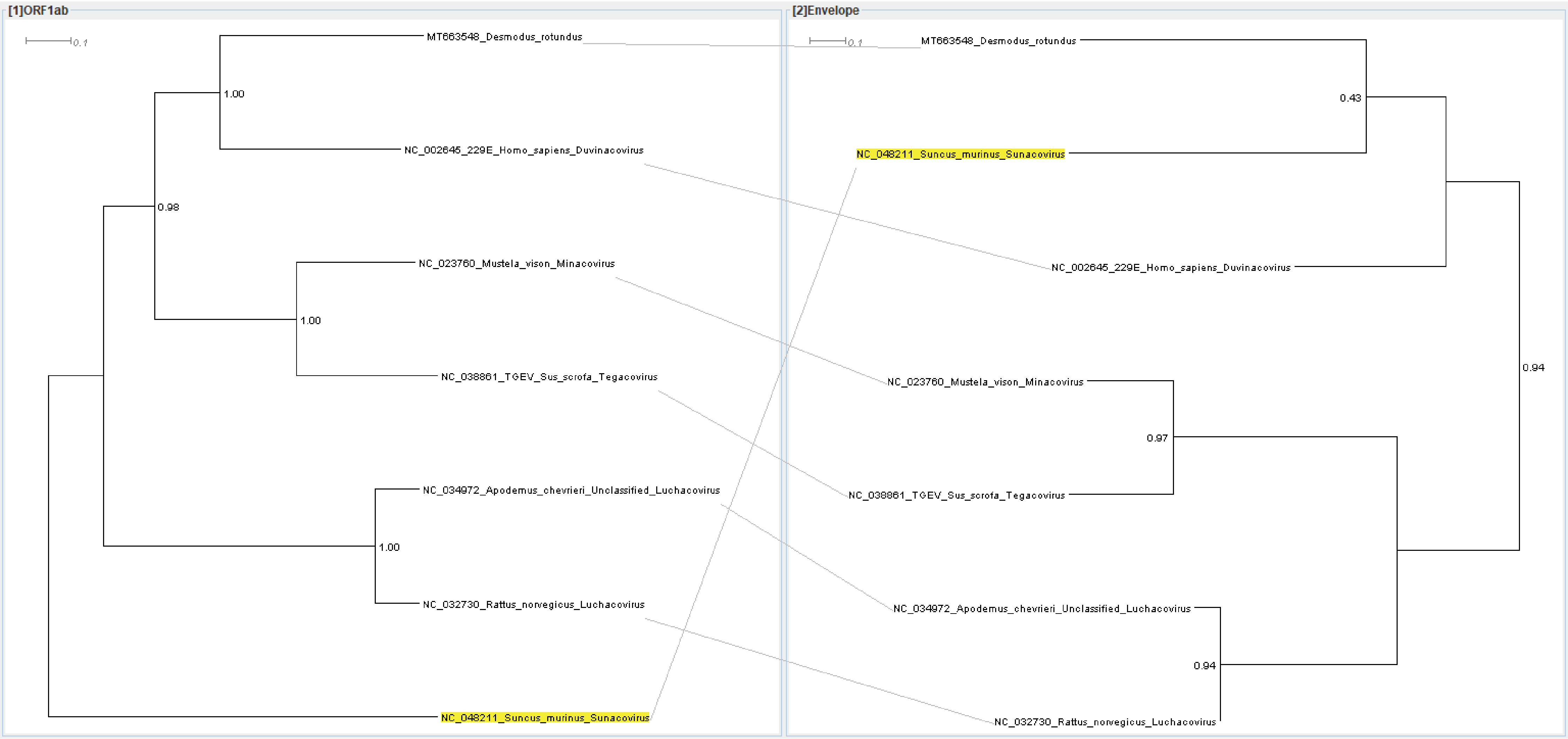

Tree	obs	au	np	bp	pp	kh	sh	wkh	wsh
Envelope	-29.8	0.999	0.999	0.999	1	0.998	0.998	0.998	0.998
ORF1ab	29.8	0.001	0.001	0.001	1.00E-13	0.002	0.002	0.002	0.002

Recombinant organisms in CONSEL analyses are colored yellow

Supp. fig 32: *Alphacoronavirus* BioNJ Robinson-Foulds matrix

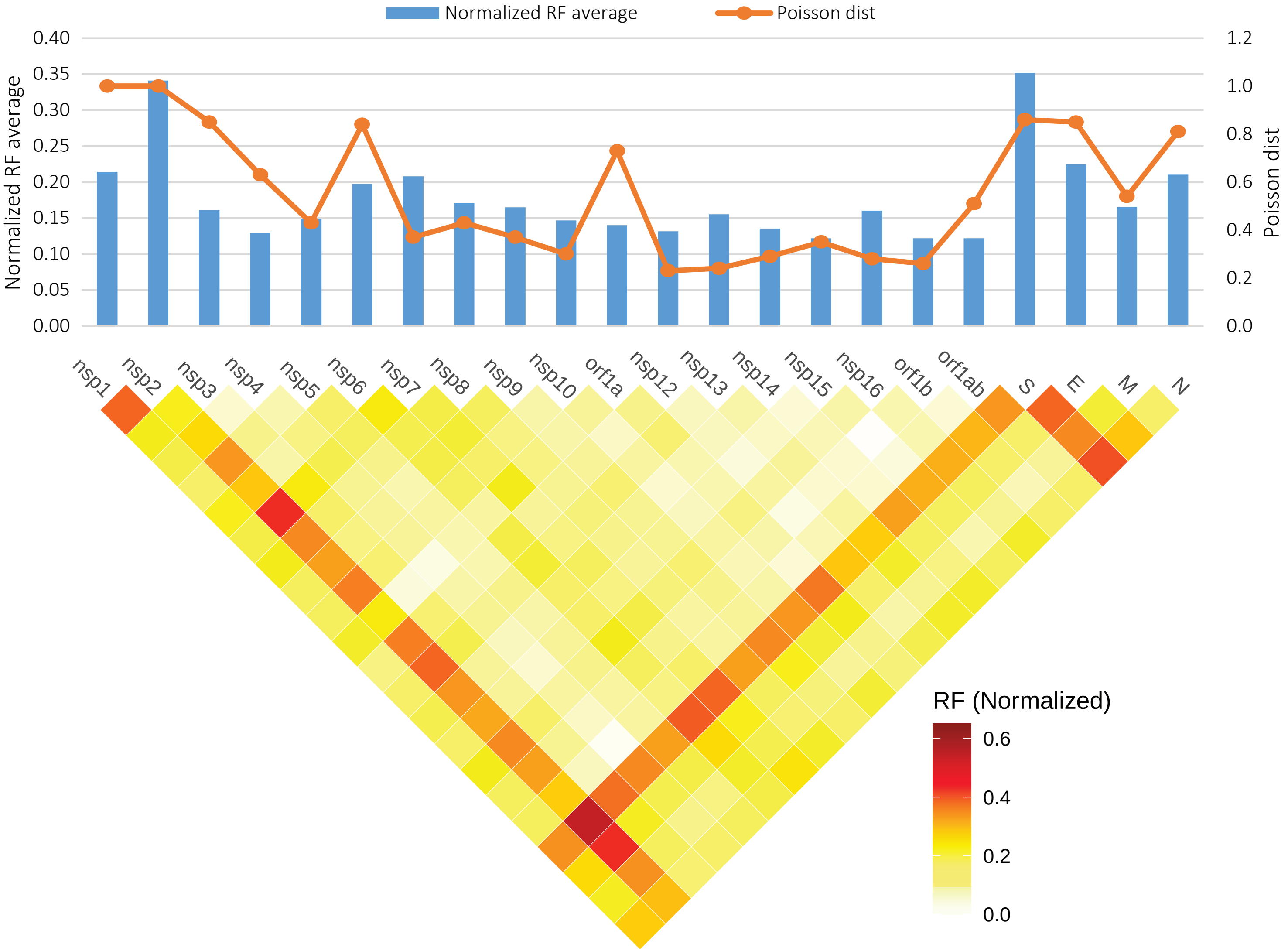

Alphacoronavirus BioNJ collapsed br.length 0.02
Poisson model, 500bs

Supp. fig 33: *Alphacoronavirus* PhyML Robinson-Foulds matrix

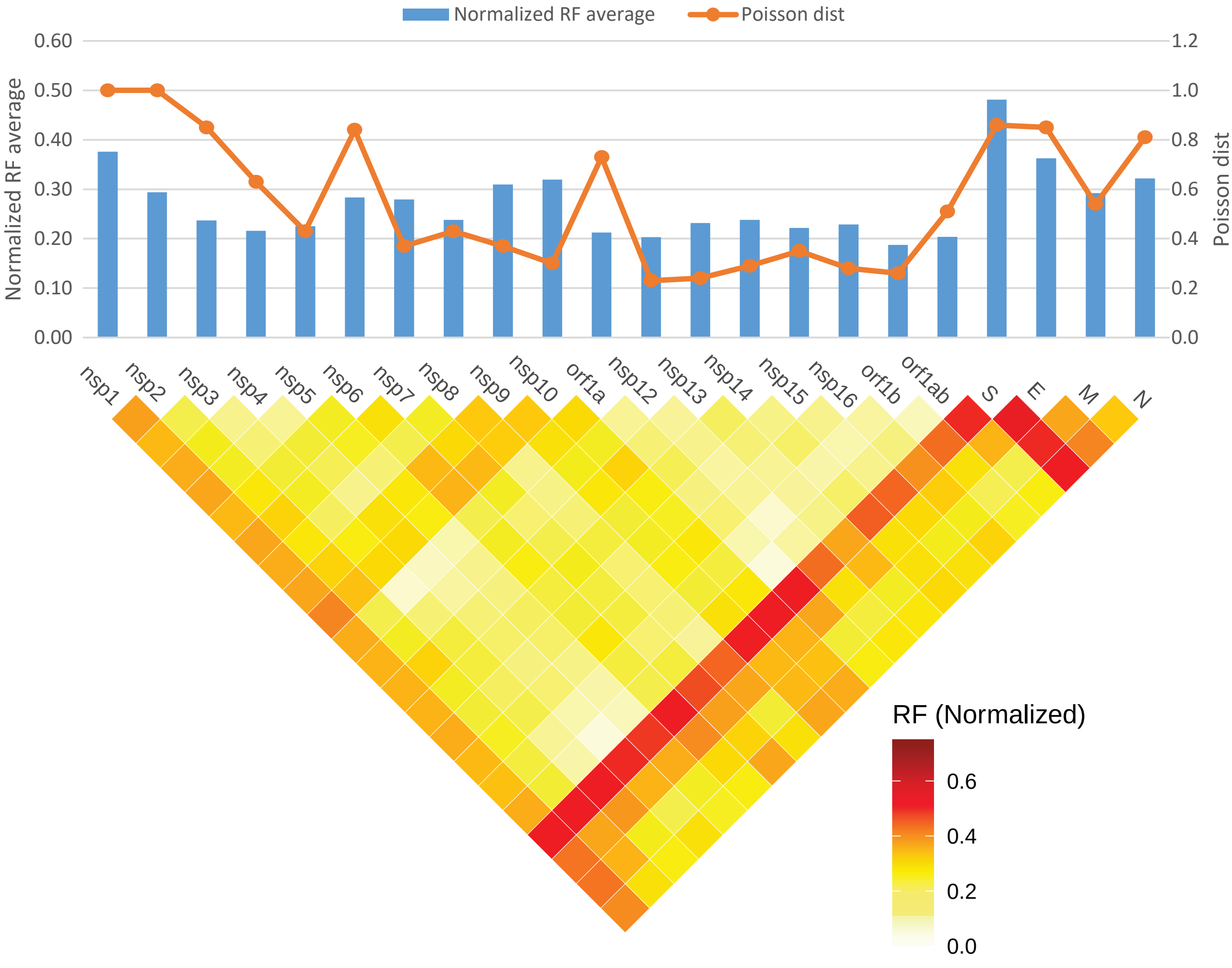

Alphacoronavirus PhyML collapsed br. length 0.02
PhyML model: LG+I+G

Supplementary file 1 section 2, subsection 2

Recombination events for β-CoVs

For easier inspection of the recombination events the β-CoV subgenera have been colored as follows

- *Sarbecovirus* subgenus
- *Nobecovirus* subgenus
- *Merbecovirus* subgenus
- *Embecovirus* subgenus

Phylogenetic incogruences are noted with arrows

Recombinant organisms in CONSEL analyses are colored yellow

- Event 12: *Merbecovirus* Nucleocapsid
  These peptides are now sister group to *Sarbecovirus* - *Hibecovirus* nucleocapsids. Bootstrap 93, aLRT 0.96, posterior probability 1. This is also supported by CONSEL

Supp. fig. 34: *Betacoronavirus* ORF1ab - Nucleocapsid BioNJ Tanglegram (Event 12)

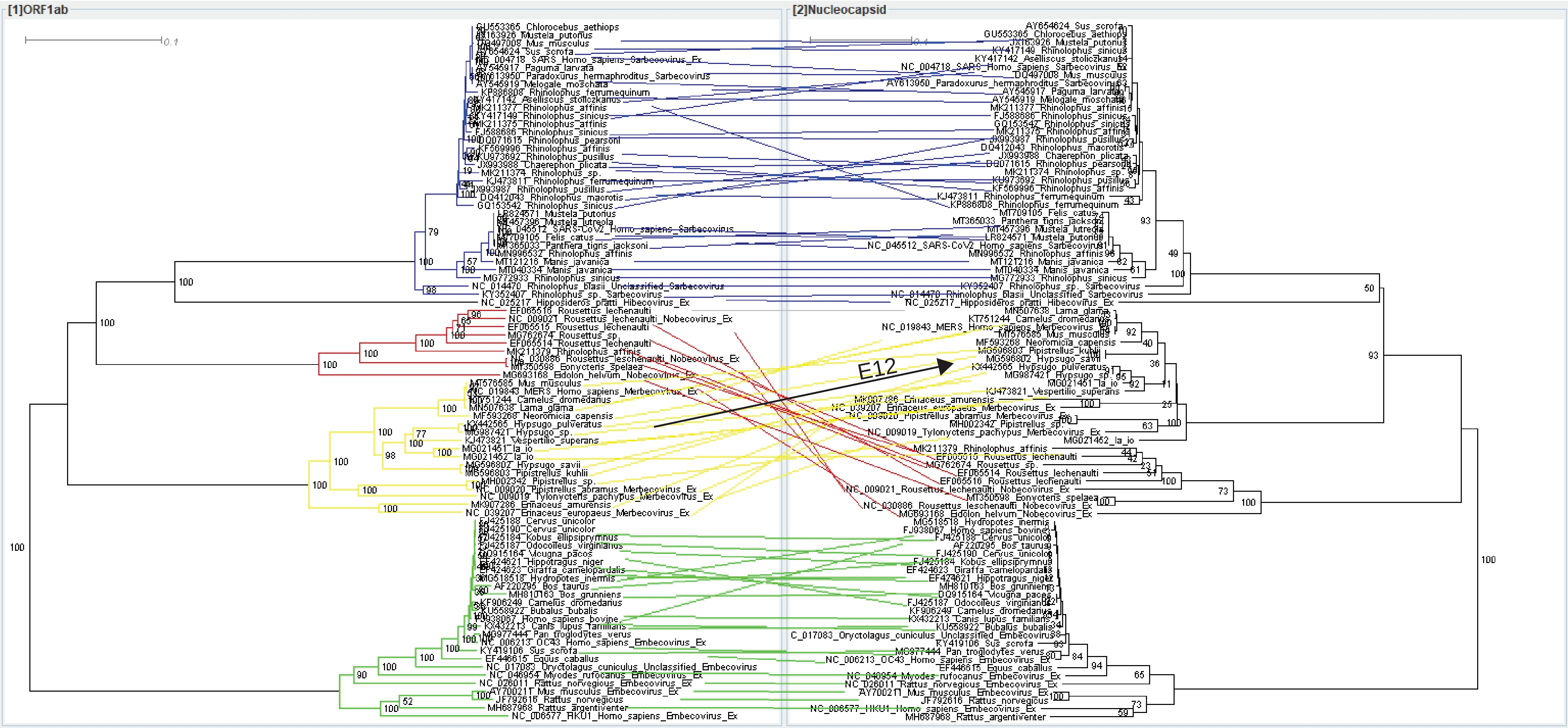

Supp. fig. 35: *Betacoronavirus* ORF1ab - Nucleocapsid PhyML Tanglegram (Event 12)

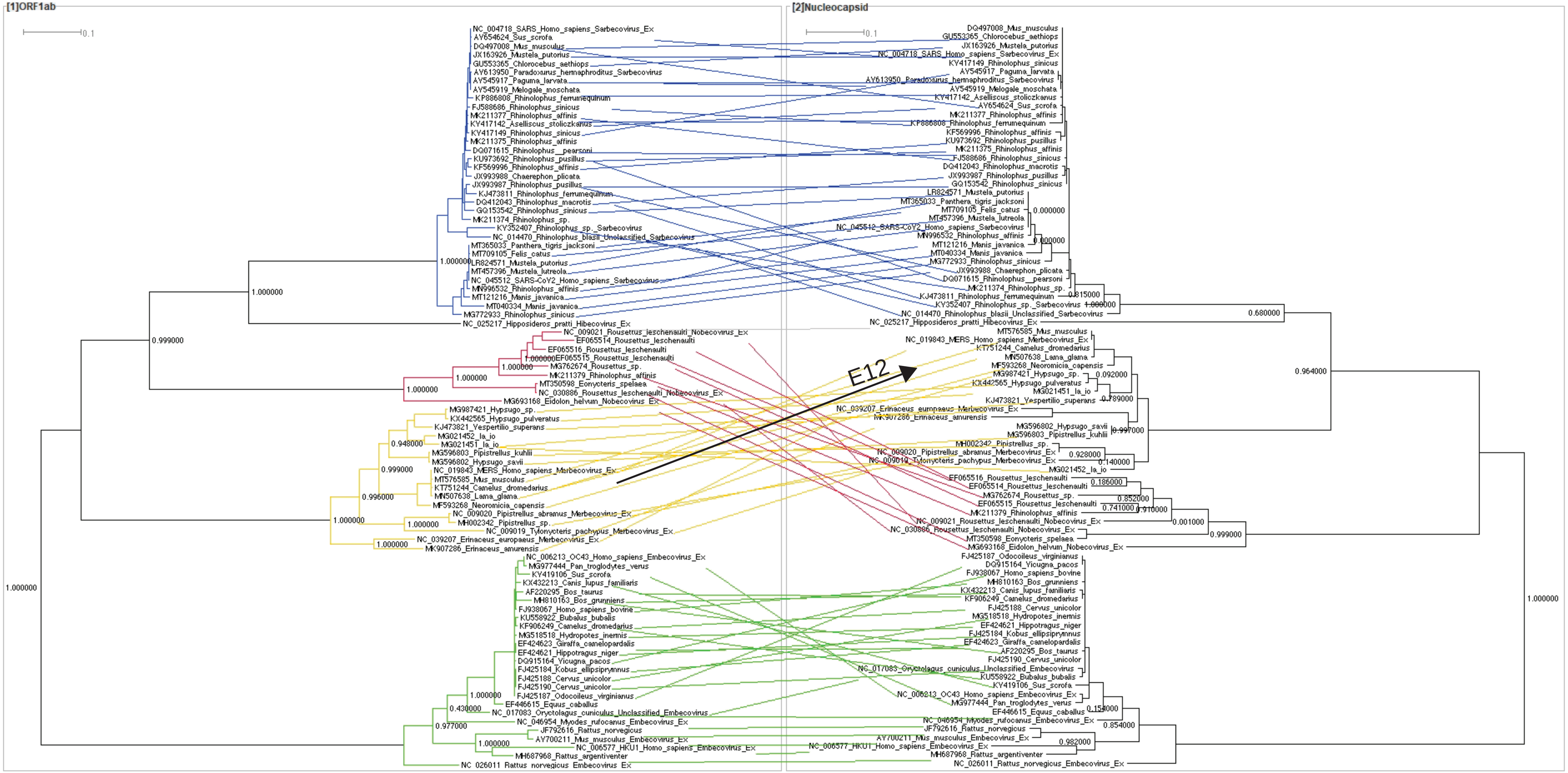

Supp. fig. 36: *Betacoronavirus* ORF1ab - Nucleocapsid Bayesian Tanglegram (Event 12)

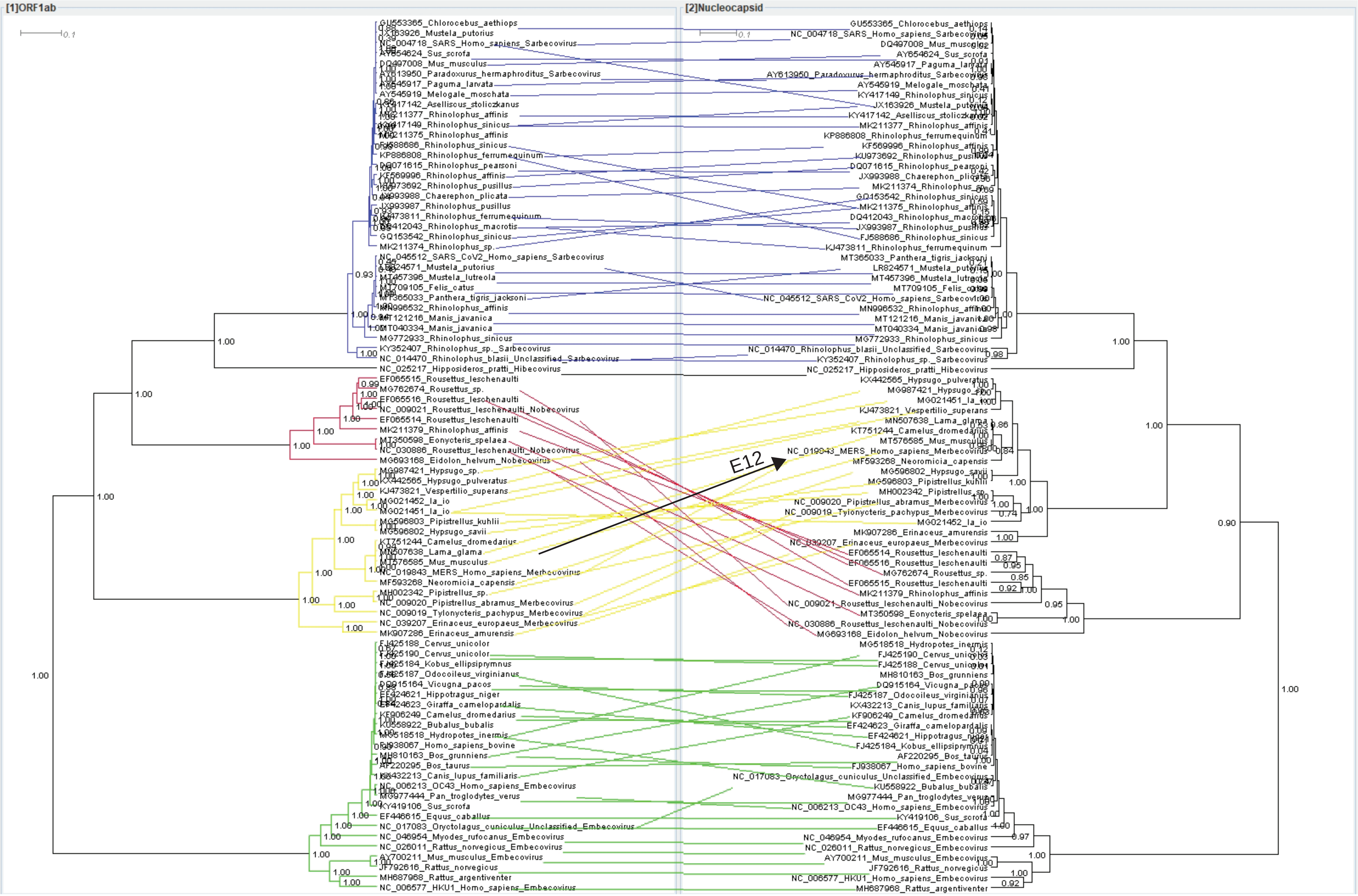

Supp. fig. 37: Event 12 CONSEL (ORF1ab LGIG - Nucleocapsid LGIG PhyML, aLRT, SPR)

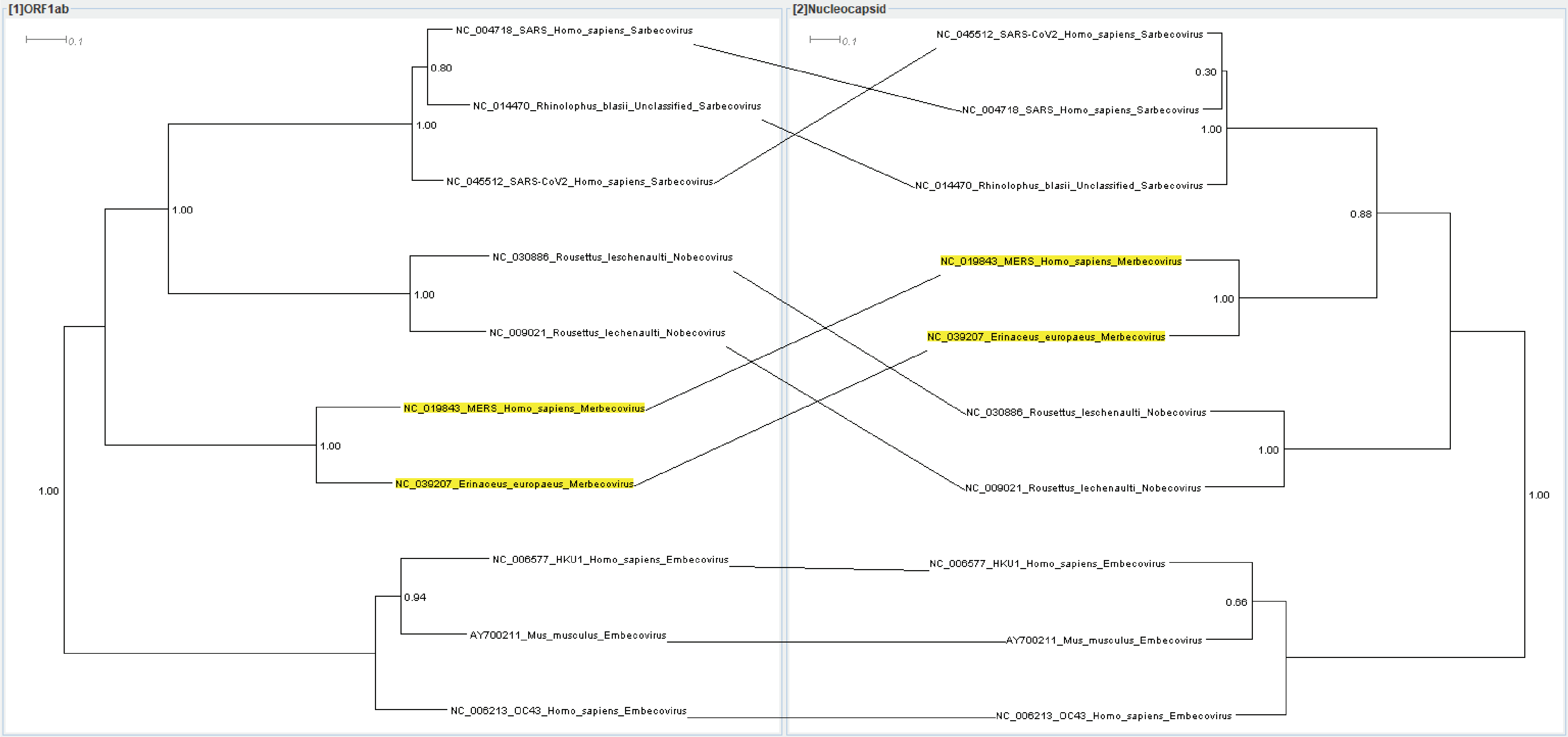

Trees	obs	au	np	bp	pp	kh	sh	wkh	wsh
Nucleocapsid	-28.7	0.998	0.998	0.998	1	0.997	0.997	0.997	0.997
ORF1ab	28.7	0.002	0.002	0.002	3.00E-13	0.003	0.003	0.003	0.003

Recombinant organisms in CONSEL analyses are colored yellow

Supp. fig. 38: *Betacoronavirus* BioNJ Robinson-Foulds matrix

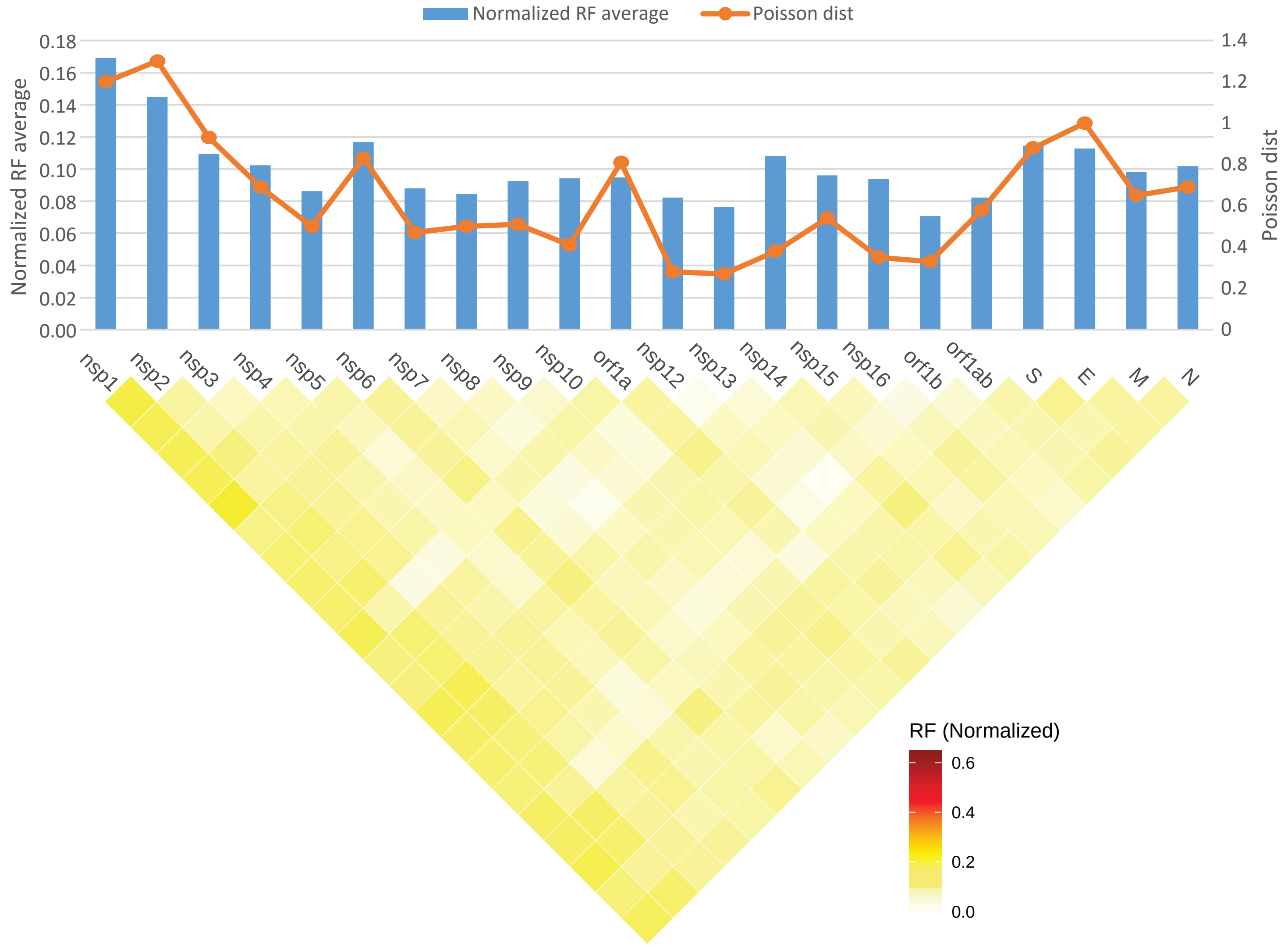

Betacoronavirus BioNJ collapsed br. length 0.02

Poisson model, 500bs

Supp. fig. 39: *Betacoronavirus* PhyML Robinson-Foulds matrix

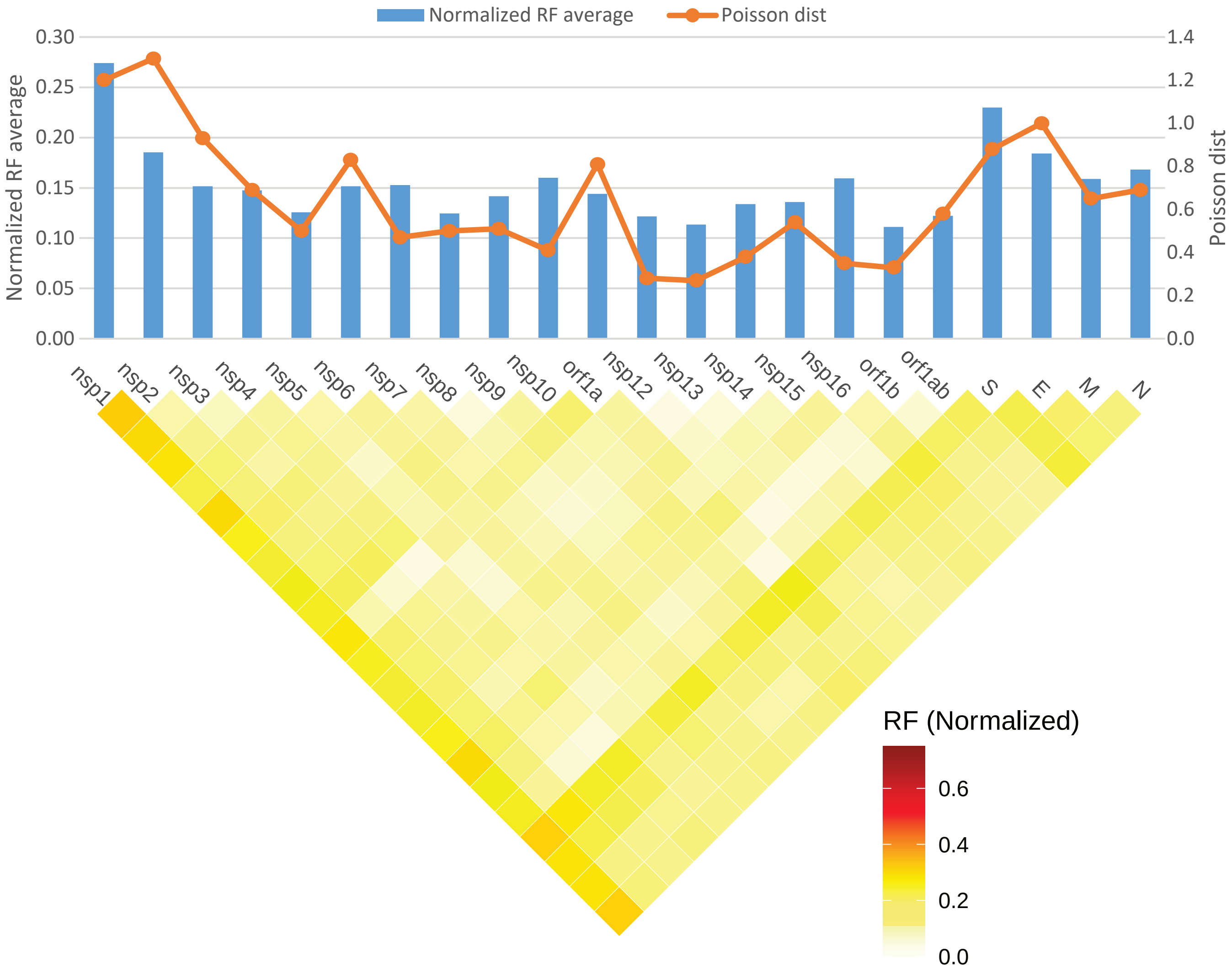

Betacoronavirus PhyML collapsed br. length 0.02
PhyML model: LG+I+G

Supplementary file 1 section 2, subsection 3

Recombination events for γ-CoVs

Recombinant *Igacovirus* sequences have been colored yellow

Phylogenetic incogruences are noted with arrows

Recombinant organisms in CONSEL analyses are colored yellow

- Event 13: *Igacovirus* Spike
  This event is about LN610099 *Numida.*

- Event 14: *Igacovirus* Spike
  This event is about KR822424 *Meleagris.*

- Event 15: *Igacovirus* Spike
  This event is about EU022525 *Meleagris.*

- Event 16: *Igacovirus* Spike
  This event is about KM454473 *Anatidae.*

In events 13 - 16 four *Igacovirus* sequences form a monophyletic group outside of the *Igacovirus* subgenus and each one is considered a separate recombination event with an uknknown γ-CoV donor. These events are supported by all three trees (Boostrap support 100, aLRT 1 and posterior probability 0.94). These events are also supported by Simplot, Bootscan and CONSEL

- Event 17: *Igacovirus Anatidae sp.* Membrane
  KM454473 *Anatidae* membrane is no longer within the *Igacovirus* subgenus and is now sister group to *Brangacovirus*. Bootstrap support 73, aLRT 0.92, posterior probability 0.99. This event is also supported by CONSEL

Supp. fig. 40: *Gammacoronavirus* ORF1ab - Spike BioNJ Tanglegram (Events 13-16)

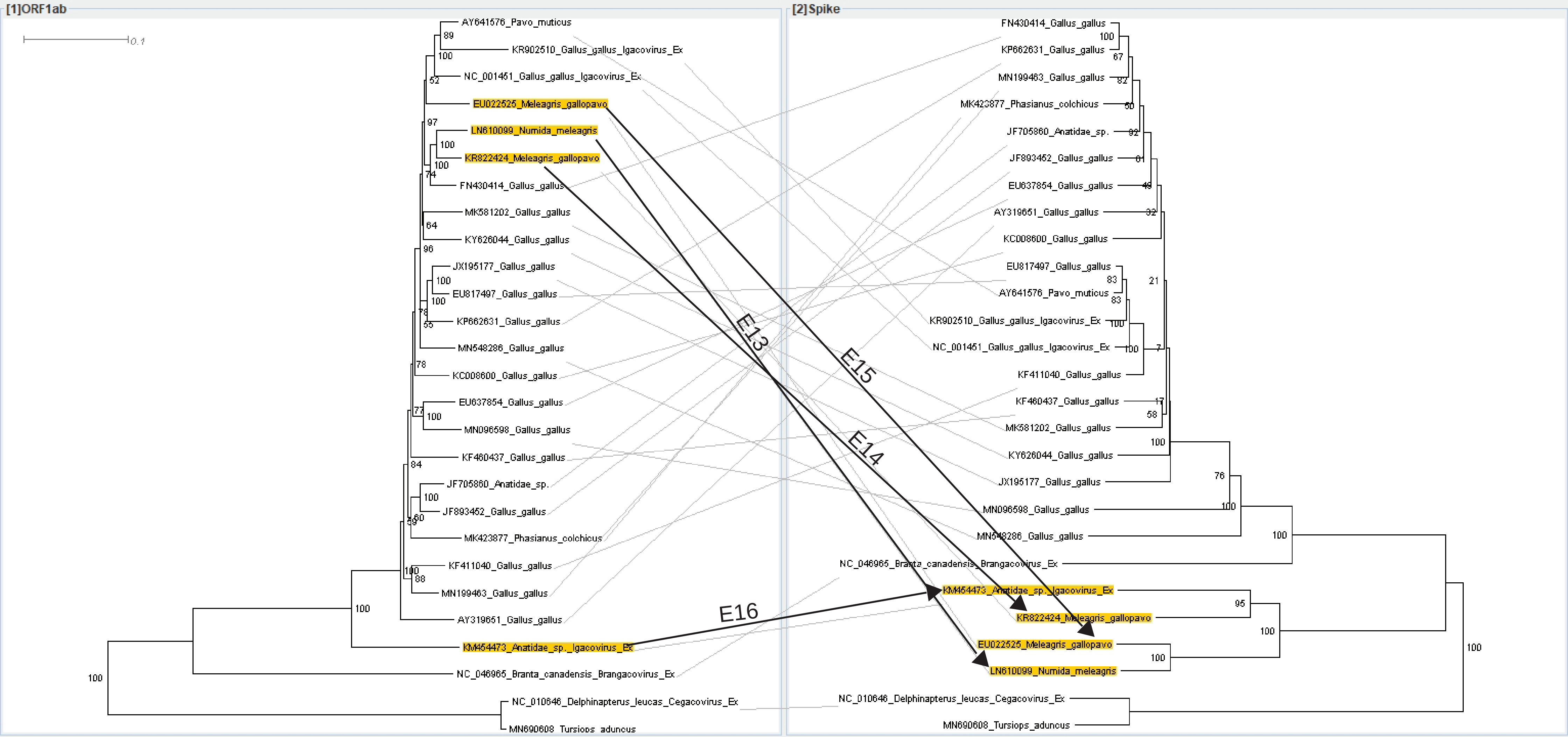

Supp. fig. 41: *Gammacoronavirus* ORF1ab - Spike PhyML Tanglegram (Events 13-16)

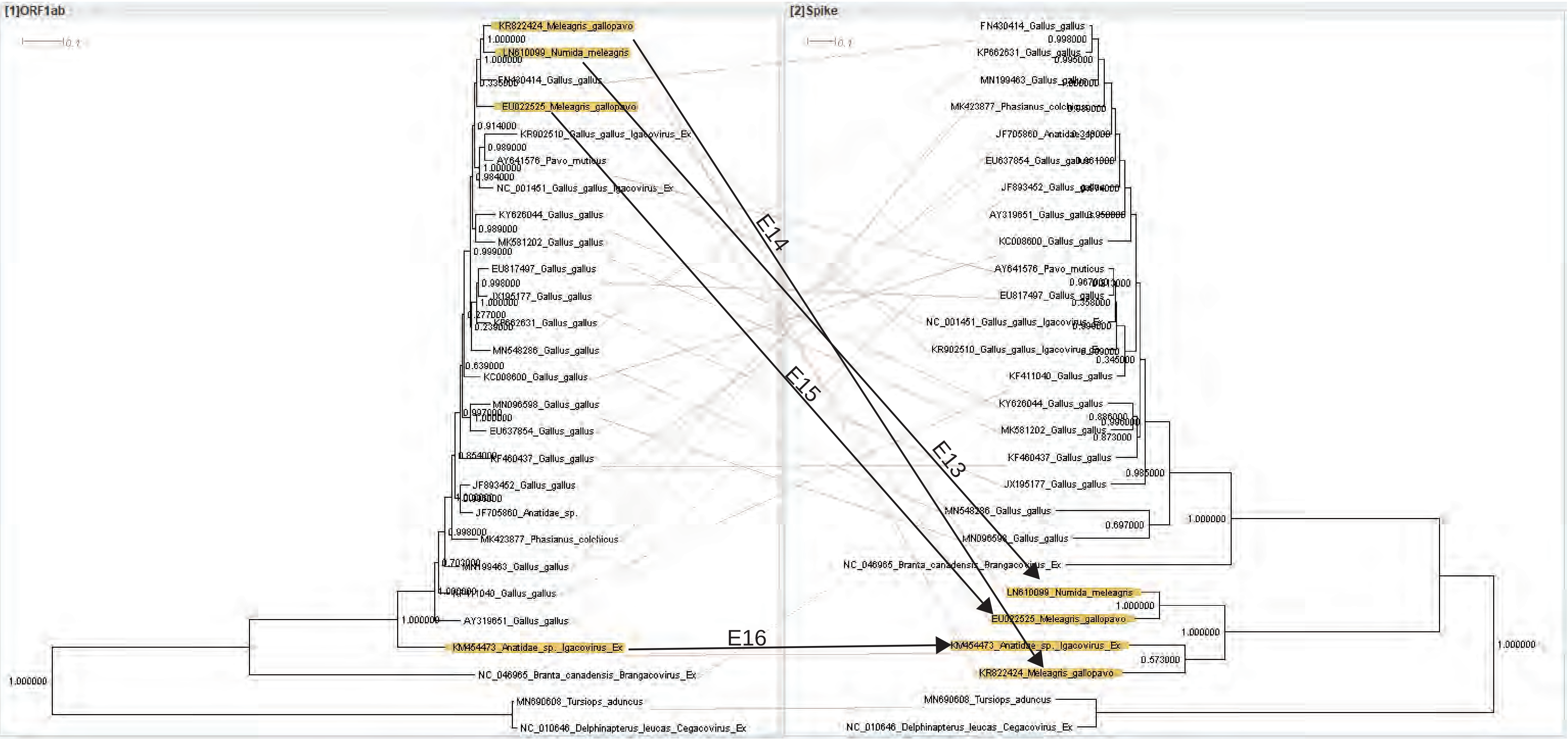

Supp. fig. 42: *Gammacoronavirus* ORF1ab - Spike Bayesian Tanglegram (Events 13-16)

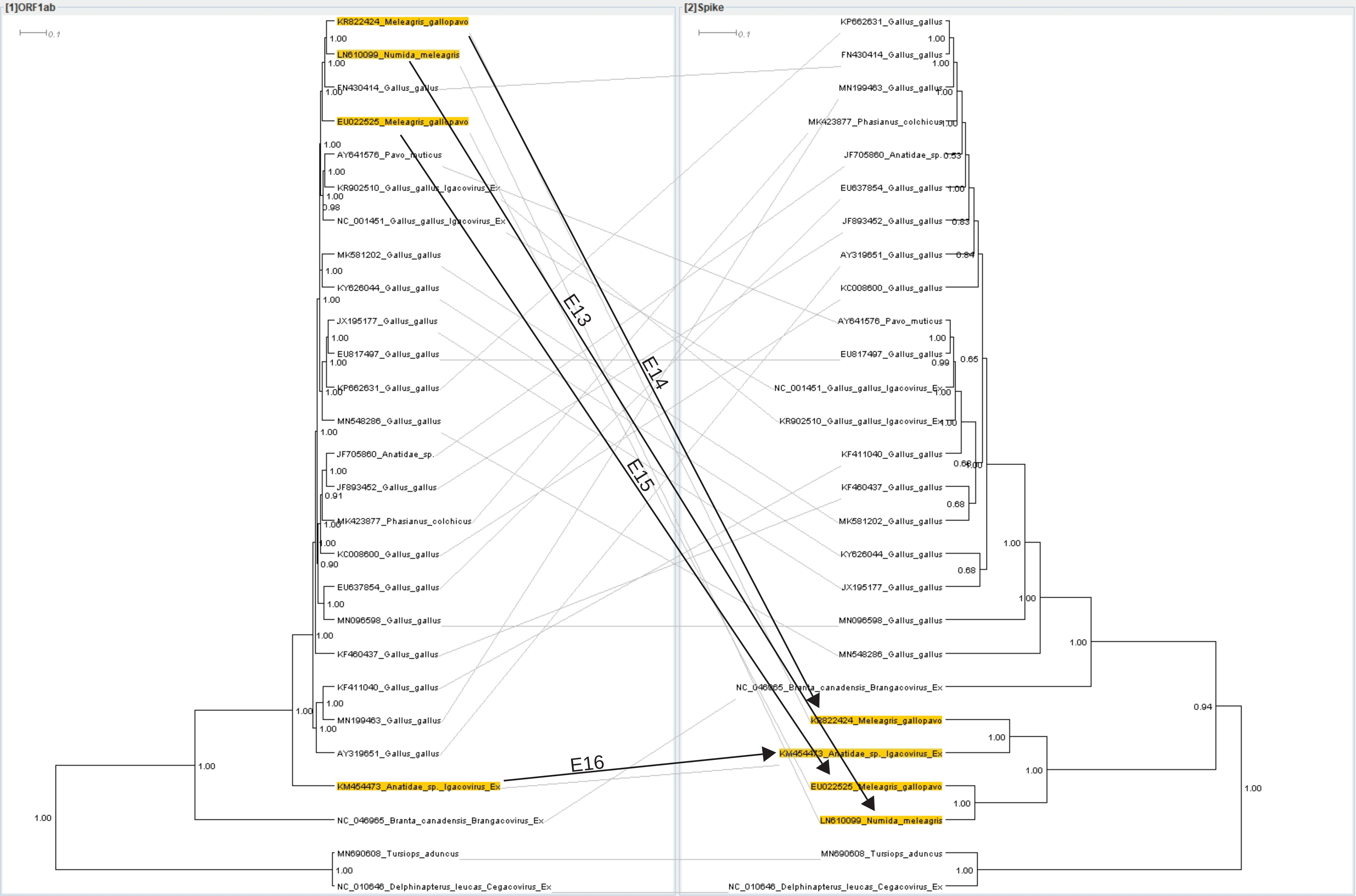

Supp. fig. 43: Recombination events 16-17 Simplot and Bootscan analyses

A) *Gammacoronavirus Igacovirus* Simplot:

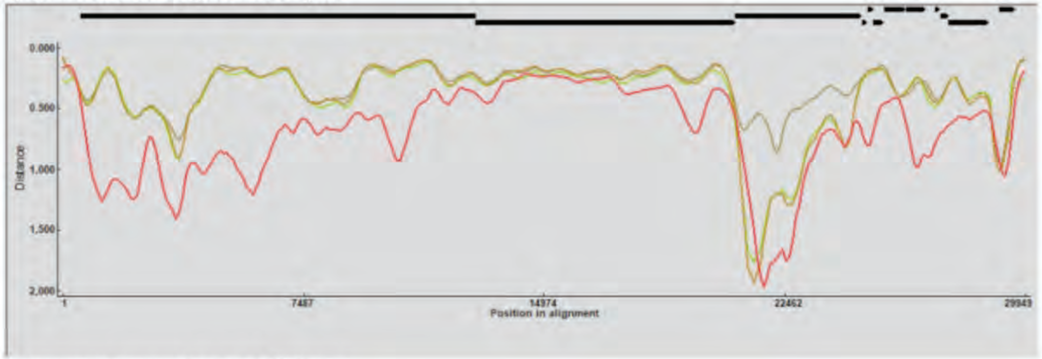

B) *Gammacoronavirus Igacovirus* Bootscan:

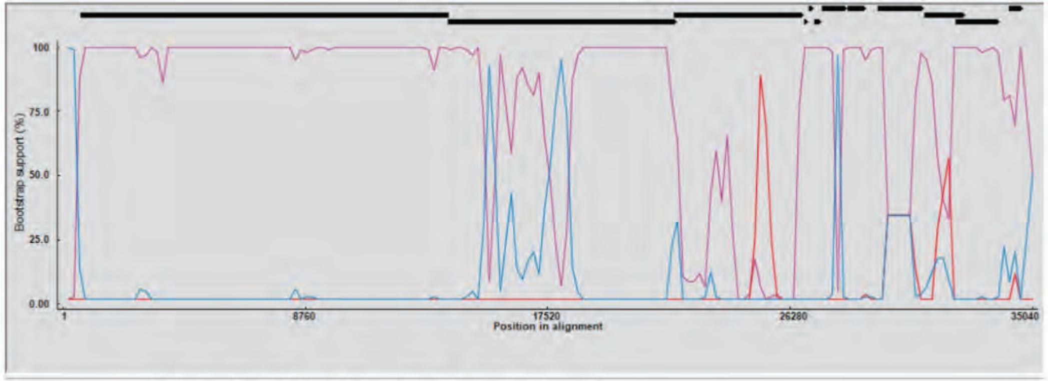

C) *Gammacoronavirus* recombinant *Igacovirus* ORF1ab vs Spike Tanglegram

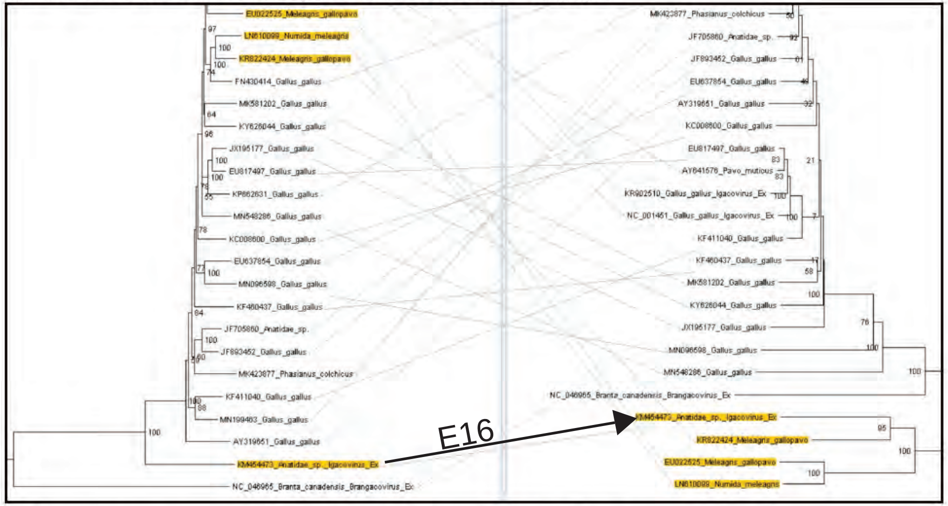

D) *Gammacoronavirus* recombinant *Igacovirus* ORF1ab vs Membrane Tanglegram

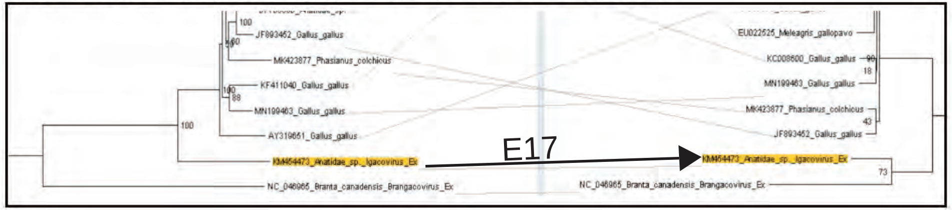

KM454473_Anatidae_sp._Igacovirus_Ex scanned against:

Simplot

- KR822424_Meleagris_gallopavo
- KR902510_Gallus_gallus_Igacovirus_Ex
- NC_001451_Gallus_gallus_Igacovirus_Ex
- NC_046965_Branta_canadensis_Brangacovirus_Ex

Bootscan

- KR902510_Gallus_gallus_Igacovirus_Ex
- NC_010646_Delphinapterus_leucas_Cegacovirus_Ex
- NC_046965_Branta_canadensis_Brangacovirus_Ex

Simplot params:
Window size:500
Step size:100

Bootscan params:
Window size:500
Step size:200
Bootstrap replicates:100
Pairwise distances

Supp. fig. 44: Events 13-16 CONSEL (ORF1ab WAGG - Spike WAGIG PhyML, aLRT, SPR)

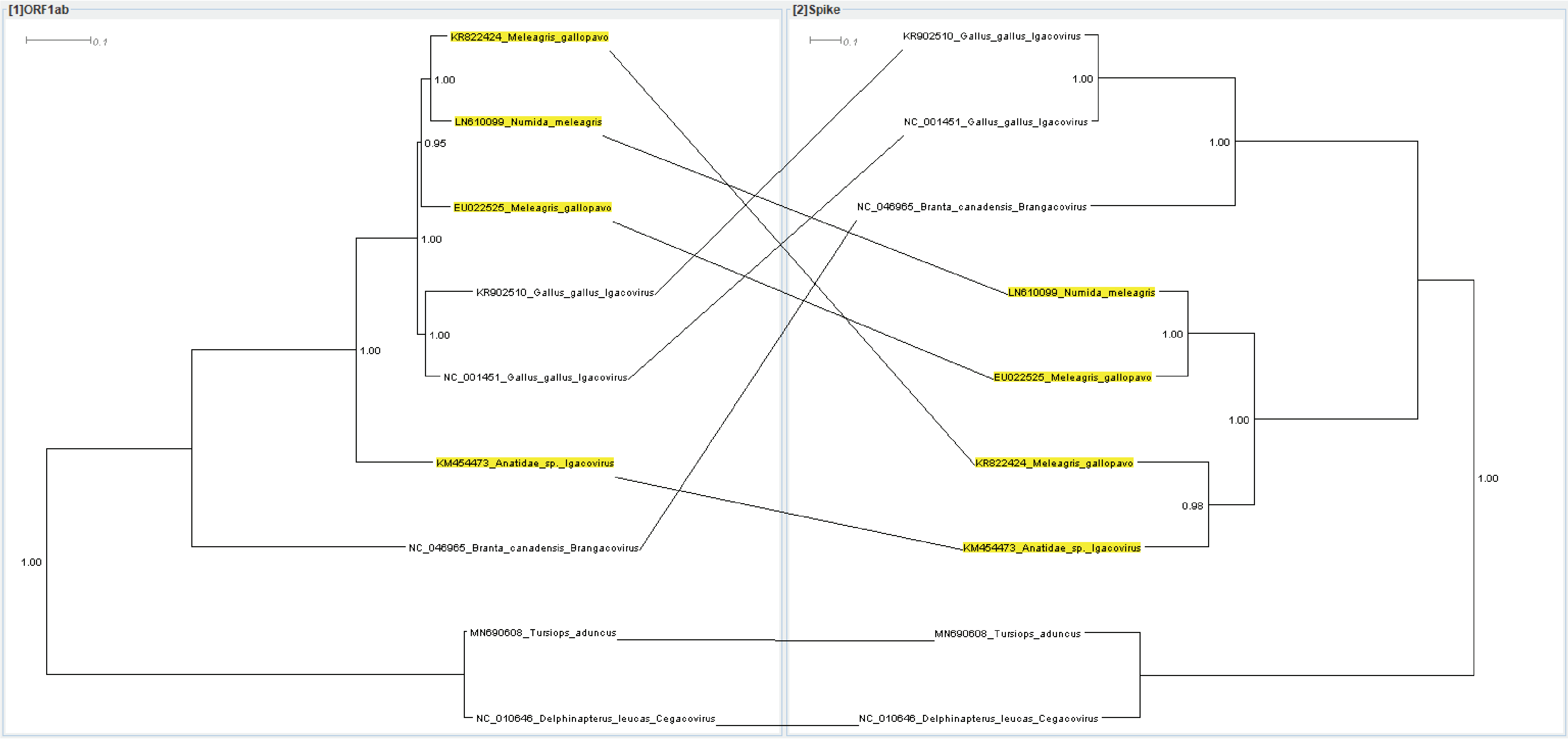

Tree	obs	au	np	bp	pp	kh	sh	wkh	wsh
Spike	-4084.7	1	1	1	1	1	1	1	1
ORF1ab	4084.7	2.00E-77	1.00E-21	0	0	0	0	0	0

Recombinant organisms in CONSEL analyses are colored yellow

Supp. fig. 45: *Gammacoronavirus* ORF1ab - Membrane BioNJ Tanglegram (Event 17)

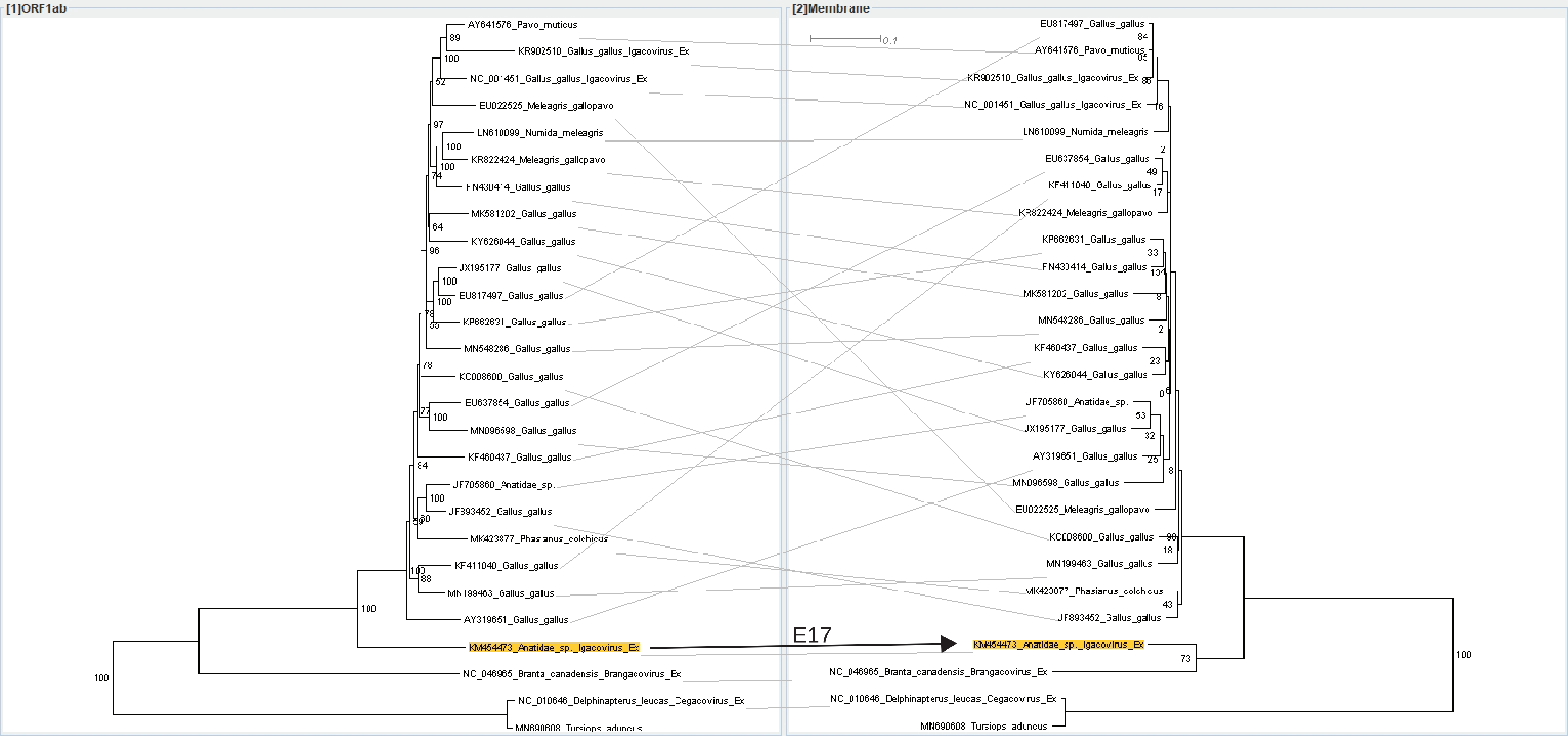

Supp. fig. 46: *Gammacoronavirus* ORF1ab - Membrane PhyML Tanglegram (Event 17)

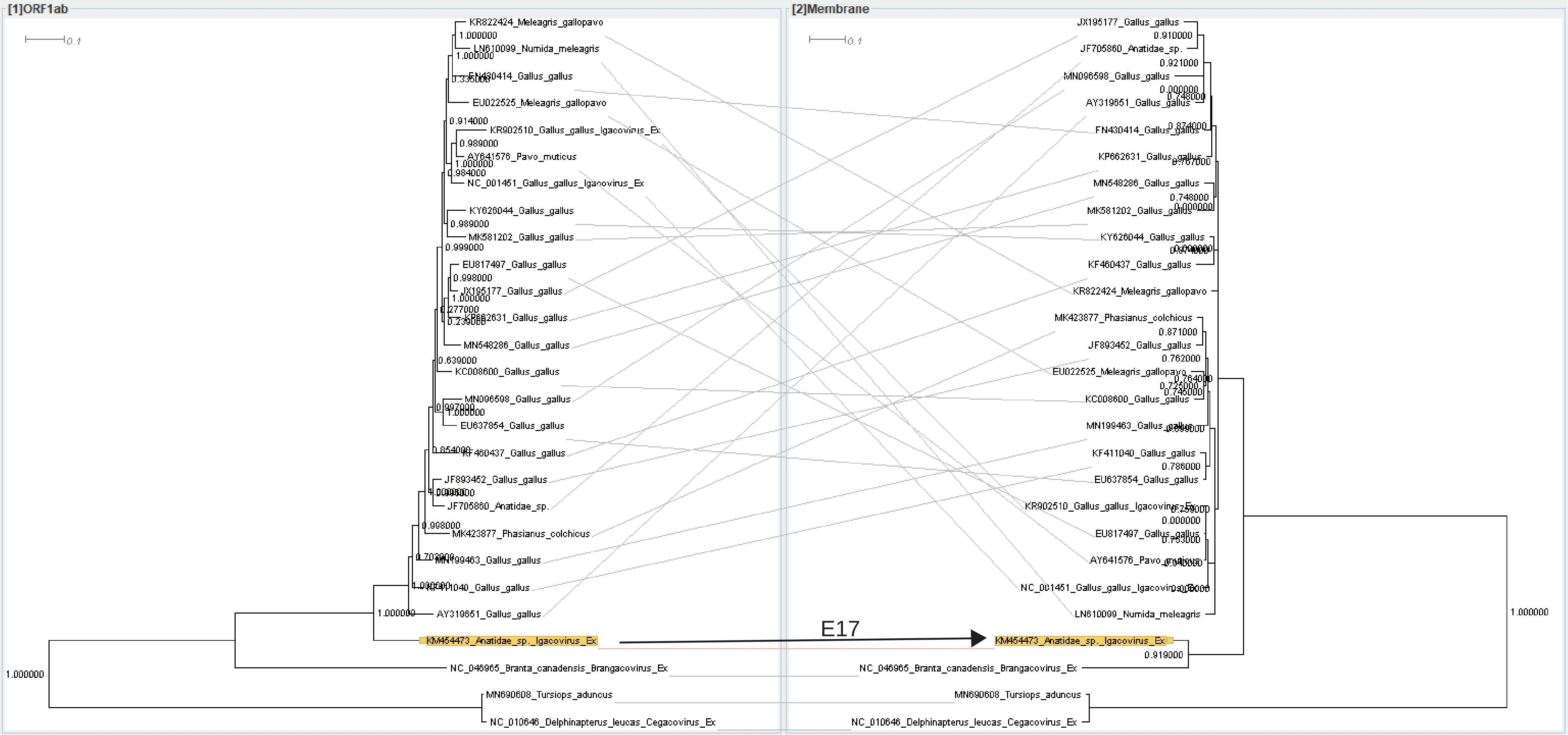

Supp. fig. 47: *Gammacoronavirus* ORF1ab - Membrane Bayesian Tanglegram (Event 17)

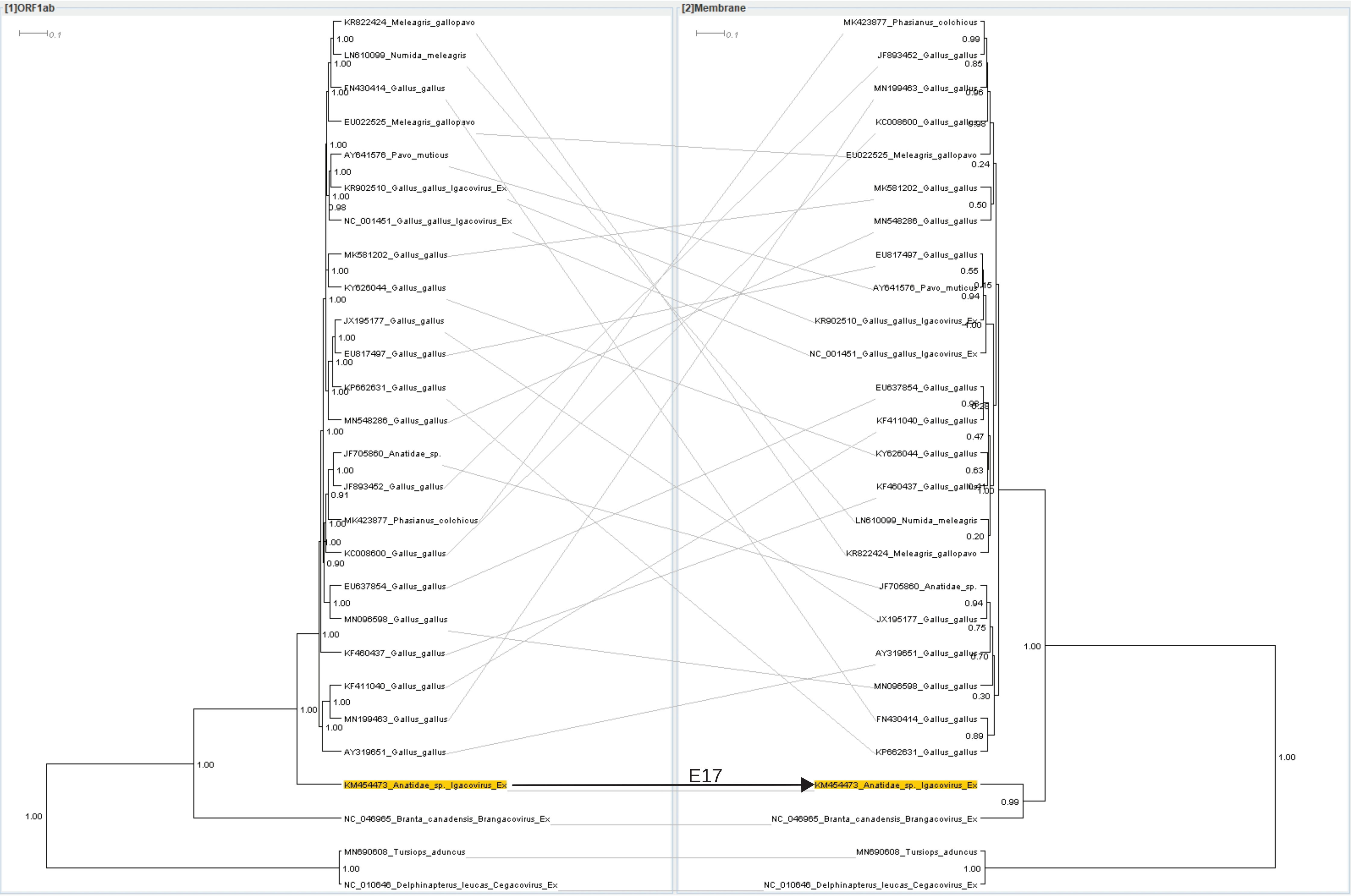

Supp. fig. 48: Recombination events 16-17 Simplot and Bootscan analyses

A) *Gammacoronavirus Igacovirus* Simplot:

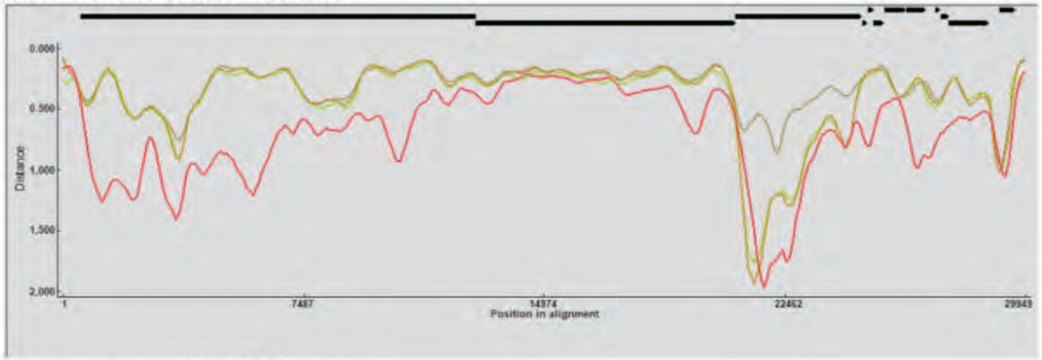

B) *Gammacoronavirus Igacovirus* Bootscan:

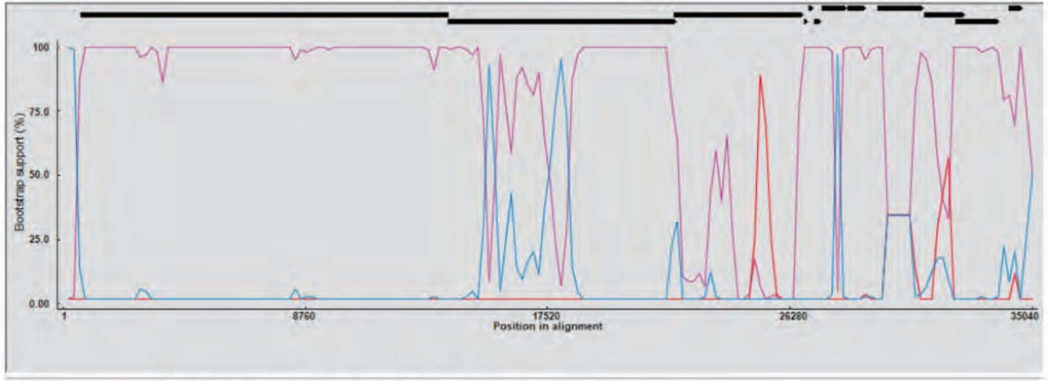

C) *Gammacoronavirus* recombinant *Igacovirus* ORF1ab vs Spike Tanglegram

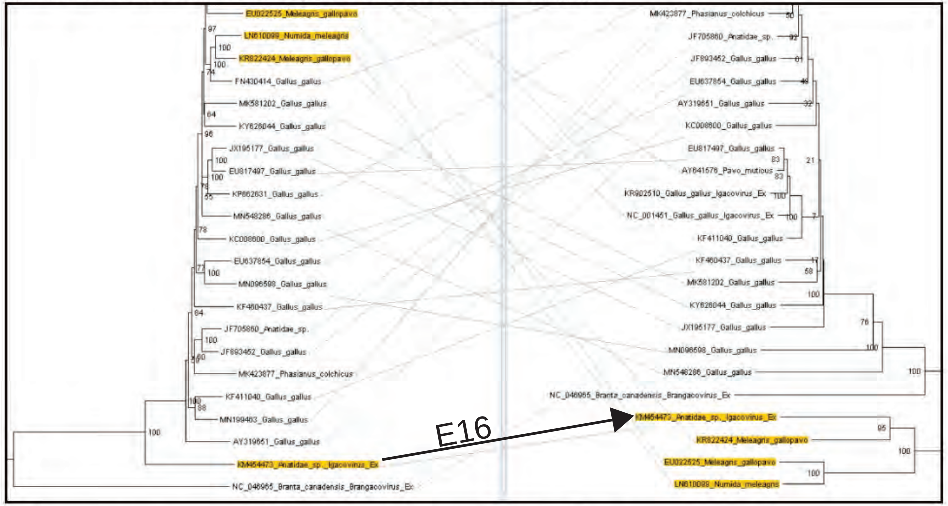

D) *Gammacoronavirus* recombinant *Igacovirus* ORF1ab vs Membrane Tanglegram

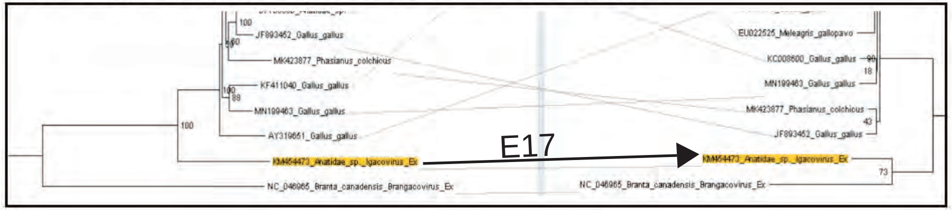

KM454473_Anatidae_sp._Igacovirus_Ex scanned against:

Simplot

- KR822424_Meleagris_gallopavo
- KR902510_Gallus_gallus_Igacovirus_Ex
- NC_001451_Gallus_gallus_Igacovirus_Ex
- NC_046965_Branta_canadensis_Brangacovirus_Ex

Bootscan

- KR902510_Gallus_gallus_Igacovirus_Ex
- NC_010646_Delphinapterus_leucas_Cegacovirus_Ex
- NC_046965_Branta_canadensis_Brangacovirus_Ex

Simplot params:
Window size:500
Step size:100

Bootscan params:
Window size:500
Step size:200
Bootstrap replicates:100
Pairwise distances

Supp. fig. 49: Event 17 CONSEL (ORF1ab WAGIG - Membrane WAGIG PhyML, aLRT, SPR)

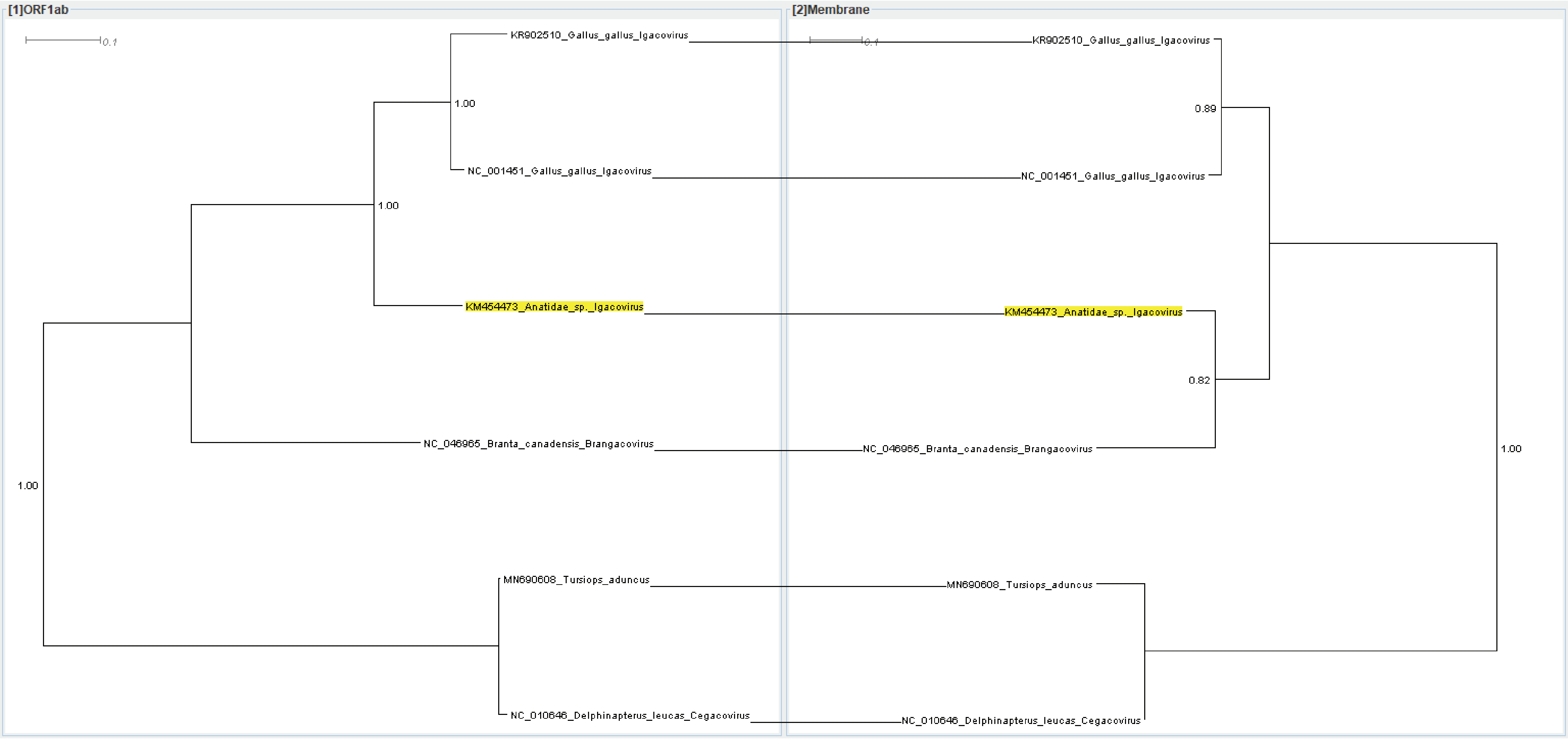

Trees	obs	au	np	bp	pp	kh	sh	wkh	wsh
Membrane	-67.7	1	1	1	1	1	1	1	1
ORF1ab	67.7	1.00E-05	1.00E-05	0	4.00E-30	0	0	0	0

Recombinant organisms in CONSEL analyses are colored yellow

Supp. fig. 50: *Gammacoronavirus* BioNJ Robinson-Foulds matrix

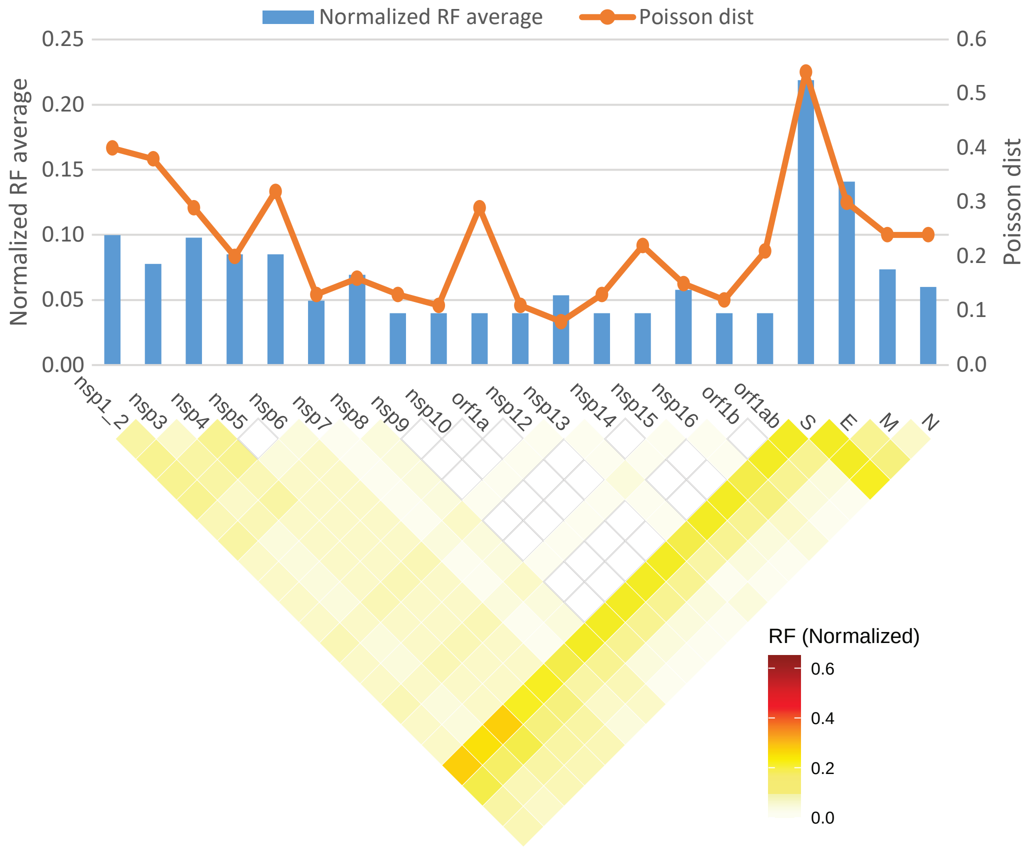

Gammacoronavirus BioNJ collapsed br. length 0.02
Poisson model, 500bs

Supp. fig. 51: *Gammacoronavirus* PhyML Robinson-Foulds matrix

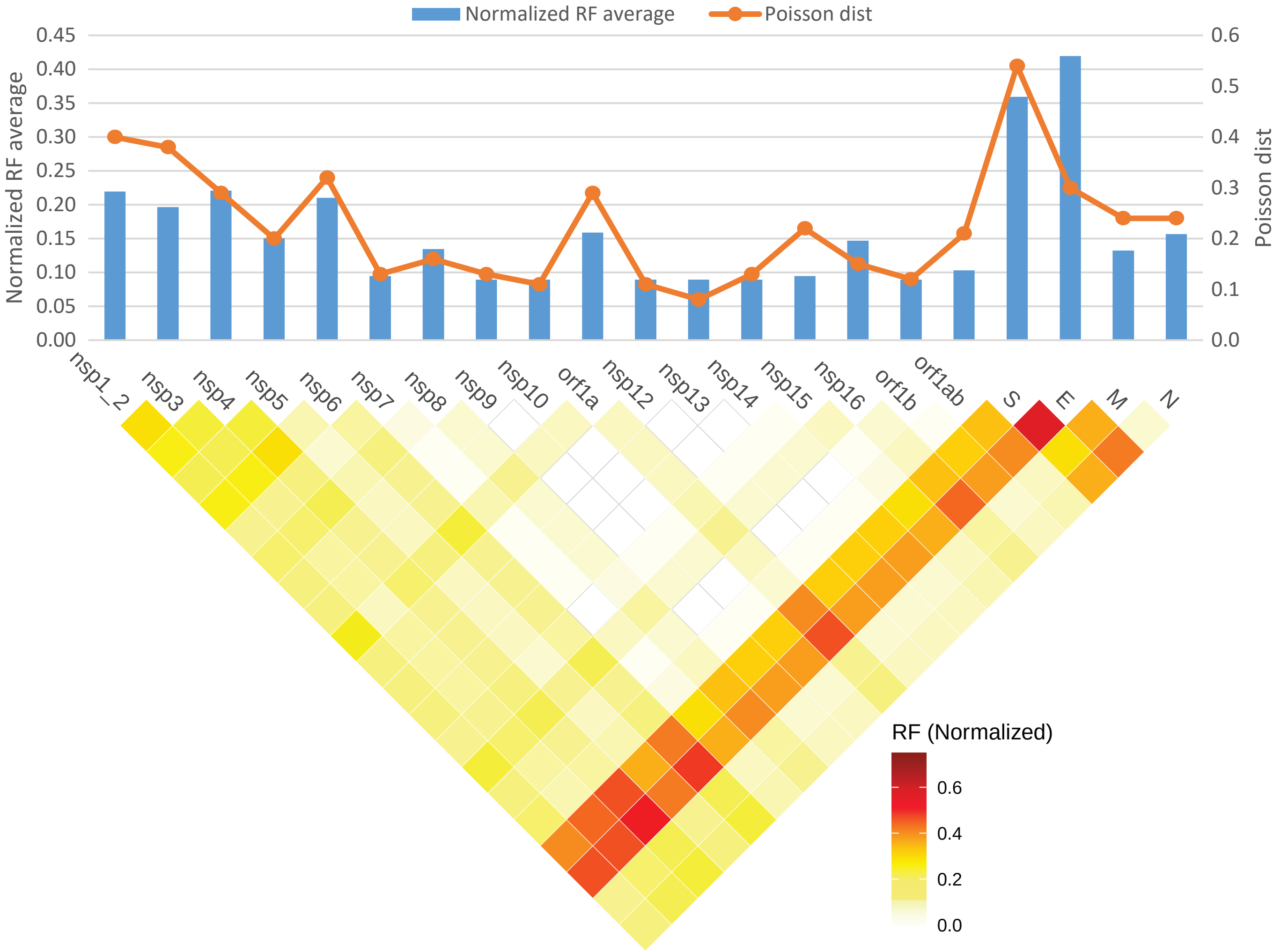

Gammacoronavirus PhyML collapsed br. length 0.02

PhyML model: JTT+G

Supplementary file 1 section 2, subsection 4

Recombination events for δ-CoVs

Recombinant *Buldecovirus* sequences are colored yellow

Phylogenetic incogruences are noted with arrows

Recombinant organisms in CONSEL analyses are colored yellow

- Event 18: *Buldecovirus* Spike
  This event happened at the common ancestor of the representative sequences LC364342, LC364343 and LC364344.

- Event 19: *Buldecovirus* Spike
  This event is about NC_016993 *Copsychus saularis.*

- Event 20: *Buldecovirus* Spike
  This event is about NC_016992 *Passer montanus.*

In events 18 - 20 five *Buldecoviruses* are no longer part of the main *Buldecovirus* group and are forming a monophyletic group that is sister group to *Herdecovirus* . In total they are considered three separate recombination events which are supported by all three trees (boostrap 88, aLRT 0.81, posterior probability 1). These events are also supported by Simplot, Bootscan and CONSEL.

- Event 21: *Deltacoronavirus* Spike
  This is an old recombination event that happened at the common ancestor of all *Deltacoronavirus-es*. δ-CoVs are grouping together with the *Alphacoronaviruses* A1 and A2. This event is also shown in Supp. fig. 1 and supported by all three trees (Bootstrap support 100, aLRT 1, posterior probability 1). This event is also supported by CONSEL.

Supp. fig. 52: *Deltacoronavirus* ORF1ab - Spike BioNJ Tanglegram (Events 18-20)

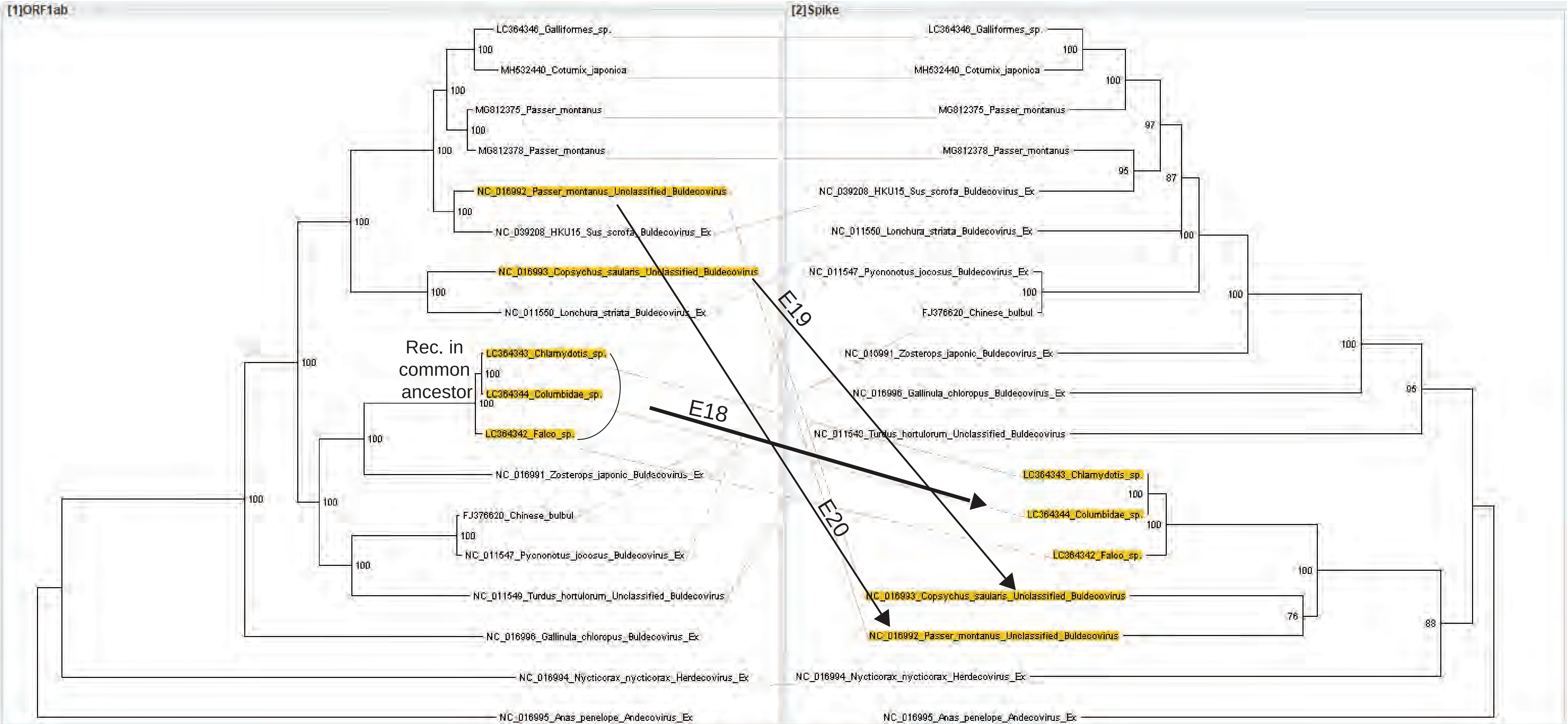

Supp. fig. 53: *Deltacoronavirus* ORF1ab - Spike PhyML Tanglegram (Events 18-20)

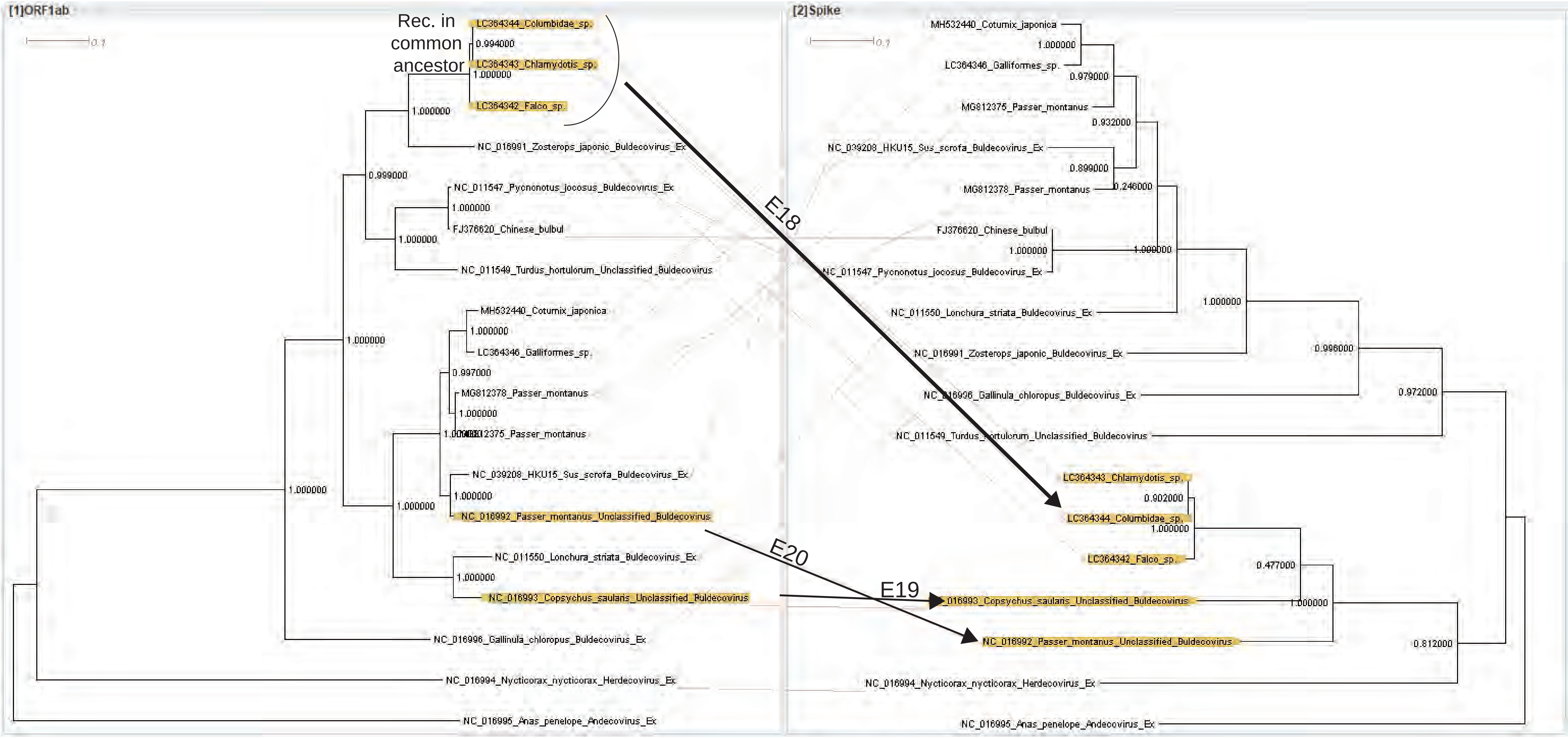

Supp. fig. 54: *Deltacoronavirus* ORF1ab - Spike Bayesian Tanglegram (Events 18-20)

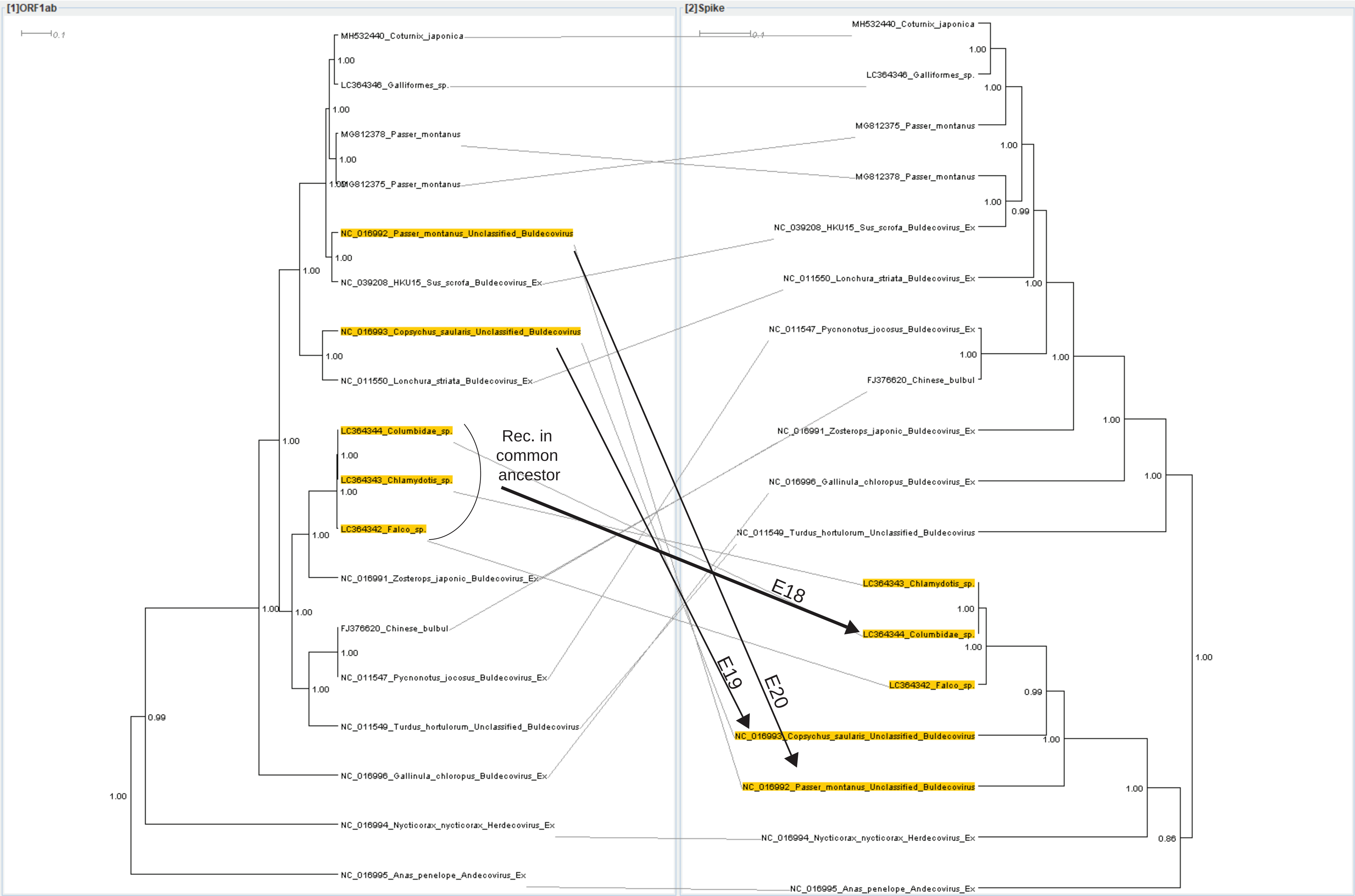

Supp. fig. 55: Recombination event 20 Simplot and Bootscan analyses

A) *Deltacoronavirus Buldecovirus* Simplot:

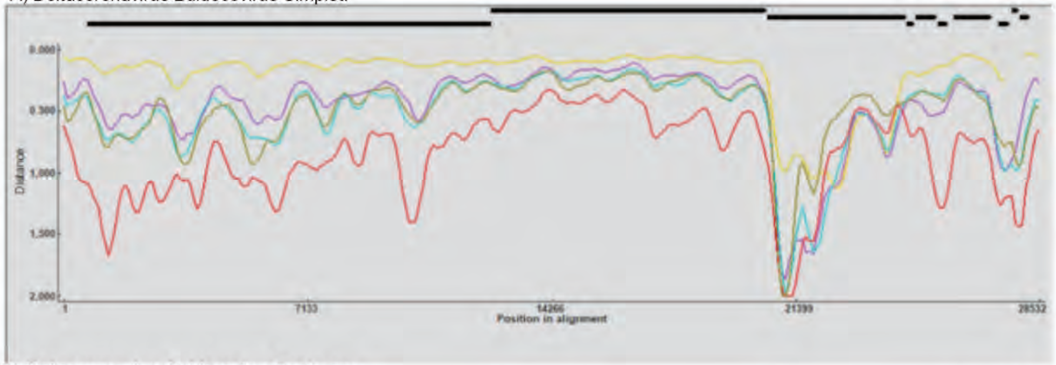

B) *Deltacoronavirus Buldecovirus* Bootscan:

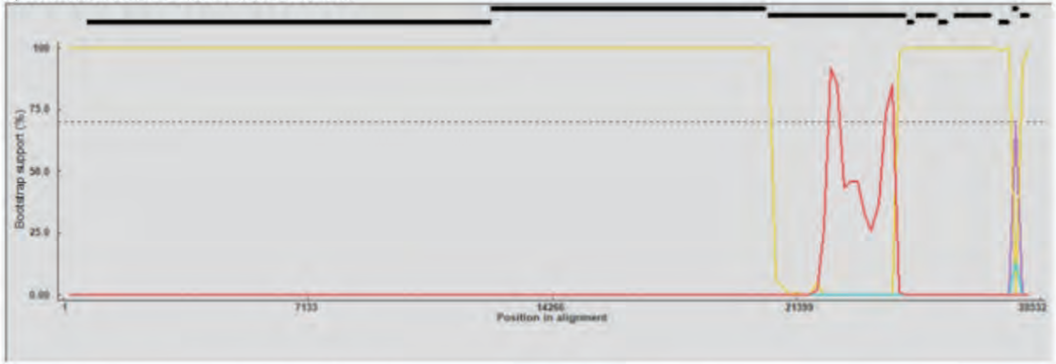

C) *Deltacoronavirus* recombinant *Buldecovirus* ORF1ab vs Spike Tanglegram

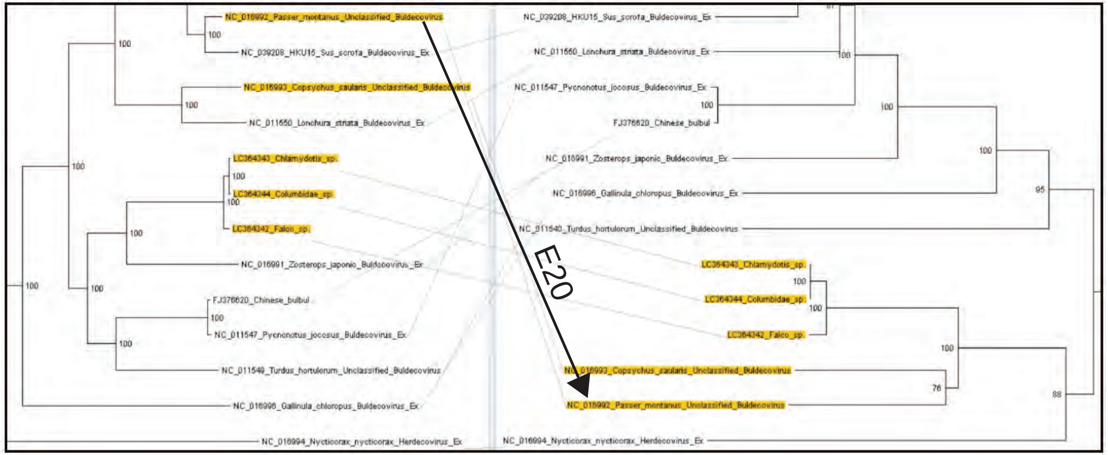

NC_016992_Passer_montanus_Unclassified_Buldecovirus scanned against:

- NC_011550_Lonchura_striata_Buldecovirus_Ex
- NC_039208_HKU15_Sus_scrofa_Buldecovirus_Ex
- NC_016991_Zosterops_japonicus_Buldecovirus_Ex
- NC_016994_Nycticorax_nycticorax_Herdecovirus_Ex
- LC364343_Chlamydotis_sp.

Simplot params:
Window size:500
Step size:100

Bootscan params:
Window size:500
Step size:200
Bootstrap replicates:100
Pairwise distances

Supp. fig. 56: Events 18-20 CONSEL (ORF1ab LGIG - Spike LGIG PhyML, aLRT, SPR)

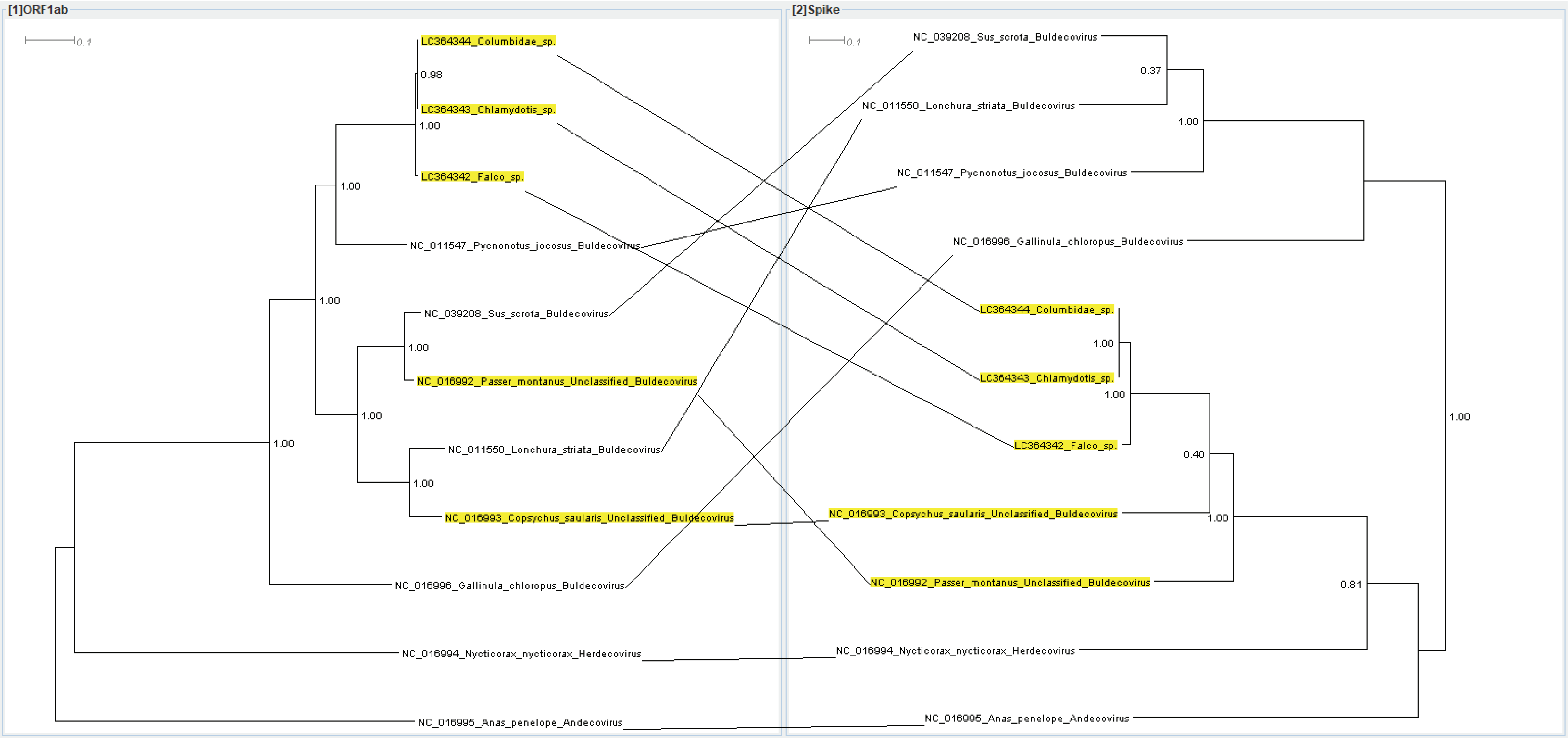

Tree	obs	au	np	bp	pp	kh	sh	wkh	wsh
Spike	-3991.7	1	1	1	1	1	1	1	1
ORF1ab	3991.7	8.00E-40	3.00E-15	0	0	0	0	0	0

Recombinant organisms in CONSEL analyses are colored yellow

Supp. fig. 57: *Deltacoronavirus* BioNJ Robinson-Foulds matrix

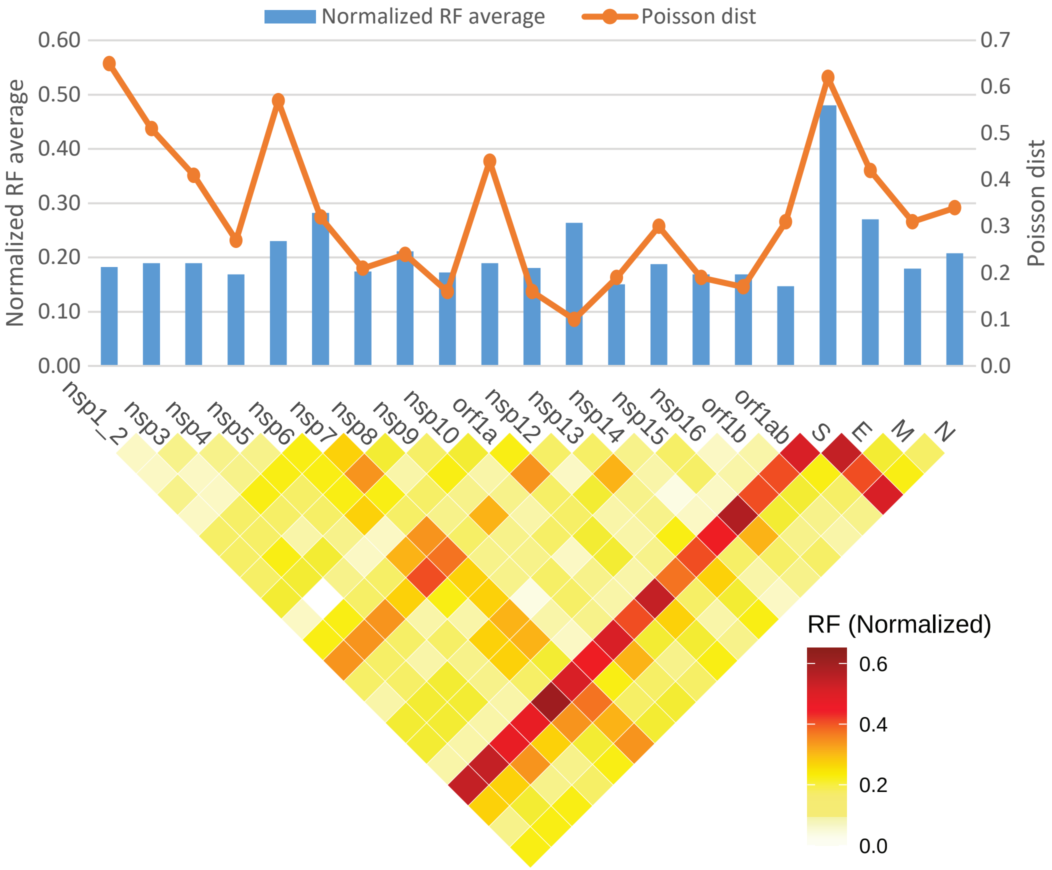

Deltacoronavirus BioNJ collapsed br. length 0.02
Poisson model, 500bs

Supp. fig. 58: *Deltacoronavirus* PhyML Robinson-Foulds matrix

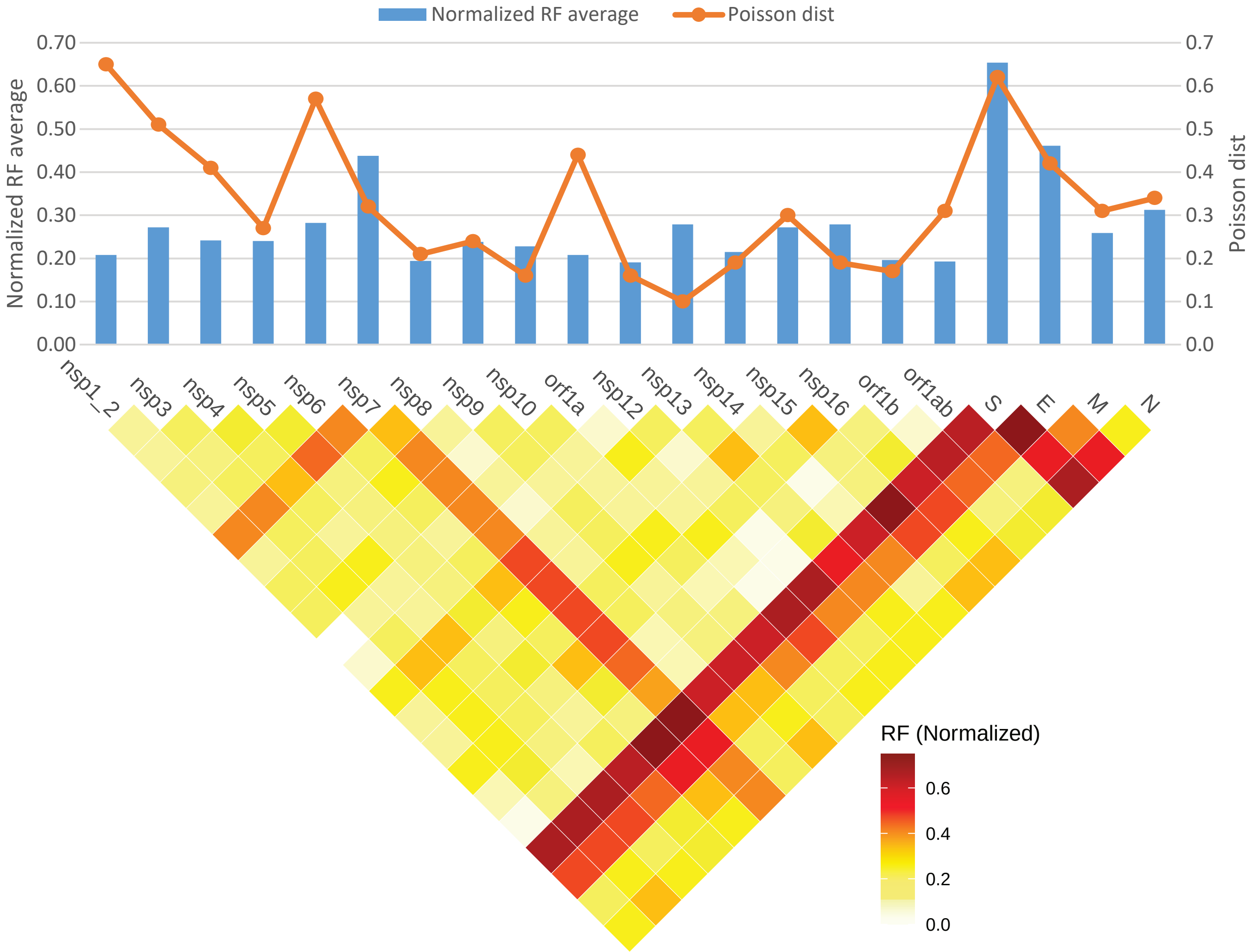

Deltacoronavirus PhyML collapsed br. length 0.02

PhyML model: LG+I+G

Supplementary file 1 Section 3

Recombination events among members of the same subgenus are not the focus of this study. Nevertheless, due to the emerging importance of *Sarbecoviruses*, we performed an analysis only for this subgenus. Within *Sarbecoviruses*, three main groups emerge, the SARS, SARS2 and a third group comprising of two *Rhinolophus* CoV genomes. All three groups have genome-wide p-distances among them ranging between 0.209 - 0.237. One *Rhinolophus sinicus* CoV genome (MG772933) from the SARS2 group, has obtained a genomic region from a member of the SARS group that spans between the end of nsp6 (pos. 11698) up to the beginning of nsp16 (pos. 20579). Thus it is conceivable that in the future, more recombinations between members of the three distinct *Sarbecovirus* groups may occur, giving rise to new distinct groups and possibly new *Sarbecovirus* epidemics with new clinical/epidemiological features. Recombination between the SARS and SARS2 groups or within each of the groups has already been reported (Yang et al., 2020) (Boni et al.,2020) (Lam et al., 2020)

The following Neighbour Joining trees were created from nucleotide alignments of each subregion
Kimura 2 parameter model, 500 bootstrap replicates. This is an intra-subgenus recombination event

The blue lines indicate the three different *Sarbecovirus* groups

Yang, Y., Yan, W., Hall, A.B., Jiang, X., Characterizing transcriptional regulatory sequences in coronaviruses and their role in recombination. Mol. Biol. Evol. (2020).

Boni, M.F. et al. Evolutionary origins of the SARS-CoV-2 sarbecovirus lineage responsible for the COVID-19 pandemic. Nat. Microbiol. **5,** 1408–1417 (2020).

Lam, T.T.-Y. et al. Identifying SARS-CoV-2-related coronaviruses in Malayan pangolins. Nature **583,** 282–285 (2020).

Supp. fig 59: Poisson distances of Sarbecovirus genomic regions against the other β-CoV subgenera

	nsp1	nsp2	nsp3	nsp4	nsp5	nsp6	nsp7	nsp8	nsp9	nsp10	nsp12	nsp13	nsp14	nsp15	nsp16	S	E	M	N
Hibecovirus	1.290	1.192	0.945	0.663	0.372	0.818	0.327	0.504	0.498	0.362	0.254	0.215	0.347	0.508	0.395	0.861	0.595	0.647	0.660
Nobecovirus	1.399	1.667	1.138	0.885	0.656	1.010	0.415	0.540	0.542	0.436	0.327	0.301	0.491	0.708	0.475	1.087	1.233	0.869	0.843
Merbecovirus	1.563	1.538	1.132	0.930	0.657	1.033	0.559	0.660	0.692	0.503	0.332	0.333	0.462	0.690	0.436	1.116	1.018	0.855	0.710
Embecovirus	1.623	1.884	1.240	0.894	0.717	1.188	0.725	0.739	0.746	0.630	0.411	0.390	0.545	0.716	0.438	1.150	1.613	0.934	1.008

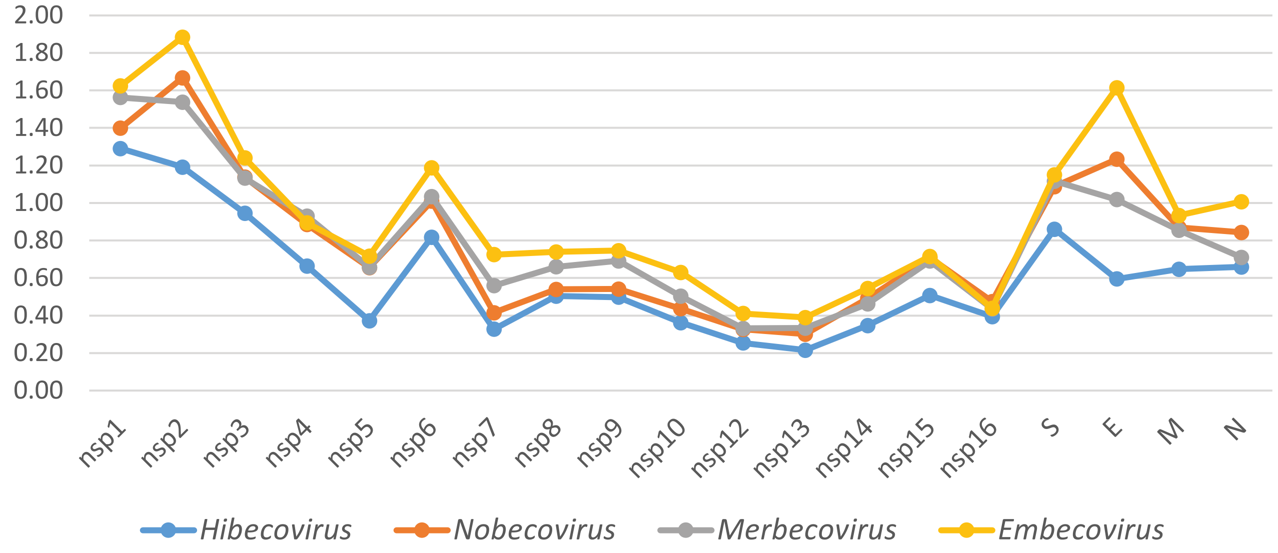

MEGA Parameters:
Protein alignments
Between group average Poisson distance
Uniform rates among sites
Pairwise deletion for missing data/gaps

Supp. fig. 60: *Sarbecovirus* ORF1ab - nsp7 BioNJ Tanglegram

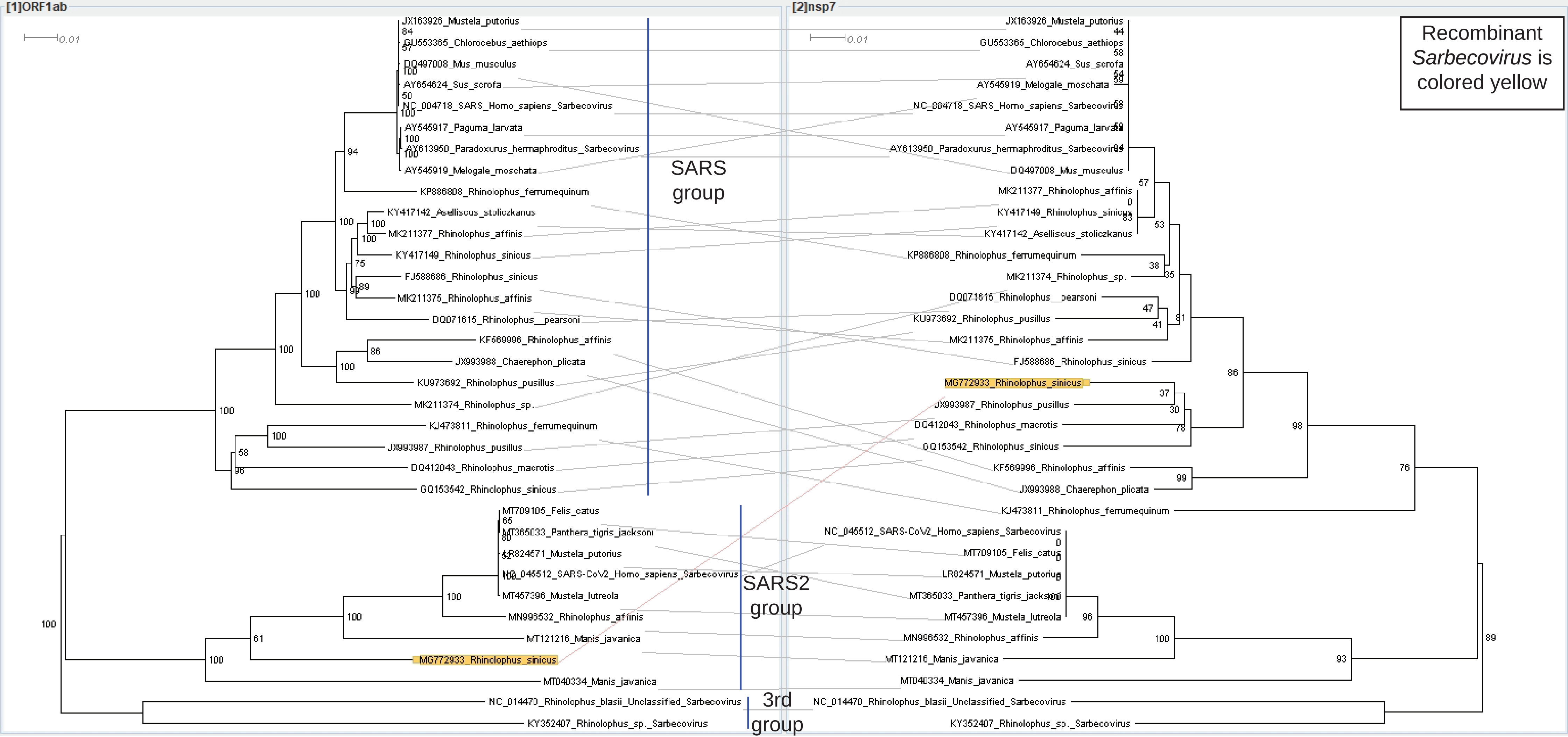

Supp. fig. 61: *Sarbecovirus* ORF1ab - nsp8 BioNJ Tanglegram

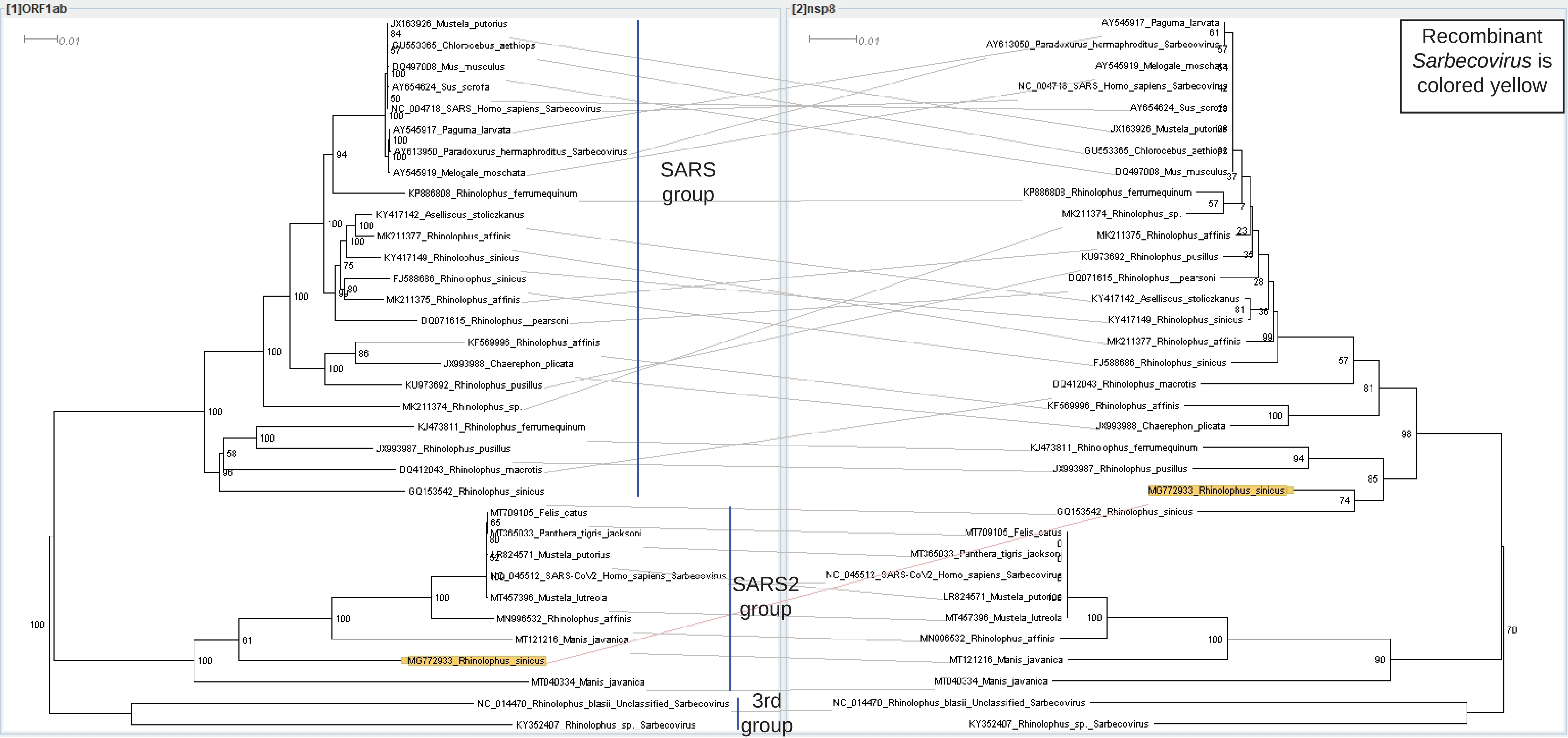

Supp. fig. 62: *Sarbecovirus* ORF1ab - nsp9 BioNJ Tanglegram

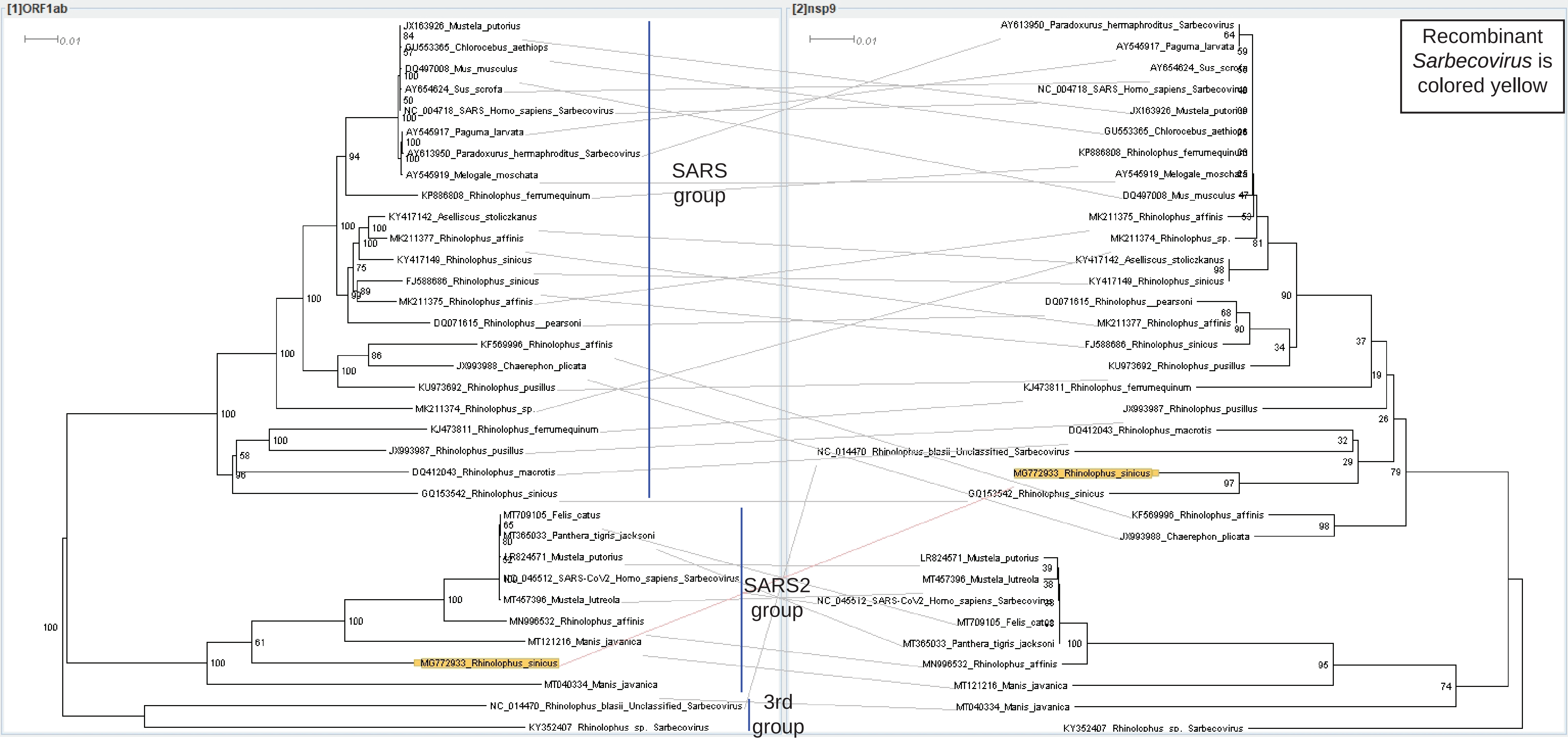

Supp. fig. 63: *Sarbecovirus* ORF1ab - nsp10 BioNJ Tanglegram

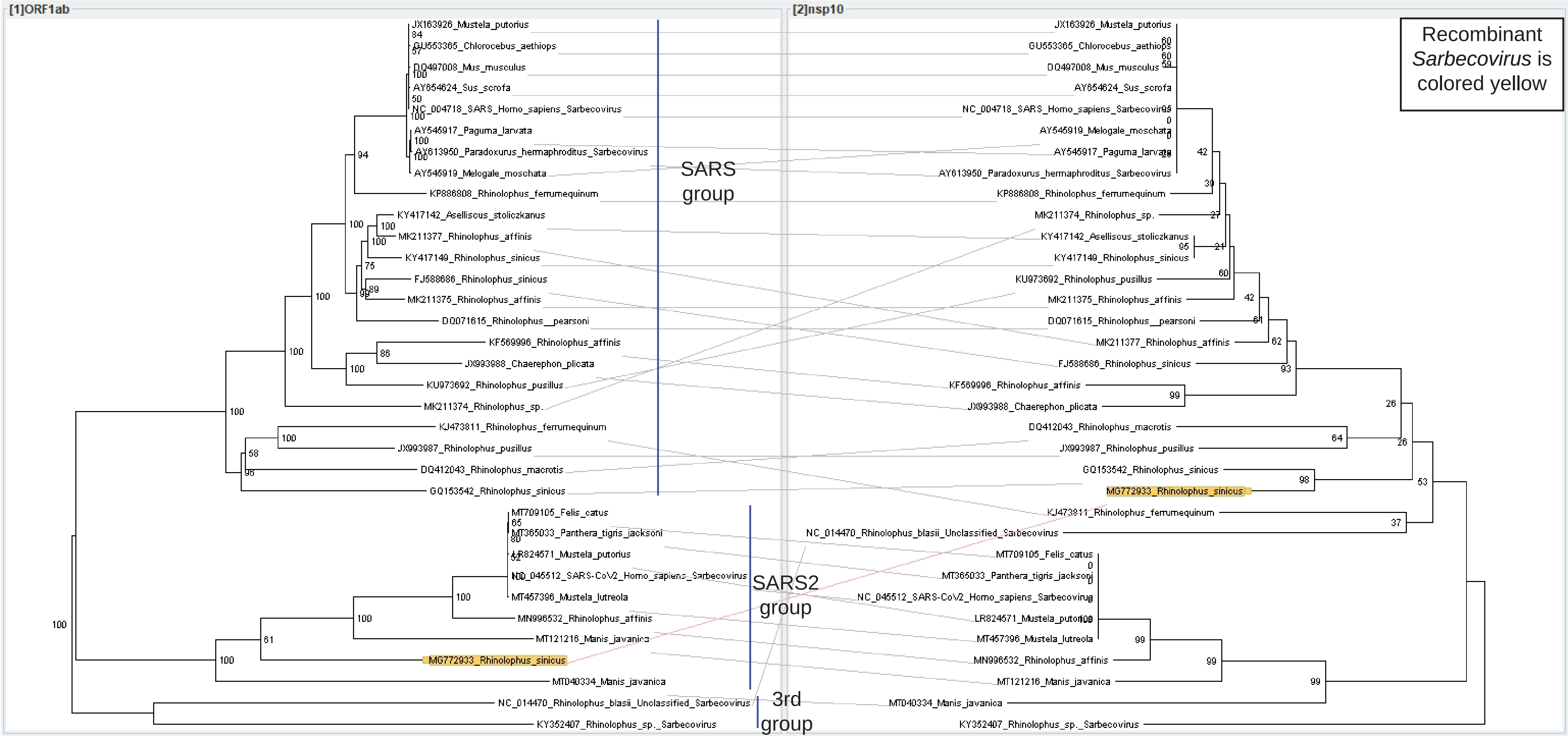

Supp. fig. 64: *Sarbecovirus* ORF1ab - nsp12 BioNJ Tanglegram

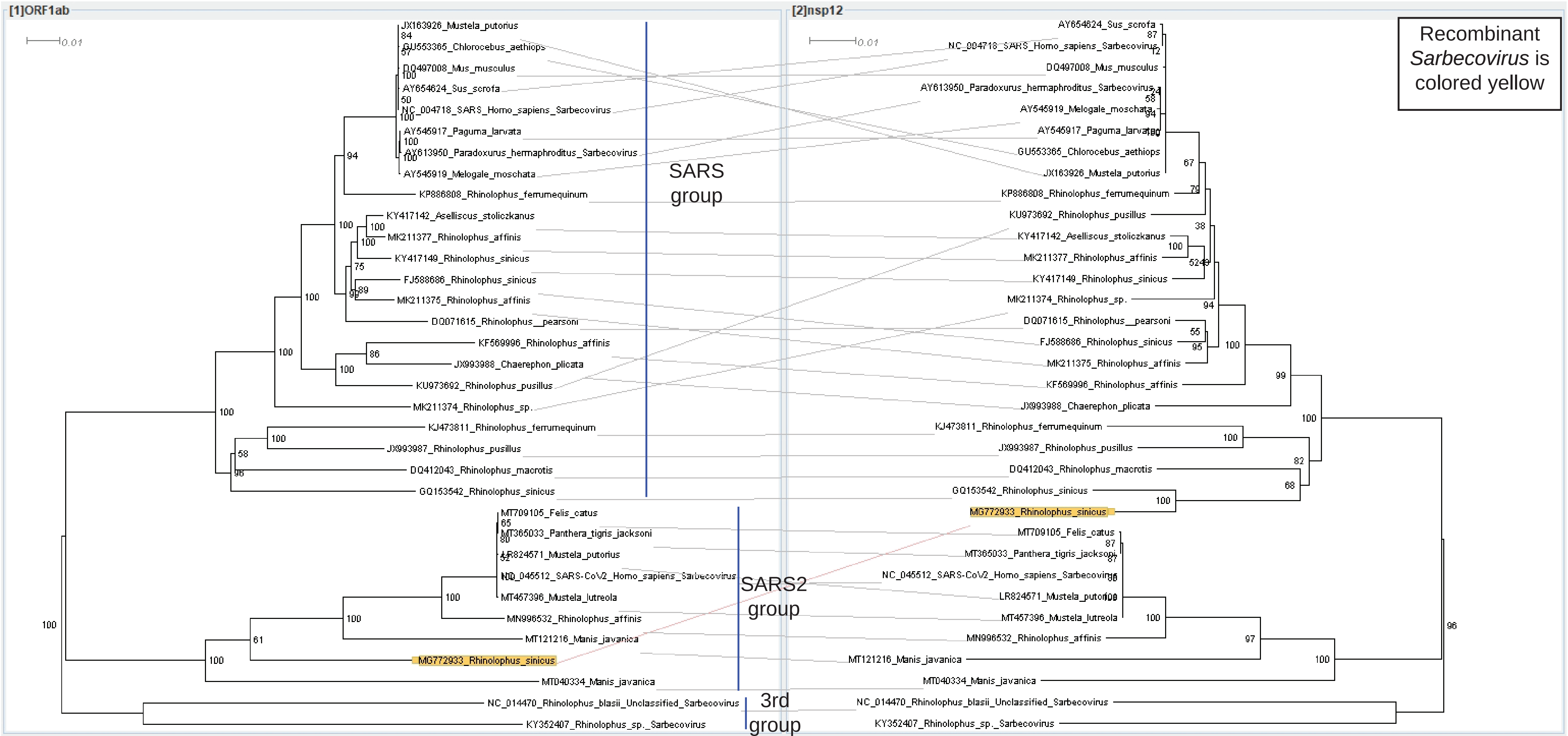

Supp. fig. 65: *Sarbecovirus* ORF1ab - nsp13 BioNJ Tanglegram

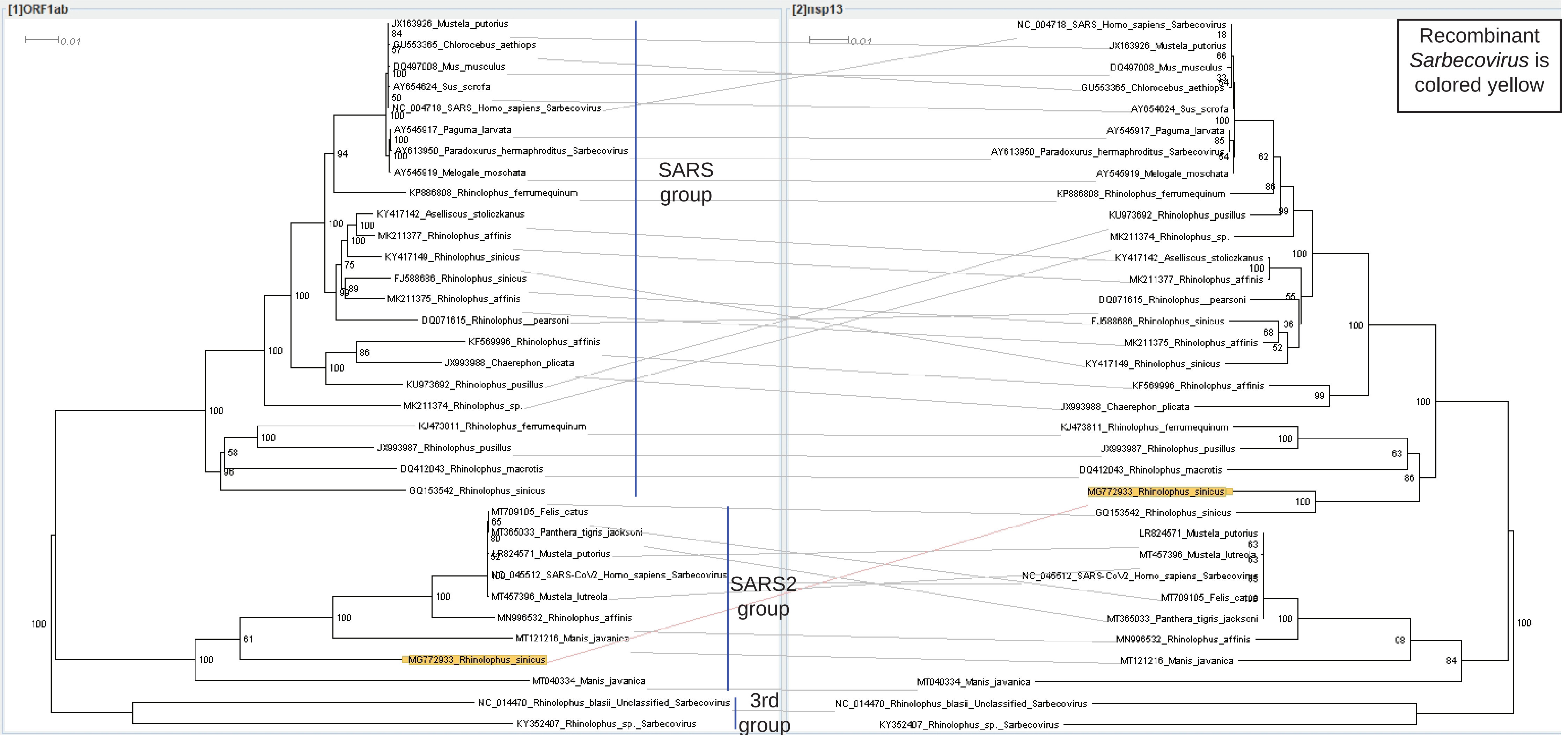

Supp. fig. 66: *Sarbecovirus* ORF1ab - nsp14 BioNJ Tanglegram

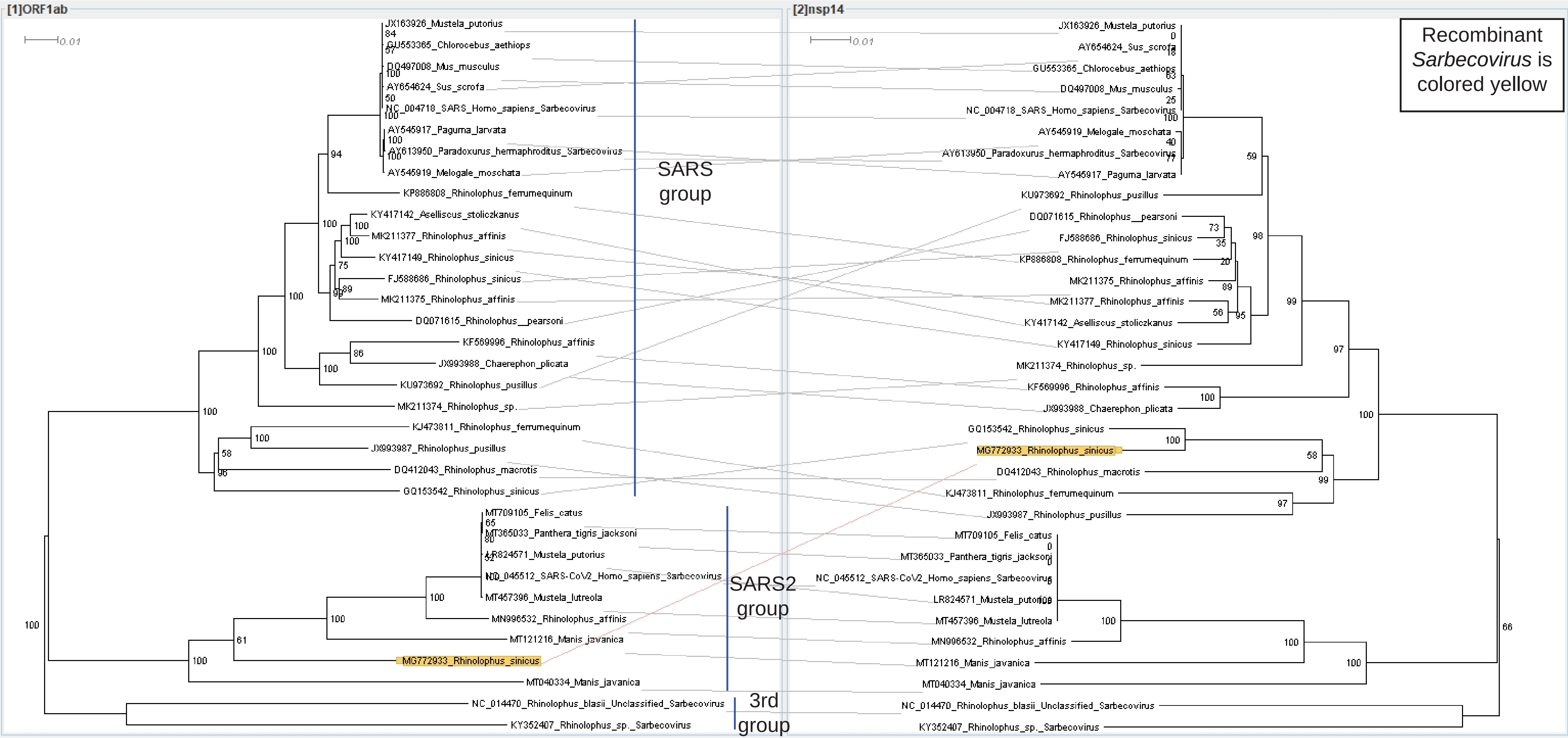

Supp. fig. 67: *Sarbecovirus* ORF1ab - nsp15 BioNJ Tanglegram

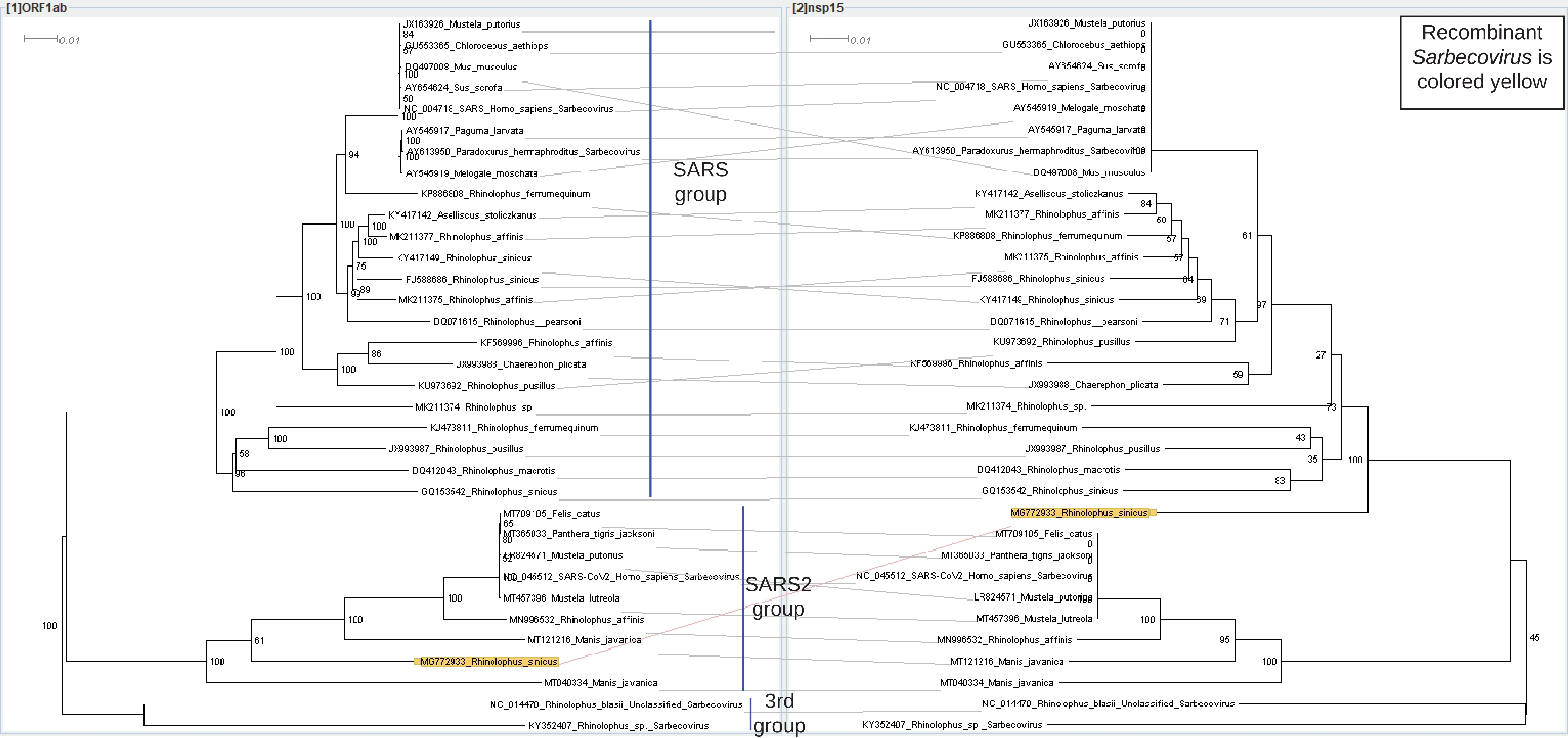

Supp. fig. 68: Recombinant *Sarbecovirus* Simplot and Bootscan

A) Sarbecovirus Simplot

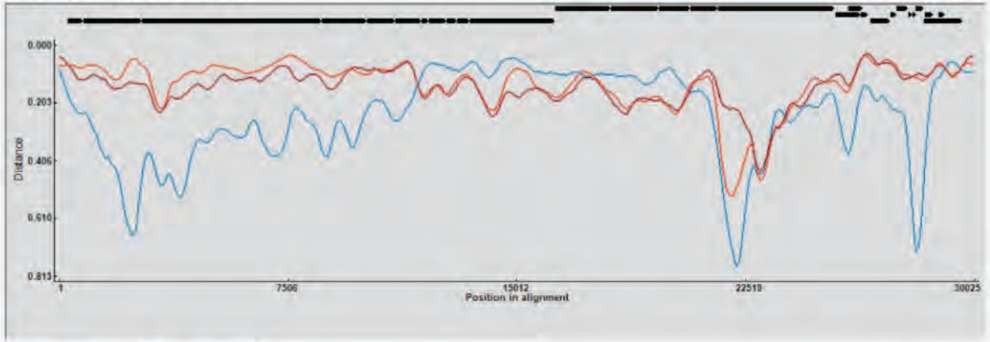

C) *Sarbecovirus* ORF1ab nsp7 Tanglegram

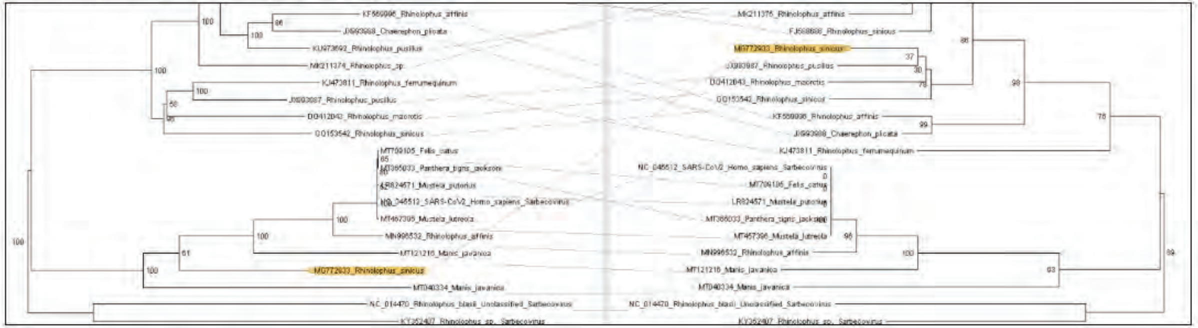

B) Sarbecovirus Bootscan

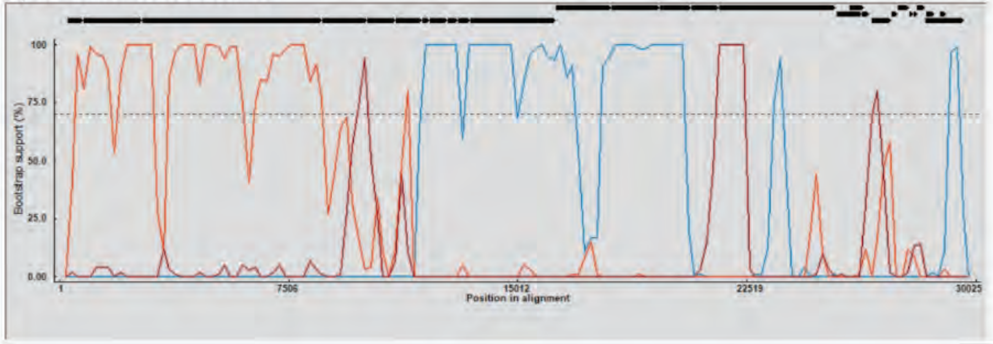

D) *Sarbecovirus* ORF1ab nsp15 Tanglegram

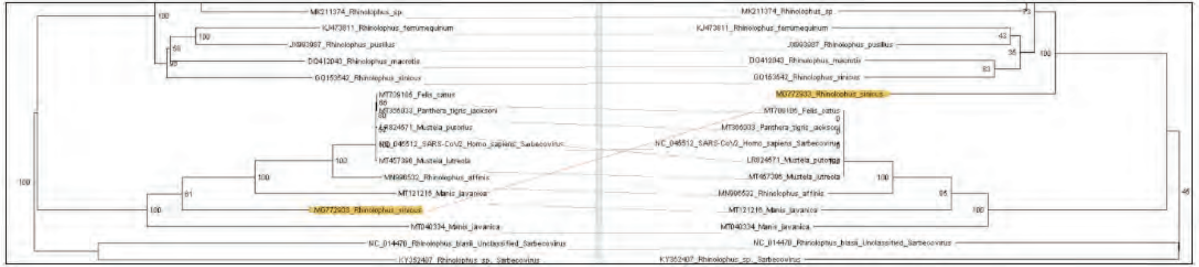

MG77293_Rhinolophus_sinicus scanned against:
NC_004718_SARS_Homo_sapiens_Sarbecovirus_Ex
NC_045512_SARS-CoV2_Homo_sapiens_Sarbecovirus
MT121216_Manis_javanica

Simplot params:
Window size:500
Step size:100

Bootscan params:
Window size:500
Step size:200
Bootstrap replicates:100
Pairwise distances
